# Cost-effectiveness analysis of guidelines for antihypertensive care in Finland

**DOI:** 10.1186/1472-6963-7-172

**Published:** 2007-10-24

**Authors:** Neill Booth, Antti Jula, Pasi Aronen, Minna Kaila, Timo Klaukka, Katriina Kukkonen-Harjula, Antti Reunanen, Pekka Rissanen, Harri Sintonen, Marjukka Mäkelä

**Affiliations:** 1 Tampere School of Public Health, University of Tampere, Tampere, Finland; 2 Department of Health and Functional Capacity, National Public Health Institute, Helsinki, Finland; 3 Finnish Office for Health Technology Assessment (FinOHTA), National Research and Development Centre for Welfare and Health (STAKES), Helsinki, Finland; 4 Department of Public Health, University of Helsinki, Helsinki, Finland; 5 Paediatric Research Centre, Tampere University Hospital and University of Tampere, Tampere, Finland; 6 Research Department, Social Insurance Institution, Helsinki, Finland; 7 UKK Institute for Health Promotion Research, Tampere, Finland; 8 University of Copenhagen, Copenhagen, Denmark

## Abstract

**Background:**

Hypertension is one of the major causes of disease burden affecting the Finnish population. Over the last decade, evidence-based care has emerged to complement other approaches to antihypertensive care, often without health economic assessment of its costs and effects. This study looks at the extent to which changes proposed by the 2002 Finnish evidence-based Current Care Guidelines concerning the prevention, diagnosis, and treatment of hypertension (the ACCG scenario) can be considered cost-effective when compared to modelled prior clinical practice (the PCP scenario).

**Methods:**

A decision analytic model compares the ACCG and PCP scenarios using information synthesised from a set of national registers covering prescription drug reimbursements, morbidity, and mortality with data from two national surveys concerning health and functional capacity. Statistical methods are used to estimate model parameters from Finnish data. We model the potential impact of the different treatment strategies under the ACCG and PCP scenarios, such as lifestyle counselling and drug therapy, for subgroups stratified by age, gender, and blood pressure. The model provides estimates of the differences in major health-related outcomes in the form of life-years and costs as calculated from a 'public health care system' perspective. Cost-effectiveness analysis results are presented for subgroups and for the target population as a whole.

**Results:**

The impact of the use of the ACCG scenario in subgroups (aged 40–80) without concomitant cardiovascular and related diseases is mainly positive. Generally, costs and life-years decrease in unison in the lowest blood pressure group, while in the highest blood pressure group costs and life-years increase together and in the other groups the ACCG scenario is less expensive and produces more life-years. When the costs and effects for subgroups are combined using standard decision analytic aggregation methods, the ACCG scenario is cost-saving and more effective.

**Conclusion:**

The ACCG scenario is likely to reduce costs and increase life-years compared to the PCP scenario in many subgroups. If the estimated trade-offs between the subgroups in terms of outcomes and costs are acceptable to decision-makers, then widespread implementation of the ACCG scenario is expected to reduce overall costs and be accompanied by positive outcomes overall.

## Background

### Rationale and objectives of the study

Despite the increasing use of evidence-based guidelines over the last decade to complement other approaches to care, there appears to be a relative dearth of English-language cost-effectiveness analyses of such guidelines ([1-11]). There are numerous possible approaches to cost-effectiveness analysis (CEA) in the field of antihypertensive care (see, e.g., [12-22]), mainly addressing questions such as 'whom to treat' and 'how to treat'. A literature database search strategy (see Additional file 1, Table 1) revealed no CEAs that have been carried out concerning broad alternative scenarios for antihypertensive care as outlined in evidence-based guidelines. Therefore, we undertook a cost-effectiveness analysis to evaluate the relative impact of the hypothetical application of two scenarios on the costs and effects of the prevention, diagnosis, and treatment of hypertension in Finland. Based on the 2002 evidence-based Antihypertensive Current Care Guideline (ACCG) [23], the ACCG scenario is compared with a prior clinical practice (PCP) scenario. For a description of the development process for the Finnish Current Care Guidelines see Additional file 2.

**Table 1:** Differences between the two approaches to the prevention, diagnosis, and treatment of hypertension.

**Clinical practice according to the ACCG**	**Clinical practice prior to the publication of the ACCG**
**prevention**	**prevention**
systematic counselling on health-related lifestyle choices if SBP is 130–139 mmHg and/or DBP 85–89 mmHg	a somewhat non-systematic approach
**diagnosis**	**diagnosis**
- BP measurements performed according to guideline specifications	- variations in BP measurement practices
- calculation of CHD risk profiles	- other CHD risk factors not fully incorporated
**treatment**	**treatment**
provide lifestyle counselling and considered initiation of pharmacological therapies with a stepwise approach	commonly pharmacological therapy, often without lifestyle counselling

The ACCG and PCP scenarios differ in the types of care they include and, hence, in the clinical outcomes expected to result from each scenario. We use a combination of individual-level data (i.e., observed and recorded information on a representative population sample of individuals) with data representative of the whole population (i.e., population data from national registers) to calculate expected outcomes in each scenario using decision analytic modelling (see Additional file 3, Figure 1).

**Figure 1:**
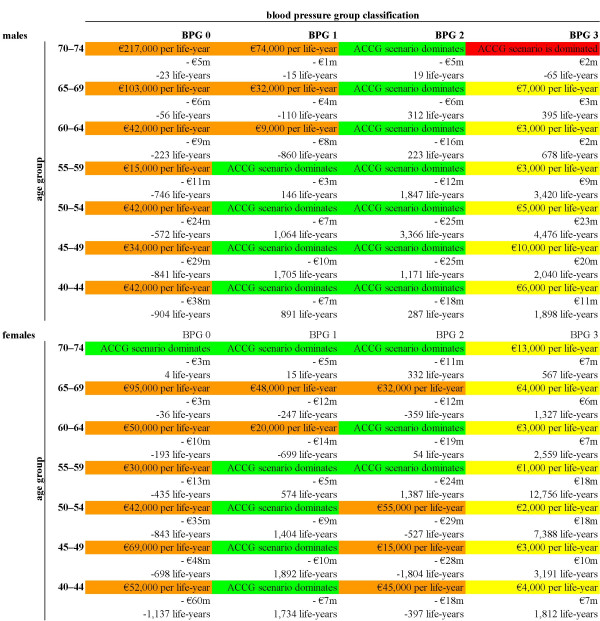
Base case incremental analysis for both genders, by age group and blood pressure group.

The ACCG is aimed primarily at providing health care professionals with guidance concerning the prevention, diagnosis, or treatment of hypertension in individuals. The objective of this cost-effectiveness study is to highlight some of the opportunity costs of the two scenarios in undertaking antihypertensive care in the longer term and at a national level (see Additional file 1, Table 2 for details of terminology such as opportunity costs). This research is intended mainly for members of the various bodies and organisations responsible for the selection and implementation of publicly funded health care technologies.

**Table 2:** Classification of blood pressure: If SBP and DBP fell into different groups, the individual was classified in the higher group.

	systolic blood pressure (in mmHg)		diastolic blood pressure (in mmHg)
BPG 0	< 130	and	< 85
BPG 1	130–139	and/or	85–89
BPG 2	140–159	and/or	90–99
BPG 3	≥160	or	≥100

### Antihypertensive care scenarios

Some important differences between antihypertensive care according to the 2002 antihypertensive Current Care guideline and prior clinical practice are shown in Table 1. The ACCG scenario refers to the hypothetical application of only part of the ACCG and the PCP scenario refers to the hypothetical application of observed PCP. These scenarios are constructed to form part of a decision analytic model. The blood pressure groups (BPGs) used in this study are presented in Table 2. The two scenarios each involve particular combinations of a variety of therapeutic interventions, as shown in Table 3.

**Table 3:** Main operationalised differences between the PCP and ACCG scenarios.

**ACCG scenario**	Monitoring	lifestyle counselling	single antihypertensive drug	two antihypertensive drugs	three antihypertensive drugs
BPG 0	Yes	No	No	No	No
BPG 1	No	Yes	No	No	No
BPG 2	No	Yes	Possible*	Possible	Possible
BPG 3	No	Yes	Yes	Possible	Possible
**PCP scenario**	monitoring	lifestyle counselling	single antihypertensive drug	two antihypertensive drugs	three antihypertensive drugs
BPG 0	Possible	No	Possible	Possible	Possible
BPG 1	Possible	No	Possible	Possible	Possible
BPG 2	Possible	No	Possible	Possible	Possible
BPG 3	Possible	No	Possible	Possible	Possible

* In this table, 'Possible' refers to the potential use of interventions. In the ACCG scenario, this refers to the fact that treatment with antihypertensive pharmacological therapy can be considered in this case. For the PCP scenario, data from H2000 suggest that use of antihypertensive pharmacological therapy did occur in all BP groups, as did monitoring.

The most important assumed differences between the two scenarios are the following: 1) the frequency and type of BP measurement, 2) the use of coronary risk assessment, 3) the recommended therapeutic choices – especially – the preventative role of lifestyle counselling [23]. For example, in line with the ACCG, diagnostic BP measurement under the ACCG scenario consists of four sets of duplicate SBP and DBP measurements within a specified period of time (if the screening SBP is 140 mmHg or more or the screening DBP is 90 mmHg or more as averaged over two readings) [24]. In contrast, the PCP scenario is assumed to include four blood pressure measurements per year, see Additional file 4, Table 1. The use of coronary risk assessment tables [25] is advocated in the ACCG and modelled in the ACCG scenario, but such tables are assumed not to be used under the PCP scenario. The therapeutic choices for different BP groups under the PCP and ACCG scenarios differ as shown in Table 3, and the differences in the pharmacological therapies between the two scenarios are shown the additional material, see Additional file 1, Table 3. The lifestyle counselling intervention is assumed to be applicable to all individuals in the ACCG scenario, except those in the lowest-numbered BP group, BPG 0. It is assumed that lifestyle counselling is not used under the PCP scenario.

The PCP and ACCG scenarios are hypothetically applied to the individuals in the study population as if antihypertensive care was being initiated. The two scenarios are applied only to individuals without concomitant cardiovascular disease or diabetes. That is, the analysis of the two scenarios is specific to individuals without diagnoses of diabetes, coronary heart disease (CHD), or cerebrovascular events (CVEs). In addition, the two scenarios are restricted to individuals aged 40–74. Almost 1.5 million Finns, out of a total Finnish population of almost 5.2 million, fall into this category. Among this target population of 1.5 million Finns, over 70% of males and over 60% of females have elevated blood pressure (SBP exceeding 130 mmHg or DBP over 85 mmHg). The 'do nothing' comparator was assumed not to be a reasonable alternative in the context of antihypertensive care in a Western European society.

## Methods

### Modelling

For the purposes of economic evaluation, a decision model with Markov cycle sub-trees was built [26]. The sub-trees consist of 11 Markov states, which describe the major health-related outcomes and costs associated with antihypertensive care (see Additional file 3, Figure 3). For the basic structure of the decision tree see Additional file 3, Figure 3. The Markov cycle duration was set at five years, the minimum time horizon of the model was 10 years, and the maximum time horizon was 40 years. Progression between states is represented by transition probabilities (see Additional file 1, Table 5). Transitions between states are determined by the use of both epidemiological study data and published analyses of clinical trial data. Results are presented as incremental cost-effectiveness ratios (ICERs).

**Table 4:** Yearly costs of pharmacological therapies* used in the PCP scenario, rounded to the nearest euro, by gender (2001 prices).

Pharmacological subgroup	ATC code	male	female
hydrochlorothiazide or trichlormethiazide and potassium-sparing agents	C03EA	40	42
beta-blocking agents	C07A	145	136
combination of metoprolol or bisoprolol and thiazides	C07B	141	141
atenolol or metoprolol and other antihypertensives	C07F	285	275
calcium channel blockers	C08	245	228
ACE inhibitors	C09A	203	192
combination of ACE inhibitors and diuretics	C09BA	233	228
combination of ACE inhibitors and calcium channel blockers	C09BB	349	348
angiotensin II subtype 1 receptor antagonists	C09C	251	250
combination of angiotensin II subtype 1 receptor antagonists and diuretics	C09D	271	274

*(weighted average of the different pharmacological subgroups)

**Figure 2:**
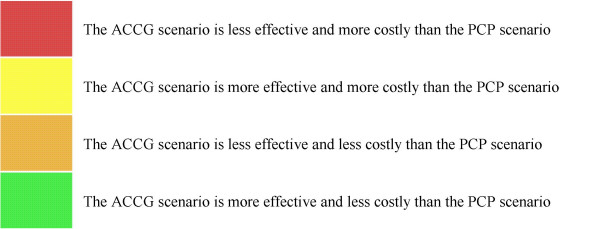
Key for figures 1, 3, and 4.

**Figure 3:**
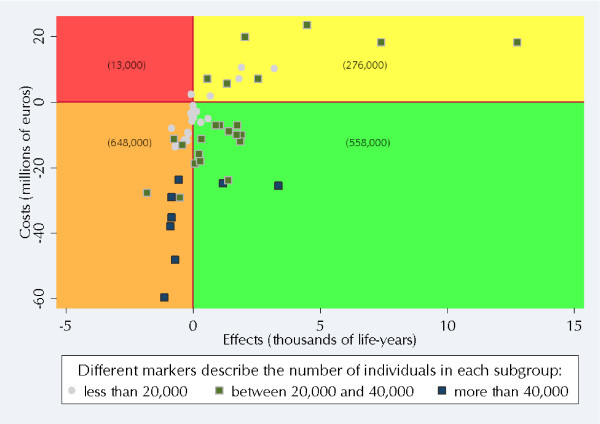
Subgroup results on the cost-effectiveness plane.

To facilitate modelling, the population was divided by gender and into seven age bands, each five years wide. The model follows cohorts of individuals (40–44, 45–49, 50–54, 55–59, 60–64, 65–69, and 70–74) and calculates costs and outcomes until members of the cohort exceed the age of 80. Results are given as incremental cost-effectiveness ratios in 56 age-, gender-, and BP-stratified subgroups as well as being aggregated over all subgroups.

A rate of discount of 5% was used for both costs and effectiveness in the base case analysis as well as 0% in sensitivity analysis in accordance with the guidelines of the Finnish Ministry of Social Affairs and Health [27]. In addition, one-way sensitivity analyses were carried out by changing the probabilities of regimen switching (only applicable in the ACCG scenario), the distribution of first-line therapy (only applicable in the ACCG scenario), the costs of medication, the costs of lifestyle counselling (only applicable in the ACCG scenario), the costs associated with morbidity, and the effects of antihypertensive care on BP. Sensitivity analysis was also carried out by attaching to the Markov states health-related quality of life (HRQL) weights as measured by the 15D instrument [28]. For further details of the sensitivity analyses, see Additional file 5.

Statistical analysis was carried out using the R statistical package [29] and version 6.12 of the SAS System [30].

### Population data

Data were available from the National Health 2000 Health Examination Survey (H2000), a two-stage stratified cluster sample undertaken in 2000–2001 (which included 8,028 persons aged 30 years and over and was representative of all people of that age in Finland) [31]. These data were used to provide prevalence estimates for BP groups in the population, stratified by age and gender, as well as a means for calculation of hypothetical treatment effects; an estimate of the use of antihypertensive medication under PCP; and the proportion of the population, stratified by age group and gender, with a risk of CHD within the next 10 years of ≥ 20% by applying a risk estimation equation [32]. In addition, the HRQL weights of the Markov states were derived from these data.

From the entire H2000 dataset, only data on individuals aged between 40 and 79 were utilised, as the expected observable frequency of events in other age groups, in terms of morbidity and mortality, was deemed insufficient. Further, the dataset was restricted to those individuals who have had their BP measured and who have no history of cardiovascular or related illnesses according to H2000 study. That is, the sample was restricted to individuals without diagnoses of diabetes, coronary heart disease (CHD), or cerebrovascular events (CVEs). The resultant sample size was 3,188. Almost 15% of individuals in this sample were recorded as using antihypertensive medication. For these individuals, prior to allocation to one of the four BP groups, the recorded SBP was increased by 10 mmHg and DBP by 6 mmHg. The same increase was applied regardless of BP group (see Additional file 1, Table 6). This simplified adjustment was undertaken because information for apportioning prior treatment effects more precisely was not available at the time of this study.

**Table 5:** Yearly costs of pharmacological therapies used in the ACCG scenario, rounded to the nearest euro, by gender (2001 prices).

Pharmacological substance	ATC code	male	female
hydrochlorothiazide	C03AA03	22	24
bisoprolol	C07AB07	112	107
nifedipine	C08CA05	243	227
enalapril	C09AA02	192	181
candesartan	C09CA06	235	237

Due to the relatively small sample size in the 70–74 age group, where necessary, it was assumed that the 75–79 age group also is representative of the 70–74 age group. Expansion weights calculated by Statistics Finland for the H2000 sample were applied to provide estimates of target population sizes at the national level in the 56 subgroups, with these subgroups representing approximately 1.5 million Finns (i.e., 62% of the Finnish population between the ages of 40 and 74), of whom almost 720,000 were male and over 780,000 female. See Additional file 6, Figures 1, 2, 3.

### Transition probabilities and paths of treatment

For modelling purposes, we calculated the transition probabilities between the Markov states in each of the 28 age- and BP-stratified subgroups for men and women. The transition probabilities between BP groups are based on treatment effects (see Additional file 1, Tables 7 and 8) applied to the individual H2000 data on blood pressure group membership. The estimated probabilities of moving from the various BP group states to the states of CHD, CVEs, and death from those or other causes (i.e., morbidity and fatal event states) are based on the follow-up of the participants in the Mini-Finland (MF) health examination/interview survey undertaken between 1978–1980 [33]. Hazard functions were estimated by stratifying the sample into two age groups, 40–59 and 60–79 (see Additional file 1, Table 9), due to the relative infrequency of endpoints in quinquennial age groups. After the first Markov cycle, the model also allows movements from the morbidity states to two co-morbidity states: the pertinent hazard function estimates are shown in (see Additional file 1, Table 10). All hazard function estimates are transformed into transition probabilities using the formula P(t) = 1 – exp(-*μ*t) [34]. In the few cases where, due to the small number of observations in a subgroup, the estimates were not congruent with the work of MacMahon *et al*. [35], estimates were smoothed.

**Table 6:** Costs of non-pharmacological treatment-related therapies per year*, rounded to the nearest euro, by gender and BPG**.

**Cost**	**ACCG scenario**		**PCP scenario**	
BP measurement	15		5	
initial diagnostic work-up	38		62	
lifestyle counselling	1^st ^year = 36 2^nd ^year = 24 subsequent years = 17		not applicable	
follow-up BP measurement		**Female**	**Male**
**BPG 0**	3	2	0	
**BPG 1**	10	6	4	
**BPG 2**	27	9	7	
**BPG 3**	57	16	14	

* unless otherwise stated

** where applicable

**Table 7:** Estimates of the antihypertensive effect of monotherapy and combination pharmacological treatment.

	**Blood pressure level**	**1 drug**	**2 drugs**	**3 drugs**
**BPG 0**	SBP < 130 mmHg and DBP < 85 mmHg	5/3	10/6	15/8
**BPG 1**	SBP 130–139 mmHg and/or DBP 85–89 mmHg (but not SBP ≥ 140 or DBP ≥ 90 mmHg)	6/3	12/7	18/10
**BPG 2**	SBP 140–159 mmHg and/or DBP 90–99 mmHg (but not SBP ≥ 160 or DBP ≥ 100 mmHg)	7/4	14/8	21/12
**BPG 3**	SBP ≥ 160 mmHg or DBP ≥ 100 mmHg	8/4	16/9	24/13

The scenarios differ in their treatment paths. Common to both are the use of mono-, dual-, or triple-drug therapy and a path defined as monitoring (i.e., without any BP-modifying treatment). Lifestyle counselling (LSC) is used only in the ACCG scenario, with or without drug therapy.

The ACCG scenario follows the treatment options presented in Table 3, such that the type of treatment largely is determined by BPG. In BPG 0 and BPG 1, monitoring and LSC are the only options, respectively. In BPG2, all individuals are assumed to receive LSC and pharmacological therapy can be considered if the individual's risk of CHD within the next 10 years is at least 20% [25]. In BPG 3, all individuals are assumed to receive both LSC and pharmacological therapy.

The choice of initial pharmacological therapy in the ACCG scenario is limited to five alternative monotherapies: a thiazide diuretic, a calcium channel blocker, a beta blocker, an ACE inhibitor, or an angiotensin II subtype 1 receptor antagonist. The latter is recommended by the ACCG when the other drug therapies have resulted in problematic side effects. In the ACCG scenario, at the start of the first five-year Markov cycle, there is a possibility of regimen switching if an individual's hypertension is poorly controlled or there are side effects. According to the ACCG, initial therapy is then changed either to another monotherapy (preferably with a different pharmacological effect) or to a combination treatment (especially if the first drug is a thiazide diuretic or an ACE inhibitor). If blood pressure still remains poorly controlled, the ACCG suggests changing one drug in the two-drug combination, or that adding a third drug with a different effect to the combination should be considered. The possible alternatives for regimen switching in the ACCG scenario are shown in Table 8.

**Table 8:** Possible regimen changes in the ACCG scenario.

Initial drug	1^st ^additional drug	2^nd ^additional drug
thiazide diuretic	ACE inhibitor (or angiotensin II subtype 1 receptor antagonist)	calcium channel blocker or beta blocker
thiazide diuretic	beta blocker	ACE inhibitor (or angiotensin II subtype 1 receptor antagonist) or calcium channel blocker
calcium channel blocker (dihydropyridine derivatives)	ACE inhibitor (or angiotensin II subtype 1 receptor antagonist)	thiazide diuretic or beta blocker
calcium channel blocker	beta blocker	ACE inhibitor (or angiotensin II subtype 1 receptor antagonist) or thiazide diuretic
ACE inhibitor (or angiotensin II subtype 1 receptor antagonist)	thiazide diuretic	calcium channel blocker or beta blocker
ACE inhibitor (or angiotensin II subtype 1 receptor antagonist)	calcium channel blocker	thiazide diuretic or beta blocker
beta blocker	thiazide diuretic	ACE inhibitor (or angiotensin II subtype 1 receptor antagonist) or calcium channel blocker
beta blocker	calcium channel blocker	ACE inhibitor (or angiotensin II subtype 1 receptor antagonist) or thiazide diuretic

The ACCG recommends consideration of costs in the prescription of pharmacological therapies. According to the ACCG the rational first-line treatment would be thiazides for 60% of the population (not complicated by other cardiovascular-related disease) for which drug treatment would be recommended. In part, this recommendation was implemented in the base case analysis of our model by assuming that the majority (60%) of patients receive thiazide diuretics as their initial treatment. In addition, from each pharmacological subgroup a relatively inexpensive and widely used pharmacological substance was chosen.

In the PCP scenario there are 24 possible choices of pharmaceutical monotherapy or pharmaceutical combination therapies, with no possibility of regimen switching. For further details on the interventions available in both scenarios, see Additional file 1, Table 3.

### Estimates of costs

The data used to estimate costs were collated from national registers, Finnish costing studies [36], an earlier national study on the costs of antihypertensive care [37], and an international study of the costs of morbidity associated with elevated blood pressure [38].

For the PCP scenario, information on the shares of use of the pharmacological subgroups of antihypertensive drugs and their combinations (see Additional file 1, Table 4), as well as their average costs in 2001, was obtained from the reimbursement registers of the Finnish Social Insurance Institution (SII). The SII data include all reimbursements made under the National Health Insurance Scheme [39]. In calculation of these costs, patients with entitlement to a special refund on account of concomitant conditions of diabetes and cardiovascular diseases were excluded.

The costs of pharmacological therapies in the ACCG scenario were estimated on the basis of the ACCG recommendation that from each pharmacological subgroup an inexpensive and widely used substance should be chosen. As for the PCP scenario, information on the average costs of antihypertensive drugs was obtained from the reimbursement registers of the SII.

In the base case the substances were valued at their cost to the health care sector in 2001, excluding value added tax (VAT). The estimates of yearly costs (including VAT) are shown in Table 4 and Table 5.

Base case estimates of the non-pharmacological treatment-related costs of prevention, diagnosis, and treatment of hypertension in the ACCG and PCP scenarios are presented in Table 6. Cost estimates were derived by using H2000 data; a Finnish lifestyle counselling study [40]; Finnish health care unit costs [36]; and, where necessary, expert opinion. For further details, see Additional file 4.

Costs applied to the states of CHD, cerebrovascular events, and combinations of these morbid states were estimated from the related literature [38,41-45] and Finnish health care unit costs [36]. The estimate used for CVEs is 3,000 euros per year and for CHD 1,000 euros per year [36,38,41-45]. In the combination states of CVE and CHD, these costs were summed together.

### Estimates of effects

The estimated effect of treatment on outcomes is calculated by first estimating the effect of the expected reduction in BP on BP group and then by the estimating the effect of BP group on morbidity and mortality. These estimated effects are then expressed as changes in life-years, which are calculated on the basis of the cohort's duration of stay in non-fatal states. Life-years are valued equally in all BP and morbidity states in the base case analysis and adjusted for health-related quality of life (HRQL) in a sensitivity analysis. In both the H2000 survey and the MF survey, the measurement of BP was strictly carried out according to the World Health Organization (WHO) recommendations. Antihypertensive effects of treatments are divided into effects associated with medication, those associated with lifestyle counselling and those associated with a combination of the two treatments (see Additional file 1, Tables 7 and 8). The effectiveness of all pharmacological therapies (i.e., the change resulting from any particular pharmacological intervention in terms of change in BP) is assumed to be the same in the ACCG and PCP scenarios.

On account of the work of Kastarinen *et al*. [40], for those receiving both lifestyle counselling (LSC) and pharmacological therapy, a reduction due to LSC of 1 mmHg in both SBP and DBP was assumed in BPG 0, and 2 mmHg in the other BP groups. In the absence of larger RCTs, LSC is assumed to decrease SBP by 2.6 mmHg and DBP by 2.7 mmHg for persons not receiving pharmacological treatment [46].

The effect of five-year increases in age on BP as estimated with regression analysis from the H2000 sample – i.e., the effect of monitoring (no active treatment but active surveillance) – was similar to that obtained in another study [47]. These estimates were calculated for two age groups, 40–60 years old and 60–80 years old. In the first of these groups, SBP was estimated to have increased by 4 mmHg and DBP by 2 mmHg per five-year period, while in the 60–80-year-old group SBP was estimated to have increased by 4 mmHg and DBP decreased by 1 mmHg per five-year period.

The estimates of the antihypertensive effect of pharmacological monotherapies and combination therapies were derived from a recent meta-analysis of randomised trials [48]. According to that study, the five main categories of blood-pressure-lowering drugs (thiazides, beta blockers, ACE inhibitors, angiotensin II subtype 1 receptor antagonists, and calcium channel blockers) produced similar reductions in BP. The expected reductions in BP in different BP groups under both the ACCG and the PCP scenarios are summarised in Table 7.

It is assumed that the effect of treatments within each scenario on BP group membership occurs only during the first five-year cycle. In subsequent cycles, transitions between BP groups result from changes in BP with age under the assumption that only monitoring would occur (for estimates of the resource use in terms of monitoring see Additional file 4, Tables 4, 5 and 6). The Mini-Finland data [30] were used to help provide estimates of the morbidity and mortality associated with BP group. The MF data were linked using exact matching of unique identifiers to the Cause of Death Register (Statistics Finland) and the Hospital Care Register (National Research and Development Centre for Welfare and Health). Cox models [32] were used to produce estimates of hazard functions relating the BP groups to the morbidity and fatal event states for the follow-up period of 15 years (see Additional file 1, Table 9) and estimates of hazard functions relating prior morbidity to future comorbidity and death (see Additional file 1, Table 10). For examples of transition probabilities see Additional file 7.

## Results

In line with recommendations in the literature (see, e.g., [49]), we report analyses in both subgroup and aggregated form. For the 56 age- and gender-stratified subgroups, the estimated ICERs and the corresponding numerators and denominators for the base case analysis are shown (Figure 1). Green cells in Figure 1 (21 in number) show dominance of the ACCG scenario; that is, application of the ACCG scenario is more effective (produces more life-years) and is less costly than the PCP scenario. Yellow shading (12 cells) indicates incremental costs and incremental outcomes, while orange shading (22 cells) indicates decremental costs and decremental outcomes. That is, the smaller the ICERs with yellow shading or, conversely, the larger the ICERs with orange shading, the more likely the ICERs are to be considered cost-effective. Red shading (one cell) represents incremental costs and decremental outcomes (i.e., the PCP scenario dominates). These results are given for each subgroup over the time horizon of the study (10 to 40 years, depending on the age range of the subgroup). See Additional file 1, Table 11 for the estimated sizes of subgroups. The total target population is almost 1.5 million individuals in Finland.

In large part, the subgroup results differ according to blood pressure group (BPG). Generally, in blood pressure group 0 (BPG 0, where SBP is below 130 mmHg and DBP is below 85 mmHg) the effect of the application of the ACCG scenario was to reduce expected costs at the same time as reducing the expected number of life-years. Largely, for BPG 1 and BPG 2 (where SBP is 130–139 mmHg and/or DBP 85–89 mmHg and SBP is 140–159 mmHg and/or DBP 90–99 mmHg, respectively), the ACCG scenario was shown to be cost-saving and more effective (i.e., decreased costs and increased life-years expected). Generally, in BPG 3 (where SBP is over 160 mmHg or DBP over 100 mmHg), increased expected costs are associated with an increase in the expected number of life-years. Figure 3 shows these subgroup results plotted on the cost-effectiveness plane [50]. We also provide the approximate size of the Finnish population to which the results in that quadrant apply (the numbers in brackets in Figure 3).

As a summary of both Figure 1 and Figure 3, aggregating the results from the 56 study subgroups indicates that in comparison to the PCP scenario the use of the ACCG scenario would produce 49,000 extra life-years and save 498 million euros. In this case, the ACCG would be the dominant scenario overall. That is, while the ACCG scenario should not be classed as cost-effective, it is both cost-saving and more effective [51].

**Figure 4:**
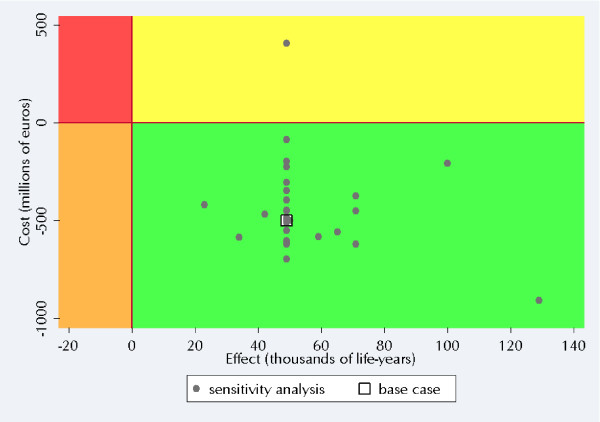
The cost-effectiveness plane with the results (aggregated across subgroups) of the one-way sensitivity analyses.

A cost-effectiveness plane based on the results of one-way sensitivity analyses (aggregated over all subgroups) of 30 variations of influential variables is shown in Figure 4. For a detailed presentation of the results of these sensitivity analyses, see Additional file 5. Almost all aggregated sensitivity analyses showed that the ACCG scenario dominates the PCP scenario – i.e., that the ACCG scenario is cost-saving and more effective. One exception was the case where lifestyle counselling was assumed to be four times more costly than in the base case. In this sensitivity analysis, with an extreme value used for the cost of lifestyle counselling, the aggregated results show that the cost per life-year saved would be around €8,000.

Despite many thousands of life-years being saved in all aggregated subgroup analyses, the overall benefit per person is modest. Applying the ACCG scenario would add 12 (six at least, 31 at best) days per person in the target population, although it should be noted that this figure is averaged over all individuals in the target population of 1.5 million Finns.

## Discussion

Over half of Finland's 40–74-year-old 'uncomplicated' population has elevated blood pressure. The major goal of the ACCG is to achieve a reduction in blood pressure that is sufficient for these individuals to lower their risk of cardiovascular (and related) diseases. As a means to this end, the ACCG promotes lifestyle modification and rational use of antihypertensive drugs. The lifestyle counselling considered to be feasible in the ACCG scenario is by design a low-intensity patient-counselling programme [40]. The most significant lifestyle-related and modifiable risk factors for elevated blood pressure are overweight status, high intake of sodium, high intake of alcohol, and physical inactivity. Also, a randomised controlled trial has shown the beneficial effects of a lifestyle intervention based on reduced sodium intake and increased intake of fruits, vegetables, and low-fat dairy products [52]. Even more dramatic effects could be achieved with a more aggressive lifestyle intervention [53,54] than is considered here.

Generally, in blood pressure group 0, the impact of the application of the ACCG scenario was to reduce expected costs at the same time as reducing the expected number of life-years in comparison to the PCP scenario. This result is likely to reflect the fact that the PCP scenario, with at least some use of medication, is always treated by the model as being more effective than monitoring (i.e., in BPG 0 active surveillance is the only treatment option under the ACCG scenario). In the majority of age- and gender-stratified subgroups in BPG 1 and BPG 2, the ACCG scenario was shown to be both cost-saving and more effective. Indeed, these two groups are those for which, a priori, we expected to see the greatest relative benefit from lifestyle counselling, either alone (in the case of BPG 1) or in combination with medication for individuals at increased risk of CHD (in the case of BPG 2). Generally, in BPG 3, increased expected costs are associated with an increase in the expected number of life-years. This mainly reflects the fact that the ACCG scenario applies drug treatment to all individuals in BPG 3, whereas the PCP scenario treats only some individuals in this group.

Other factors that are likely to have contributed greatly to the results presented include both the manner in which diagnosis is performed in the ACCG scenario and the fact that inexpensive medications from within each pharmacological subgroup are used under the ACCG scenario. The rational use of antihypertensive drugs can be promoted by reference to both the severity of hypertension and the costs of drug treatment [23,55]. The ACCG, for instance, promotes the initiation of drug treatment using a single pharmacological therapy [23]. In addition, for most instances of combination treatment, one of the drugs to be chosen is a thiazide diuretic [56].

### Strengths of the study

This analysis is intended to be a pragmatic cost-effectiveness analysis and firmly based on observed prior clinical practice in Finland and on the operationalisation of the Finnish evidence-based guidelines undertaken in this study. Generalising the results of this study may, to some extent, be justified in view of the broadly similar hypertension-related disease burden in other settings and the similarity of other evidence-based antihypertensive guidelines, such as the recently updated National Institute for Clinical Excellence (NICE) guidelines [57].

The study presented here is based on an extraordinary set of data sources, which include both exemplary national registers with high coverage [58] and high-quality surveys of population health (see [31] and [33]). The Hospital Care Register, particularly as regards cardiovascular diseases, has proved to be accurate [59]. The National Health Insurance Scheme operates in all Finnish pharmacies, and over 90% of reimbursements for the purchase of antihypertensive drugs occur seamlessly at the point of sale.

In both the H2000 and MF surveys, the measurement of BP was carried out according to the WHO recommendations. It is therefore credible that these datasets provide classification of BP in line with measurements made in standard clinical practice. In addition, both SBP and DBP are used as part of the basis for BP classification, as recommended by the ACCG.

Perhaps most importantly, this study offers rare insight into the potential usefulness of developing clinical practice guidelines.

### Limitations of the study

A number of caveats should be attached to all results and discussion presented here, the following two being of major importance. Firstly, that this is a model of limited size, analytic capability, and adherence to economic evaluation guidelines. Secondly, because uncertainty concerning the model inputs has not been fully incorporated, the cost-effectiveness analyses reported here are best taken as being indicative of the direction, rather than the exact magnitude, of differences in costs and effects between the two scenarios.

Further to this, pertinent evidence of adherence to either the ACCG scenario or the PCP scenario had not been published. The full-adherence assumption used in this study deviates, somewhat, from what might be expected in standard clinical practice. However, the practical effect of this assumption is likely to be reduced by the fact that it was used in the same manner in both scenarios. Indeed, the financial costs associated with guideline development and production in Finland are estimated to be minimal, and, even with very low levels of adherence, their production is likely to be economically viable in the long term. The results of a sensitivity analysis concerning low levels of adherence to the ACCG scenario in favour of continued use of the PCP scenario are not presented here, as the result is merely a linear scaling of the differences in the costs and effects between the two scenarios. However, the possibility remains that these results may be located toward the upper end of the potential overall impact of the ACCG scenario.

The ACCG itself is an evidence-based, advisory statement, and treatment should always be tailored to the individual. On the other hand, in the Markov model used here, the ACCG scenario is implemented as if it were a prescriptive scenario. Thus, the model does not fully incorporate the subtleties and flexibilities of the ACCG. The result that the ACCG scenario, in almost all age- and gender-stratified BPG 0 subgroups, produces fewer life-years and costs than the PCP scenario is also explained in part by the rigidity of the model. In contrast to the observed use of antihypertensive medications in prior clinical practice, in the ACCG scenario no intervention is provided for BPG 0 (see Table 3). It is likely that the H2000 records on prior usage of antihypertensive medications include some element of overtreatment.

This study did not directly consider the cost of ACCG development, nor the costs of ACCG implementation in clinical practice [60]. This leads to an underestimate of the costs of the ACCG scenario, but can be justified by reference to the fact that the ACCG scenario is not identically equivalent to the whole of the evidence-based ACCG. The costs included can be considered to consist mainly of the costs borne by the health care sector. Value added tax was subtracted from the recorded prices of the pharmaceuticals. This could result in an underestimate of the cost burden to the health care sector [61].

The number of treatments considered and the number of health states representing alternative outcomes had to be restricted in order to keep the Markov model simple enough to be functional and able to populate the model. Carrying out the analyses in 56 subgroups led to moderately small sample sizes in some cases, but this was deemed necessary for identifying any heterogeneity of the impact on the subgroups. For example, due to lack of reliable or consistent data, this study does not incorporate some health care costs and outcomes that potentially could be associated with elevated blood pressure, such as the costs and health-related effects of peripheral vascular disease, renal disease, heart failure, diabetes, and lost earnings [62]. This may well have resulted in an underestimate of the costs and effects associated with BP-related disease but is also likely to result in an underestimate of potential savings and benefits in outcome from BP reduction at the aggregated level.

At best, the estimates of BP effects on mortality and morbidity should be treated as only indicative of the extent and direction of the likely associations between BPG and health status in terms of morbidity and mortality. The estimates presented here are also subject to the assumption of full benefit [12]. This assumes that the estimated change in BP is achieved, that the effect of non-adherence is negligible, that the change to (or inclusion in) a BP group completely and linearly defines the risk of morbidity and mortality, and that the amount of benefit does not diminish within any age group. For a detailed presentation of other assumptions applied in this research, see Additional file 8.

The estimates of the blood-pressure-reducing effects of antihypertensive drugs and their combinations are to some extent uncertain. At the time this CEA was undertaken, data concerning the antihypertensive effect of ACE inhibitors and beta-blockers in combination could not be located. Combination treatment involving angiotensin II subtype 1 receptor antagonists had been studied only for combinations involving diuretics. Research on three-drug combination treatment is especially sparse [57]. Drugs with different pharmacological mechanisms – i.e., different classes of antihypertensive drugs – seem to intensify one another's effects.

The potential effect of substitution with generic equivalents, for which legislation has been in place in Finland since 1 April 2003, was not incorporated into the base case analysis. Equivalence, in terms of side effects, of the pharmacological therapies was assumed in the absence of strong evidence to the contrary. Therefore, the impact of potential side effects on antihypertensive care (discontinuation of treatment or regimen change and costs associated therewith) or on individuals (HRQL effects) is assumed to be the same and is omitted from consideration.

Probabilistic sensitivity analysis was not carried out, partly on account of the assumed robust nature of the datasets used [63]. In addition, the complexity of the Markov model did not allow easy application of probabilistic sensitivity analysis. One-way sensitivity analyses showed that exaggerated increases in the cost of lifestyle counselling was the only variant investigated that changed the aggregated result from that of the ACCG scenario being dominant to that of it being both more costly and more effective.

## Conclusion

The aggregated results showed that the ACCG scenario is less costly and produces more life-years than the PCP scenario. However, there was heterogeneity in the results from the 56 subgroups analysed – i.e., ranging from losses in life-years and increased costs in one subgroup to gains in life-years and reduced costs in others. The most consistently positive effects of the ACCG scenario (decreased costs and an increased number of life-years) were observed for males with moderately elevated blood pressure – that is, for those in BPGs 1 and 2. On the other hand, generally, the effect of the application of the ACCG scenario in BPG 0 was to reduce costs at the same time as the number of life-years.

Although aggregated results alone can be of value to decision-making entities, here they are accompanied by more detailed information from subgroup-specific results. This subgroup-specific information is of potential importance to decision-making entities, too. The aggregated ICER results presented here assume that individuals and groups are treated equally in keeping with *ex ante *equity concerns – i.e., that, as is usual in cost-effectiveness analyses, equity is restricted to a specific form of 'equitable efficiency' [64]. 'Equitably efficient' usually refers to a situation where reductions in life-years for one group are given an equal and opposite weight to gains in life-years in another. For example, here we assume that the relevant objective function is the maximisation of a proxy for health – life-years – and that each life-year is valued equally, irrespective of which group loses or gains it. However, generally, society-level decision-makers' objectives include separate considerations of efficiency and equity. Therefore, whether the ACCG scenario represents an improvement in societal welfare and thus is preferable to the PCP scenario is a value judgement [65], and the applicability of the consideration of equity employed here is left for decision-makers to judge.

If the estimated trade-offs between the subgroups in terms of outcomes and costs are acceptable to decision-makers, then widespread implementation of the ACCG scenario is expected to reduce overall costs and be accompanied by positive outcomes overall.

## Abbreviations

ACCG = the 2002 Antihypertensive Current Care Guideline [23]

ACE = angiotensin-converting enzyme

ATC = Anatomical Therapeutic Chemical classification system

BP = blood pressure (indirect measurement using an external measuring device)

BPG = blood pressure group (see Table 2)

C03EA = hydrochlorothiazide or trichlormethiazide and potassium-sparing agents

C07A = beta-blocking agents

C07B = combination of metoprolol or bisoprolol and thiazides

C07F = atenolol or metoprolol and, e.g., calcium channel blockers

C08 = calcium channel blockers

C09A = ACE inhibitors

C09BA = combination of ACE inhibitors and diuretics

C09BB = combination of ACE inhibitors and calcium channel blockers

C09C = angiotensin II subtype 1 receptor antagonists

C09D = combination of angiotensin II subtype 1 receptor antagonists and diuretics

CEA = cost-effectiveness analysis (see Additional file 1, Table 2 for further details)

CHD = coronary heart disease

CVE = cerebrovascular event

DBP = diastolic blood pressure

H2000 = Health 2000 health examination/interview survey (2000–2001)

HRQL = health-related quality of life

ICER = incremental cost-effectiveness ratio (see Additional file 1, Table 2 for further details)

LSC = lifestyle counselling (only used in the ACCG scenario)

MF = Mini-Finland health examination/interview survey (1978–1980)

PCP = prior clinical practice (clinical practice prior to the publication of the ACCG)

SBP = systolic blood pressure

SII = Finnish Social Insurance Institution (KELA)

## Competing interests

Antti Jula declares: 'I was the chair of the task force which developed the evidence-based antihypertensive guidelines [23] and continue to be involved in the updating of these guidelines.'

Minna Kaila declares: 'I was the chief editor of *Current Care Guidelines *at the time this economic evaluation was initiated. I am still an editor, though on a strictly part-time basis (one day per month), and I am not directly involved with guideline development.'

All other authors declare that they have no competing interests.

## Authors' contributions

MM, AJ, PR, KK-H, MK, TK, HS, and NB designed the study; NB, AJ, AR, and TK collected the data; NB, PA, and AR conducted the data analysis; NB, AJ, HS, PA, AR, MK, and MM drafted the paper; and HS, KK-H, MM, MK, AR, PA, and PR critically revised the paper. All authors have read and approved the final version of the manuscript.

## Pre-publication history

The pre-publication history for this paper can be accessed here:



## Supplementary Material

Additional File 1 Extra Tables. Supplementary information in tablular form. Click here for file

Additional File 2 Current Care guidelines. Supplementary information describing the Current Care guideline process. Click here for file

Additional File 3 Extra Figures. Supplementary figures. Click here for file

Additional File 4 Details of cost calculations. Detailed calculations of the non-pharmacological treatment-related costs for the ACCG and PCP scenarios. Click here for file

Additional File 5 Sensitivity analysis. Full results of the sensitivity analysis undertaken on subgroups. Click here for file

Additional File 6 Population sizes. Supplementary details concerning the size of the study and target populations. Click here for file

Additional File 7 Transition probabilities. Supplementary details concerning transition probabilities. Click here for file

Additional File 8 Main assumptions in this study. Supplementary details of the main assumptions used in this study. Click here for file
