# Supplementary material for: Cost-effectiveness analysis of guidelines for antihypertensive care in Finland
Source: BMC Health Serv Res. 2007 Oct 24;7:172. doi: 10.1186/1472-6963-7-172 (PMC2174470; doi:10.1186/1472-6963-7-172)
Supplement: Additional File 1 — Extra Tables. Supplementary information in tablular form. [file 1472-6963-7-172-S1.pdf]

Table 1: Literature search strategy.

Search: "Cost-effectiveness of evidence-based guidelines"
Database: Ovid MEDLINE(R) <1996 to March Week 4 2005>
Date of search: 05/04/05

		**number of hits**
1	Health Planning Guidelines/	(686)
2	exp practice guidelines/	(24437)
3	guideline.pt.	(8359)
4	guideline$.ti,ab.	(44083)
5	exp consensus development conferences/ or exp consensus/ or exp consensus development conferences, nih/	(1538)
6	(consensus development conference or NIH consensus development conference).pt.	(2893)
7	consensus.ti,ab.	(28257)
8	Clinical Medicine/ec, st [Economics, Standards]	(283)
9	1 or 2 or 3 or 4 or 5 or 6 or 7 or 8	(89213)
10	(recommend$ or protocol$ or strateg$).mp. [mp=title, original title, abstract, name of substance word, subject heading word]	(300092)
11	(care or treatment? or therap$ or manag$).mp. [mp=title, original title, abstract, name of substance word, subject heading word]	(1354275)
12	((guideline$ adj2 care) or (guideline$ adj2 therap$) or (guideline$ adj2 manag$)).mp	(5710)
13	10 and 11	(176619)
14	9 or 12	(249624)
15	exp Evidence-Based Medicine/	(16196)
16	eviden$.ti,ab.	(309317)
17	15 or 16	(316328)
18	(cost$ or econom$ or fee? or charg$ or price? or pricing).mp. [mp=title, original title, abstract, name of substance word, subject heading word] (201390)	
19	exp "costs and cost analysis"/ or exp economics, medical/ or "fees and charges"/ or financial management/ or financial support/	(59646)
20	18 or 19	(204995)
21	13 and 17 and 20	(3861)
22	9 and 17 and 189	(626)
23	EVIDENCE-BASED MEDICINE/ or evidence$.ti.	(48725)
24	exp *"Costs and Cost Analysis"/ or cost$.ti.	(26792)
25	*HEALTH PLANNING GUIDELINES/ or *GUIDELINES/ or *PRACTICE GUIDELINES/ or guideline$.ti.	(19067)
26	14 and 24	(81
27	22 and 23 and 24	(23)
28	26 or 27	(98)

Similar searches strategies were undertaken using the following databases: the Health Technology Assessment Database (HTA); Database of Abstracts of Reviews of Effects (DARE); NHS Economic Evaluation Database (NHS EED); GIN Library; EBM Reviews – Cochrane Central Register of Controlled Trials <1st Quarter 2005>; and Cochrane Methodology Register.

Table 2: Brief glossary of some main terms and concepts used in this study.

cost	given the *perspective* of this study, the costs included in the base case are restricted to some of the major monetary costs borne by the health care sector or public health care system.
CEA (cost-effectiveness analysis)	‘a method of comparing the *opportunity costs* of various alternative courses of action having the same benefit or in terms of a common unit of output, outcome or other measure of accomplishment’, according to [1] (italics added). To paraphrase Sugden & Williams [2], the problem of cost-effectiveness analysis is to attempt to maximise economic efficiency subject to the constraint that one of the alternatives is chosen.
decision analytic modelling	a method of synthesising information concerning uncertain choice(s) between alternatives and assessing the expected effects of the choices considered on costs and outcomes [3]. Here we use a *Markov model* approach.
cost per LY	given the public health care system *perspective* of this study, the average expected *cost* of each unit of outcome (i.e., that per *LY*).
ICER (incremental cost-effectiveness ratio)	a ratio usually expressed as the *cost* per unit change in (health-related) *outcome* obtained through a specific (health) intervention compared with a well-defined alternative intervention. In our base case analysis, the *ICER*s presented are of the form *cost per LY*. Comparative changes in life-years, changes in costs and *ICER*s show the *opportunity costs* both for subgroups and overall, according to the *decision analytic modelling* approach used here.
LY (life-year)	the expected value – i.e., estimated *outcome* – of each scenario in terms of estimated ‘years of life’ in the target population over the period of analysis.
Markov model	a type of *decision analytic modelling* that characterises the prognosis of a cohort of individuals by assigning them to a fixed number of health states and modelling transitions among those states [4].
opportunity cost	the context-specific value, in terms of what is forgone, of using resources elsewhere instead of in their current use. To quote [5]: ‘in common sense terms, the [opportunity] cost of any service is what you sacrifice in order to obtain it.’
outcomes	the health-related consequences associated with antihypertensive care. Given the *perspective* of this study, our base case analysis focuses on key forms of mortality and morbidity, in terms of *LY*s, but does not include all potential impacts on society.
perspective	the perspective of this study can be described as a ‘public health care system’ perspective [6]. The perspective of *costs* and *outcomes* is, broadly, that of the publicly funded health care sector, and the perspective of the aggregation process is that of *decision analytic modelling*.

Table 3: Interventions available in the PCP and ACCG scenarios.

PCP	ACCG
monitoring	monitoring
C03EA	lifestyle counselling
C07A	C03 & LSC
C07B	C03+C09A & LSC
C07F	C03+C09C & LSC
C08	C07 & LSC
C09A	C07+C03 & LSC
C09BA	C07+C08 & LSC
C09C	C07+C03+C08 & LSC
C09D	C08 & LSC
C03EA+C07A	C08+C09A & LSC
C03EA+C08	C08+C09C & LSC
C03EA+C09A	C09A & LSC
C07A+C08	C09C & LSC
C07A+C09A	C09A+C03+C07 & LSC
C07A+C09BA	C09A+C03+C08 & LSC
C07A+C09C	C09A+C07+C08 & LSC
C07A+C09D	C09C+C03+C07 & LSC
C08+C09A	C09C+C03+C08 & LSC
C08+C09BA	C09C+C07+C08 & LSC
C08+C09C	
C08+C09D	
C03EA+C07A+C08	
C07A+C08+C09A	
C07A+C08+C09BA*	

* Therapy using C07A, C08, and C09BA includes four classes of antihypertensive drugs. In this case, the effects are assumed to be equivalent to those of triple-drug therapy. In 2001 in Finland, therapy using C07A, C08, and C09BA together was used by less than 1% of men and less than 2% of women (according to SII records).

Abbreviations used:

C03A = hydrochlorothiazide
C03EA = hydrochlorothiazide or trichlormethiazide and potassium-sparing agents
C07A = beta-blocking agents
C07B = combination of metoprolol or bisoprolol and thiazides
C07F = atenolol or metoprolol and other antihypertensives
C08 = calcium channel blockers
C09A = ACE inhibitors
C09BA = combination of ACE inhibitors and diuretics
C09BB = combination of ACE inhibitors and calcium channel blockers
C09C = angiotensin II subtype 1 receptor antagonists
C09D = combination of angiotensin II subtype 1 receptor antagonists and diuretics
LSC = lifestyle counselling (the ACCG lifestyle intervention)

Table 4: Percentage of antihypertensive pharmacological interventions reimbursed by the SII under PCP*, by gender and age (N = 254,877).

September–November 2001																
	**females, age**								**males, age**							
**ATC codes and combinations**	**40–44**	**45–49**	**50–54**	**55–59**	**60–64**	**65–69**	**70–74**	**All** (40–74)	**40–44**	**45–49**	**50–54**	**55–59**	**60–64**	**65–69**	**70–74**	**All** (40–74)
**C03EA**	1.3	1.4	2.1	2.4	3.3	4.2	5.2	4.2	0.2	0.2	0.5	0.5	0.8	1.3	2.1	1.2
**C07A**	22.1	17.4	15.0	14.4	14.6	14.0	12.8	14.0	13.6	13.6	13.2	13.0	13.5	13.7	14.0	13.5
**C07B**	2.8	3.1	2.9	2.3	2.0	1.8	1.6	1.9	1.9	1.8	1.4	1.1	1.1	0.9	1.0	1.2
**C07F**	2.7	2.7	2.7	2.5	2.2	2.1	1.9	2.0	3.6	3.5	3.6	3.3	3.1	2.8	2.2	2.9
**C08**	6.2	5.8	6.6	7.4	9.0	9.6	9.3	8.4	6.0	7.1	7.8	8.9	10.5	11.1	12.4	9.8
**C09A**	13.6	12.2	10.4	9.6	8.8	7.3	6.1	8.0	15.3	14.2	13.3	11.9	11.5	10.8	9.6	11.6
**C09BA**	7.2	8.1	8.3	7.8	7.2	5.3	4.6	5.8	7.9	8.5	8.6	7.8	7.0	6.4	5.3	6.9
**C09C**	6.1	5.5	4.7	3.9	3.1	2.5	2.3	3.1	6.1	5.7	4.4	3.9	3.0	2.7	2.1	3.5
**C09D**	4.7	4.6	4.1	3.6	3.1	2.3	2.0	2.7	4.7	4.3	3.5	3.1	2.6	1.6	1.7	2.7
**C03EA+C07A**	3.6	4.2	5.3	6.2	7.1	8.2	8.6	7.1	1.0	1.3	1.6	2.0	2.4	3.4	4.4	2.8
**C03EA+C08**	0.8	1.0	1.4	1.6	2.2	2.4	2.8	2.3	0.5	0.6	0.6	0.8	1.1	1.2	1.5	1.1
**C03EA+C09A**	0.7	1.1	1.4	1.6	1.7	1.5	1.5	1.4	0.4	0.5	0.6	0.7	0.9	1.1	1.0	0.8
**C07A+C08**	4.4	4.9	4.6	5.3	5.3	5.9	6.1	5.4	5.3	6.1	6.9	7.1	7.7	7.9	7.6	7.2
**C07A+C09A**	2.9	3.4	3.0	3.0	3.1	3.2	3.0	2.9	4.4	4.0	4.4	4.7	4.4	4.5	4.2	4.3
**C07A+C09BA**	2.0	2.3	2.8	3.1	2.7	2.4	2.5	2.4	3.3	2.9	3.5	3.4	3.5	2.8	2.7	3.0
**C07A+C09C**	1.5	1.6	1.6	1.5	1.3	1.3	1.2	1.3	1.7	1.3	1.5	1.4	1.3	1.2	1.0	1.2
**C07A+C09D**	1.8	1.6	1.7	1.8	1.5	1.4	1.4	1.4	1.8	1.5	1.3	1.3	1.0	1.0	0.9	1.1
**C08+C09A**	1.1	1.4	1.2	1.3	1.4	1.5	1.4	1.4	2.5	2.3	2.6	2.8	3.1	3.2	2.6	2.7
**C08+C09BA**	1.0	1.2	1.4	1.3	1.3	1.3	1.1	1.2	2.6	2.5	2.7	2.9	2.4	2.3	2.0	2.4
**C08+C09C**	0.5	0.5	0.8	0.7	0.6	0.7	0.7	0.6	1.1	1.2	1.1	1.0	1.0	0.8	0.6	0.9
**C08+C09D**	0.6	0.7	0.9	0.7	0.8	0.6	0.6	0.6	1.6	1.3	1.2	1.2	0.9	0.7	0.6	0.9
**C03EA+C07A+C08**	0.3	0.7	1.0	1.0	1.3	1.5	1.5	1.3	0.1	0.2	0.4	0.4	0.4	0.7	0.5	0.5
**C07A+C08+C09A**	0.3	0.3	0.4	0.5	0.5	0.7	0.7	0.6	0.8	1.2	1.3	1.3	1.4	1.5	1.5	1.3
**C07A+C08+C09BA**	0.3	0.5	0.7	0.7	0.7	0.8	0.9	0.7	0.9	1.4	1.5	1.7	1.2	1.3	1.3	1.3
Overall coverage of the 24 most used drugs	**88.61**	**86.29**	**85.21**	**84.41**	**84.63**	**82.43**	**79.99**	**80.87**	**87.29**	**87.40**	**87.45**	**86.19**	**85.75**	**85.09**	**82.90**	**84.77**

* excluding patients with entitlement to a special refund on account of certain concomitant conditions

Table 5: Matrix of state transitions.

From \ To	BPG 0	BPG 1	BPG 2	BPG 3	CHD	CVE	CHD death	CVE death	Other death
BPG 0	**H2000TP**	**H2000TP**	**H2000TP**	**H2000TP**	**MFTP**	**MFTP**	**MFTP**	**MFTP**	**MFTP**
BPG 1	**H2000TP**	**H2000TP**	**H2000TP**	**H2000TP**	**MFTP**	**MFTP**	**MFTP**	**MFTP**	**MFTP**
BPG 2	**H2000TP**	**H2000TP**	**H2000TP**	**H2000TP**	**MFTP**	**MFTP**	**MFTP**	**MFTP**	**MFTP**
BPG 3	**H2000TP**	**H2000TP**	**H2000TP**	**H2000TP**	**MFTP**	**MFTP**	**MFTP**	**MFTP**	**MFTP**
CHD	**0**	**0**	**0**	**0**	#	**MFTP**	**MFTP**	**MFTP**	**MFTP**
CVE	**0**	**0**	**0**	**0**	**MFTP**	#	**MFTP**	**MFTP**	**MFTP**
CHD death	**0**	**0**	**0**	**0**	**0**	**0**	**1**	**0**	**0**
CVE death	**0**	**0**	**0**	**0**	**0**	**0**	**0**	**1**	**0**
Other death	**0**	**0**	**0**	**0**	**0**	**0**	**0**	**0**	**1**

# = remainder probability.

H2000TP = probabilities calculated as set out in the section '*Transition probabilities and paths of treatment*' in the main text, using H2000 data and expert estimates of effectiveness.

MFTP = probabilities calculated as set out in the section '*Transition probabilities and paths of treatment*' in the main text, using MF data and register data.

Table 6: BP adjustment for prior treatment in the H2000 sample.

	estimated SBP change (mmHg)	estimated DBP change (mmHg)	approximate proportion of population in each BP group	approximate proportion of population using various therapies		estimated SBP change (mmHg)	estimated DBP change (mmHg)
BPG 0	5	3	0.35	0.5	monotherapy	0.875	0.525
BPG 1	6	3	0.2	0.5	monotherapy	0.6	0.3
BPG 2	7	4	0.3	0.5	monotherapy	1.05	0.6
BPG 3	8	4	0.15	0.5	monotherapy	0.6	0.3
						all 3.125	1.725
BPG 0	10	6	0.35	0.35	dual therapy	1.225	0.735
BPG 1	12	7	0.2	0.35	dual therapy	0.84	0.49
BPG 2	14	8	0.3	0.35	dual therapy	1.47	0.84
BPG 3	16	9	0.15	0.35	dual therapy	0.84	0.4725
						all 4.375	2.5375
BPG 0	15	8	0.35	0.15	triple therapy	0.7875	0.42
BPG 1	18	10	0.2	0.15	triple therapy	0.54	0.3
BPG 2	21	12	0.3	0.15	triple therapy	0.945	0.54
BPG 3	24	13	0.15	0.15	triple therapy	0.54	0.2925
						all 2.8125	1.5525
						SBP adjustment	DBP adjustment
						10	6

Table 7: Intervention-induced BP effects (mean changes according to PCP and ACCG), by age group.

Without ageing (male and female)									
Therapy	BP								
	group	**PCP**				**ACCG**			
(**na =** not applicable)		40–60 years old		60–80 years old		40–60 years old		60–80 years old	
		Systolic	Diastolic	Systolic	Diastolic	Systolic	Diastolic	Systolic	Diastolic
Single-drug therapy	0	**-5.0**	**-3.0**	**-5.0**	**-3.0**	**-6.0**	**-4.0**	**-6.0**	**-4.0**
Single-drug therapy	1	**-6.0**	**-3.0**	**-6.0**	**-3.0**	**-8.0**	**-5.0**	**-8.0**	**-5.0**
Single-drug therapy	2	**-7.0**	**-4.0**	**-7.0**	**-4.0**	**-9.0**	**-6.0**	**-9.0**	**-6.0**
Single-drug therapy	3	**-8.0**	**-4.0**	**-8.0**	**-4.0**	**-10.0**	**-6.0**	**-10.0**	**-6.0**
Dual-drug therapy	0	**-10.0**	**-6.0**	**-10.0**	**-6.0**	**-11.0**	**-7.0**	**-11.0**	**-7.0**
Dual-drug therapy	1	**-12.0**	**-7.0**	**-12.0**	**-7.0**	**-14.0**	**-9.0**	**-14.0**	**-9.0**
Dual-drug therapy	2	**-14.0**	**-8.0**	**-14.0**	**-8.0**	**-16.0**	**-10.0**	**-16.0**	**-10.0**
Dual-drug therapy	3	**-16.0**	**-9.0**	**-16.0**	**-9.0**	**-18.0**	**-11.0**	**-18.0**	**-11.0**
Triple-drug therapy	0	**-15.0**	**-8.0**	**-15.0**	**-8.0**	**-16.0**	**-9.0**	**-16.0**	**-9.0**
Triple-drug therapy	1	**-18.0**	**-10.0**	**-18.0**	**-10.0**	**-20.0**	**-12.0**	**-20.0**	**-12.0**
Triple-drug therapy	2	**-21.0**	**-12.0**	**-21.0**	**-12.0**	**-23.0**	**-14.0**	**-23.0**	**-14.0**
Triple-drug therapy	3	**-24.0**	**-13.0**	**-24.0**	**-13.0**	**-26.0**	**-15.0**	**-26.0**	**-15.0**
Lifestyle counselling	0	**na**	**na**	**na**	**na**	**-2.6**	**-2.7**	**-2.6**	**-2.7**
Lifestyle counselling	1	**na**	**na**	**na**	**na**	**-2.6**	**-2.7**	**-2.6**	**-2.7**
Lifestyle counselling	2	**na**	**na**	**na**	**na**	**-2.6**	**-2.7**	**-2.6**	**-2.7**
Lifestyle counselling	3	**na**	**na**	**na**	**na**	**-2.6**	**-2.7**	**-2.6**	**-2.7**

Numbers here are, with the aid of expert opinion, derived from the evidence base set forth in the section ‘*Estimates of effects*’ in the main text.

Table 8: Intervention's effects* on BP over a five-year period (with estimated age-related BP changes).**

With ageing (male and female)									
Therapy	BP								
	group	**PCP**				**ACCG**			
(**na =** not applicable)		40–60 years old		60–80 years old		40–60 years old		60–80 years old	
		Systolic	Diastolic	Systolic	Diastolic	Systolic	Diastolic	Systolic	Diastolic
Single therapy	0	**-1.0**	**-1.0**	**-1.0**	**-4.0**	**-2.0**	**-2.0**	**-2.0**	**-5.0**
Single therapy	1	**-2.0**	**-1.0**	**-2.0**	**-4.0**	**-4.0**	**-3.0**	**-4.0**	**-6.0**
Single therapy	2	**-3.0**	**-2.0**	**-3.0**	**-5.0**	**-5.0**	**-4.0**	**-5.0**	**-7.0**
Single therapy	3	**-4.0**	**-2.0**	**-4.0**	**-5.0**	**-6.0**	**-4.0**	**-6.0**	**-7.0**
Dual therapy	0	**-6.0**	**-4.0**	**-6.0**	**-7.0**	**-7.0**	**-5.0**	**-7.0**	**-8.0**
Dual therapy	1	**-8.0**	**-5.0**	**-8.0**	**-8.0**	**-10.0**	**-7.0**	**-10.0**	**-10.0**
Dual therapy	2	**-10.0**	**-6.0**	**-10.0**	**-9.0**	**-12.0**	**-8.0**	**-12.0**	**-11.0**
Dual therapy	3	**-12.0**	**-7.0**	**-12.0**	**-10.0**	**-14.0**	**-9.0**	**-14.0**	**-12.0**
Triple therapy	0	**-11.0**	**-6.0**	**-11.0**	**-9.0**	**-12.0**	**-7.0**	**-12.0**	**-10.0**
Triple therapy	1	**-14.0**	**-8.0**	**-14.0**	**-11.0**	**-16.0**	**-10.0**	**-16.0**	**-13.0**
Triple therapy	2	**-17.0**	**-10.0**	**-17.0**	**-13.0**	**-19.0**	**-12.0**	**-19.0**	**-15.0**
Triple therapy	3	**-20.0**	**-11.0**	**-20.0**	**-14.0**	**-22.0**	**-13.0**	**-22.0**	**-16.0**
Lifestyle counselling	0	**na**	**na**	**na**	**na**	**1.4**	**-0.7**	**1.4**	**-3.7**
Lifestyle counselling	1	**na**	**na**	**na**	**na**	**1.4**	**-0.7**	**1.4**	**-3.7**
Lifestyle counselling	2	**na**	**na**	**na**	**na**	**1.4**	**-0.7**	**1.4**	**-3.7**
Lifestyle counselling	3	**na**	**na**	**na**	**na**	**1.4**	**-0.7**	**1.4**	**-3.7**
Monitoring group	0	**4.0**	**2.0**	**4.0**	**-1.0**	**4.0**	**2.0**	**4.0**	**-1.0**
Monitoring group	1	**4.0**	**2.0**	**4.0**	**-1.0**	**4.0**	**2.0**	**4.0**	**-1.0**
Monitoring group	2	**4.0**	**2.0**	**4.0**	**-1.0**	**4.0**	**2.0**	**4.0**	**-1.0**
Monitoring group	3	**4.0**	**2.0**	**4.0**	**-1.0**	**4.0**	**2.0**	**4.0**	**-1.0**

* The numbers presented here for pharmacological and lifestyle therapies are, with the aid of expert opinion, derived from the evidence base set out in the section ‘Estimates of effects’ in the main text.

** In the case of the monitoring group, regression analysis techniques were used to estimate age-related BP changes from within the H2000 sample.

Table 9: Mini-Finland estimates of BP group effects on subsequent cardiovascular morbidity and mortality, by gender and age.

**Table of five-year intensities**	male		female	
	40–59 years old	60–79 years old	40–59 years old	60–79 years old
**CHD-related event**				
BPG 0	0.025	0.065	0.013	0.055
BPG 1	0.032	0.083	0.016	0.065
BPG 2	0.036	0.092	0.018	0.068
BPG 3	0.058	0.128	0.024	0.073
**CVE-related event**				
BPG 0	0.010	0.039	0.008	0.050
BPG 1	0.013	0.050	0.010	0.064
BPG 2	0.014	0.056	0.011	0.070
BPG 3	0.031	0.121	0.017	0.093
**CHD-related death**				
BPG 0	0.000	0.003	0.002	0.000
BPG 1	0.000	0.006	0.002	0.000
BPG 2	0.000	0.011	0.002	0.000
BPG 3	0.007	0.023	0.002	0.162
**CVE-related death**				
BPG 0	0.013	0.040	0.000	0.000
BPG 1	0.015	0.052	0.000	0.000
BPG 2	0.015	0.058	0.000	0.000
BPG 3	0.015	0.107	0.006	0.043
**death from other causes**				
BPG 0	0.024	0.126	0.006	0.073
BPG 1	0.029	0.151	0.008	0.089
BPG 2	0.032	0.159	0.012	0.094
BPG 3	0.037	0.174	0.018	0.104

Table 10: Mini-Finland predictions of the effect of morbidity at baseline on subsequent cardiovascular morbidity and mortality.

Table of five-year intensities	male		Female	
	40–59 years old	60–79 years old	40–59 years old	60–79 years old
**CHD-related event**				
no cardiovascular morbidity recorded at baseline	0.044	0.105	0.027	0.017
CVE-related morbidity recorded at baseline	0.063	0.259	0.018	0.050
CHD-related morbidity recorded at baseline	0.145	0.294	0.073	0.052
**CVE-related event**				
no cardiovascular morbidity recorded at baseline	0.023	0.035	0.003	0.087
CVE-related morbidity recorded at baseline	0.129	0.164	0.004	0.173
CHD-related morbidity recorded at baseline	0.041	0.043	0.006	0.092
**CHD-related death**				
no cardiovascular morbidity recorded at baseline	0.004	0.030	0.007	0.106
CVE-related morbidity recorded at baseline	0.009	0.098	0.040	0.400
CHD-related morbidity recorded at baseline	0.004	0.033	0.010	0.180
**CVE-related death**				
no cardiovascular morbidity recorded at baseline	0.017	0.089	0.003	0.037
CVE-related morbidity recorded at baseline	0.086	0.270	0.004	0.043
CHD-related morbidity recorded at baseline	0.082	0.262	0.006	0.085
**death from other causes**				
no cardiovascular morbidity recorded at baseline	0.035	0.195	0.031	0.120
CVE-related morbidity recorded at baseline	0.029	0.244	0.076	0.352
CHD-related morbidity recorded at baseline	0.042	0.205	0.029	0.131

Table 11: The numbers of people estimated to be in each of the subgroups stratified by age, gender, and BPG.

		blood pressure group classification			
**males**		**BPG 0**	**BPG 1**	**BPG 2**	**BPG 3**
age group	**70–74**	7,000	3,000	16,000	13,000
	**65–69**	7,000	6,000	19,000	20,000
	**60–64**	10,000	13,000	26,000	15,000
	**55–59**	22,000	17,000	35,000	20,000
	**50–54**	41,000	32,000	56,000	34,000
	**45–49**	48,000	39,000	49,000	22,000
	**40–44**	60,000	34,000	35,000	15,000
**females**		BPG 0	BPG 1	BPG 2	BPG 3
age group	**70–74**	7,000	6,000	21,000	30,000
	**65–69**	7,000	13,000	19,000	21,000
	**60–64**	18,000	13,000	26,000	26,000
	**55–59**	27,000	19,000	35,000	27,000
	**50–54**	65,000	32,000	37,000	25,000
	**45–49**	81,000	30,000	32,000	13,000
	**40–44**	100,000	24,000	20,000	9,000

References used in Table 1 (the brief glossary):

1. Culyer AJ: **The dictionary of health economics**. London: Edward Elgar; 2005.
2. Sugden R, Williams A: **The principles of practical cost-benefit analysis**. New York: Oxford University Press; 1978.
3. Detsky AS, Naglie G, Krahn MD, Naimark D, Redelmeier DA: **Primer on medical decision analysis: Part 1– getting started**. *Med Decis Making* 1997, **17**(2):123-125.
4. Krahn MD, Naglie G, Naimark D, Redelmeier DA, Detsky AS: **Primer on medical decision analysis: Part 4--Analyzing the model and interpreting the results**. *Med Decis Making* 1997, **17**(2):142-151.
5. Williams A: **The cost–benefit approach**. *Br Med Bull* 1974, **30**(3):252-256.
6. Dowie J: **No room for kinkiness in a public healthcare system**. *Pharmacoeconomics* 2005, **23**(12):1203-1205.
