# Supplementary material for: Cost-effectiveness analysis of guidelines for antihypertensive care in Finland
Source: BMC Health Serv Res. 2007 Oct 24;7:172. doi: 10.1186/1472-6963-7-172 (PMC2174470; doi:10.1186/1472-6963-7-172)
Supplement: Additional File 2 — Current Care guidelines. Supplementary information describing the Current Care guideline process. [file 1472-6963-7-172-S2.pdf]

In Finland, both local and national guidelines and care programmes have been produced at least since the late 1980s [1]. The guidelines identified in that study vary greatly in length, content and the evidence base used. Therefore, the remit of the Current Care organization was to develop evidence based clinical practice guidelines for the Finnish health care system and improve quality of care.

The Finnish Medical Society Duodecim, i.e., the main Finnish medical society, took the lead in guideline development. It was deemed essential to involve the national specialist societies. These were invited to join the initiative and other stakeholders also gave their support. The production of Current Care Guidelines began in 1994 [2]. A chairperson representing the relevant clinical speciality was selected for the guideline development group, and a Current Care editor was appointed. From the outset, care was taken to always include a general practitioner, since the majority of the guidelines were intended for use both in specialized care and in primary care. To ensure good methodological basis and a similar basic structure for all Current Care guidelines, a guideline developer's handbook was produced and published both in print and on the Internet.

The development process of a Current Care guideline is outlined in Table 1. An experienced medical librarian assists the guideline development group in systematic literature searches starting from quality-rated sources such as the Cochrane Library and expanding to Medline, locating systematic reviews produced elsewhere. Guidelines developed elsewhere are always identified, in order not to duplicate work which can usefully be used. The obtained literature is critically appraised, using the tools available from the Evidence Based Medicine Working Group, an evolved version of which can be found in [3]. Depending on the quality and size of the original studies, the strength of the main statements is graded from A to D. **Level A** represents 'Strong research-based evidence', that is multiple, relevant, high-quality studies with homogenous results (e.g. two or more randomised controlled trials, or other most suitable research design), or a systematic review with clear results. **Level B** represents 'Moderate evidence' (e.g. one randomised controlled trial, or multiple adequate studies), **Level C** 'Limited research-based evidence' (e.g. controlled prospective studies) and **Level D** 'No evidence' (none of the above available, e.g. only retrospective studies, or the consensus reached by the group in the absence of good quality evidence) [4].

Based on the evidence and discussions in the guideline group, a draft guideline is developed that is then widely circulated to identified stakeholders for comments. Whenever there are active patient organizations in the field of the guideline, their views can be included during the comment round. The guideline development group carefully reviews the comments received. Changes are made accordingly, when additional evidence is brought up, and especially to make the statements in the guideline clear to the potential audience.

The Current Care guidelines are published in hard copy format in the Medical Journal Duodecim. From the outset, the distinguishing features of Current Care guidelines were electronic publication and evidence summaries. The electronic publication format had been developed for the collection of thorough, primary care-oriented guidelines that is today the Evidence Based Medicine Guidelines [5]. The main recommendations in a Current Care guideline are backed up by an evidence summary

in which the available best evidence is summarised for the interested reader. Electronic publication, first on CD-ROMs and now on the Internet, was seen as the main media for publication since it enabled wide dissemination and ease of linkage to the evidence base.

The first Current Care guideline on celiac disease and was published in 1997. By the end of 2004, 53 guidelines had been published in Finnish, as well as a few also in English (glaucoma [6], eating disorders [7]; smoking cessation [4]). In these cases, the guideline development groups have been active to translate and publish their work more widely. More recently, summary versions of some guidelines have been translated to English and can be accessed freely on the Internet (www.kaypahoito.fi > in English), e.g., the guidelines on childhood obesity, pulmonary embolism, treatment of alcohol abuse, and hypertension (updated 2005).

Table 1. An outline of the drawing up of a Current Care Guideline.

*A topic is suggested (most commonly by a specialist medical society)
*The topic is chosen (by the board of Current Care according to systematic criteria)
*The working group is assembled (chairperson, editor, other members)
*The group receives appropriate critical appraisal training
*Systematic search of the literature (medical librarian)
*Drawing up the evidence summaries and then statements based on the available research (the experts – the main body of work)
*Drawing up the guideline text based on the evidence summaries
*Circulating the draft guideline for critical comments
*Publication of the guideline
-Internet: www.kaypahoito.fi, no password required
-Lääkärin tietokannat (Evidence Based Medicine Guideline Finnish version) available via a widely used health portal www.terveysportti.fi
-Medical Journal Duodecim
-a version for the lay public in the leading health magazine in Finland (Hyvä Terveys)
-in addition, other health professionals outline the guidelines from their perspective in their respective journals
*Updating
-minor updates as significant evidence accumulates
-major updates at set intervals of about three years
-publication in electronic formats and a short report of significant guideline changes in the Medical Journal Duodecim

References used in the description of Current Care guidelines:

1. Varonen H, Mäkelä M: **Practice guidelines in Finland: availability and quality**. *Qual Health Care* 1997, **6**(2):75-79.
2. Ketola E, Kaila M, Makela M: **[Current care guidelines from trials to keystone]**. *Duodecim* 2004, **120**(24):2949-2954.
3. Guyatt G, Rennie D: **Users' guides to the medical literature: Essentials of evidence-based clinical practice**. Chicago (Ill.): AMA Press; 2002.
4. Winell K, Kaila M, Mäkelä M: **Finnish Current Care Guidelines now target tobacco cessation**. *Suomen Lääkärilehti* 2003, **58**:2983-2984.
5. Varonen H, Jousimaa J, Helin-Salmivaara A, Kunnamo I: **Electronic primary care guidelines with links to Cochrane reviews--EBM Guidelines**. *Fam Pract* 2005, **22**(4):465-469.
6. Tuulonen A, Airaksinen PJ, Erola E, Forsman E, Friberg K, Kaila M, Klemetti A, Makela M, Oskala P, Puska P *et al*: **The Finnish evidence-based guideline for open-angle glaucoma**. *Acta Ophthalmol Scand* 2003, **81**(1):3-18.
7. Ebeling H, Tapanainen P, Joutsenoja A, Koskinen M, Morin-Papunen L, Jarvi L, Hassinen R, Keski-Rahkonen A, Rissanen A, Wahlbeck K: **A practice guideline for treatment of eating disorders in children and adolescents**. *Ann Med* 2003, **35**(7):488-501.
