# Supplementary material for: Cost-effectiveness analysis of guidelines for antihypertensive care in Finland
Source: BMC Health Serv Res. 2007 Oct 24;7:172. doi: 10.1186/1472-6963-7-172 (PMC2174470; doi:10.1186/1472-6963-7-172)

Figure 1: Flowchart for the comparison of the ACCG and PCP scenarios.

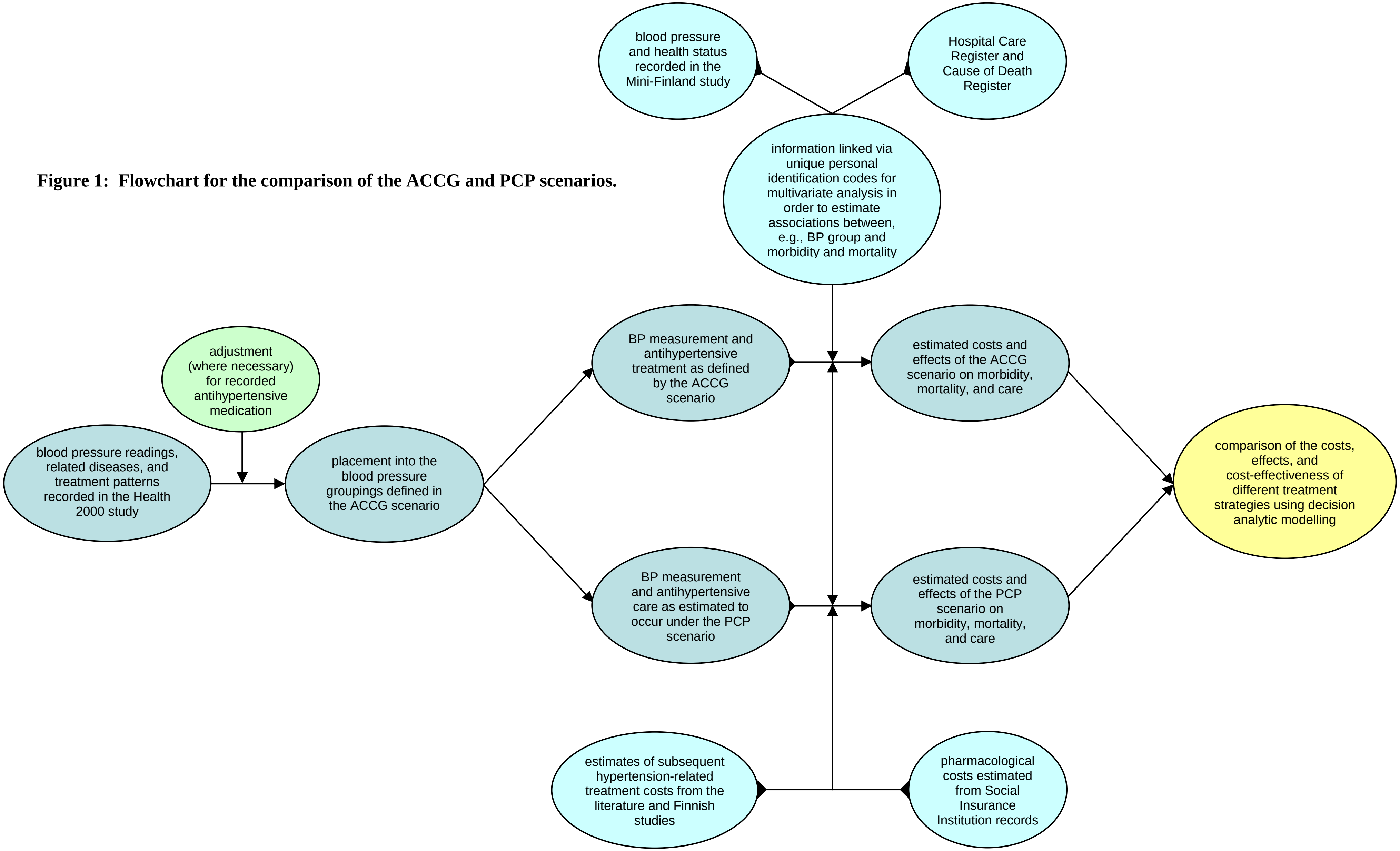

Figure 2: State transition diagram

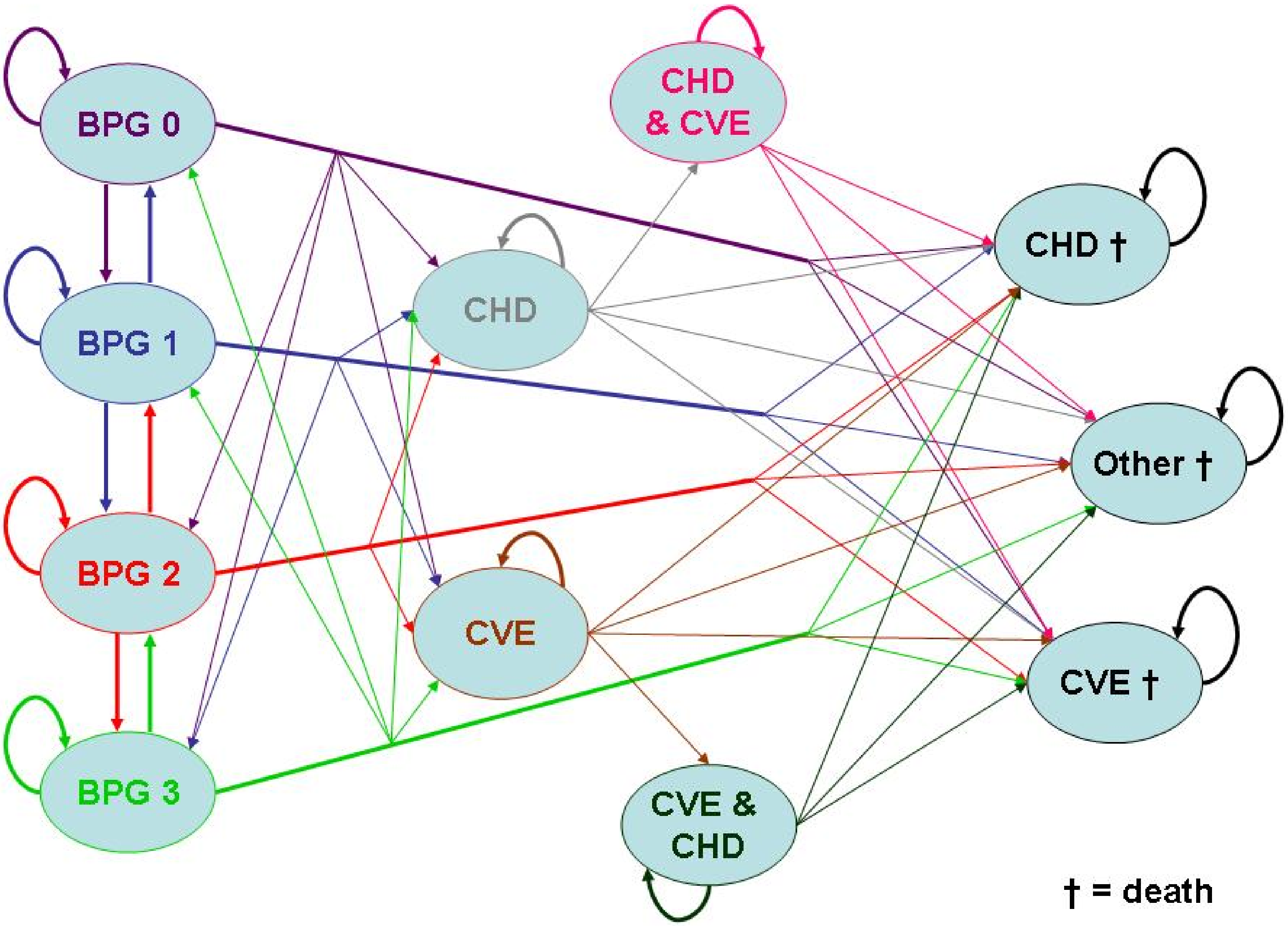

Figure 3: Outline of decision model

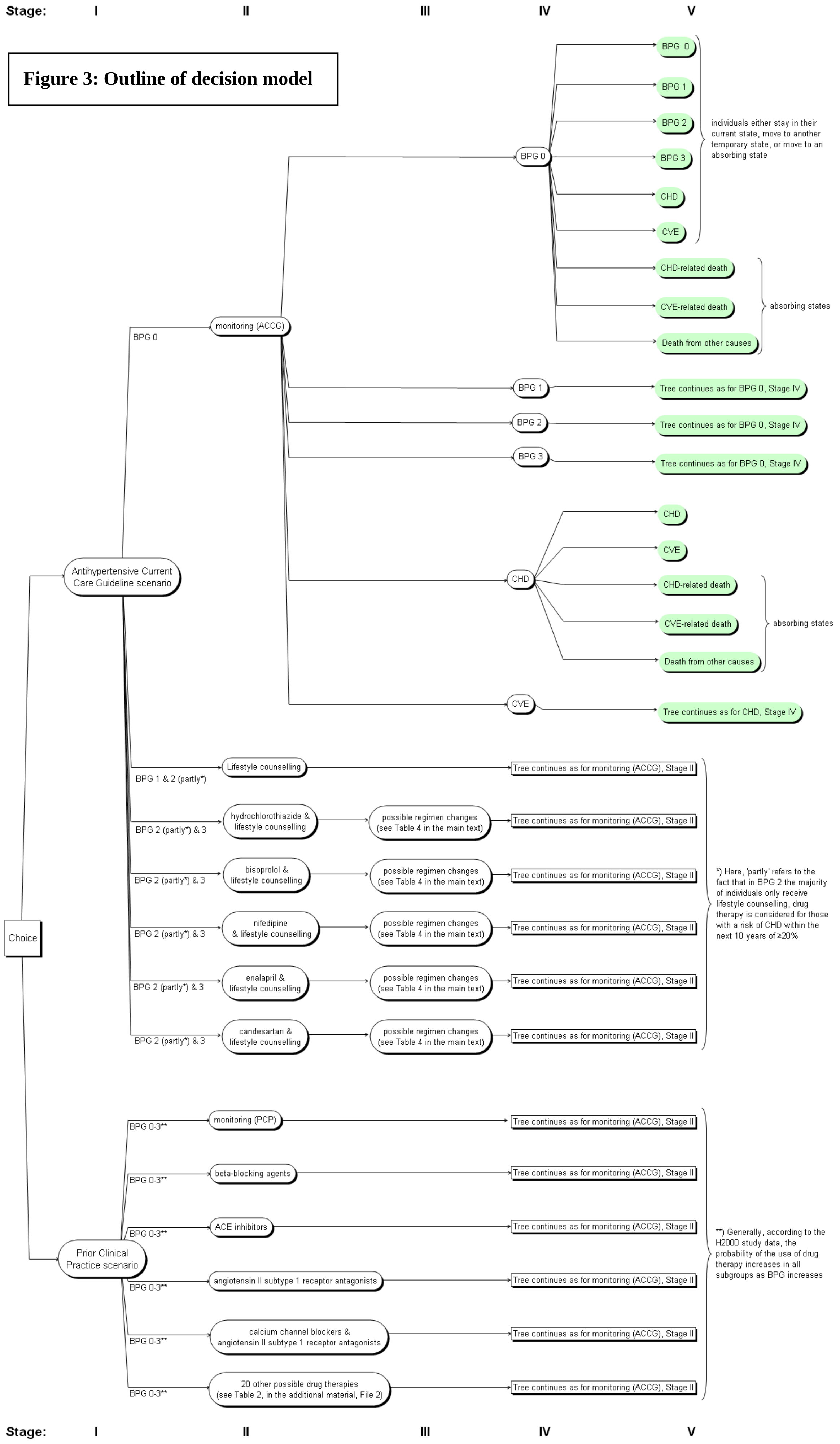

Supplement: Additional File 3 — Extra Figures. Supplementary figures. [file 1472-6963-7-172-S3.pdf]
