# Supplementary material for: Cost-effectiveness analysis of guidelines for antihypertensive care in Finland
Source: BMC Health Serv Res. 2007 Oct 24;7:172. doi: 10.1186/1472-6963-7-172 (PMC2174470; doi:10.1186/1472-6963-7-172)
Supplement: Additional File 4 — Details of cost calculations. Detailed calculations of the non-pharmacological treatment-related costs for the ACCG and PCP scenarios. [file 1472-6963-7-172-S4.pdf]

Detailed calculations of the non-pharmacological treatment-related costs for the ACCG and PCP scenarios **Additional File 4**

Table 1: Diagnostic BP measurement resource use, yearly, by scenario.

Scenario:	resource used	frequency	proportion	mean cost per minute	time (minutes)	total cost
ACCG	nurse	4	1.0	0.28 €	13	14.73 €
PCP	nurse	4	0.8	0.28 €	5	5.17 €
	physician	1	0.2	0.63 €	5	

Table 2: Diagnostic workup

	Cost per unit (€)			Time (minutes)	Mean cost (€)	Probability procedure is used in PCP diagnosis		PCP total costs (male)	PCP total costs (female)	Probability procedure is used in ACCG diagnosis	ACCG total costs
	Nurse time	Physician time	Procedure			Male	Female				
Anamnesis:											
Related diseases		0,63		1	0,6	1	1			1	
BP history		0,63		2	1,3	1	1			1	
Current medication		0,63		1	0,6	1	1			1	
Lifestyle (ACCG)	0,28			6	1,7	0	0			1	
Lifestyle (PCP)		0,63		2	1,3	1	1			0	
Psychological and social factors		0,63		2	1,3	1	1			1	
Family history		0,63		1	0,6	1	1			1	
								5,70	5,70		6,13
Clinical examination:											
Inspection		0,63		2	1,3	1	1			1	
Palpation		0,63		2	1,3	1	1			1	
Auscultation		0,63		2	1,3	1	1			1	
Weight, height, BMI, waist circumference	0,28			6	1,7	1	1			1	
								5,50	5,50		5,50
Laboratory tests:											
Basic blood analysis			1,5			0,2	0,05			1	
Proteinuria			1,6			1	1			1	
Serum creatinine			1,5			1	1			1	
Serum potassium			1,5			1	1			1	
Serum glutamyltransferase			1,5			0,2	0,05			0	
Serum lipid analysis			5			1	1			1	
Plasma glucose			1,5			1	1			1	
Electrocardiogram			8,1			1	1			1	
								19,80	19,35		20,70
Imaging diagnostics:											
Chest X-ray, basic			27,1			1	1			0,1	
Ultrasound cardiovascular investigations			53,6			0,05	0,05	29,78	29,78	0,05	5,39
								60,78	60,33		37,72

Detailed calculations of the non-pharmacological treatment-related costs for the ACCG and PCP scenarios

Table 3: Costs of ACCG lifestyle intervention (based on expert opinion and [1]).

			(where applicable)	(where applicable)		
	**freq.**	**resource**	**time (minutes)**	**mean cost per minute (€)**	**item totals**	**Totals (€)**
**First-year costs**						
group education (per patient)	1	physician	0.9	0.64	0.58	
	1	nutritionist	0.9	0.32	0.29	
group sessions (per patient)	1	physician	6	0.64	3.84	
	1	nutritionist	6	0.32	1.90	
health centre visits	4	nurse	100	0.28	28.33	
published material	1	printing costs			0.80	
						35.73
**Second-year costs**						
education (per patient)	1	physician	0.9	0.64	0.58	
	1	nutritionist	0.9	0.32	0.29	
group sessions (per patient)	1	physician	6	0.64	3.84	
	1	nutritionist	6	0.32	1.90	
health centre visits	3	nurse	60	0.28	17.00	
published material	1	printing costs			0.30	
						23.90
**Subsequent years' costs**						
health centre visits	3	nurse	60	0.28	17.00	
published material	1	printing costs			0.20	
						17.20

Table 4: Follow-up BP measurement resource use (ACCG).

CCG								
	assumed freq. of follow-up sessions per year	resource	prop. (%)	mean cost per min. (€)	time (min.)	cost	Totals per year (€)	per 5-years (€)
S0	0.5	nurse	100	0.28	13	1.84		
	0.2	physician	100	0.63	10	1.27	3.11	15.54
S1	1	nurse	100	0.28	13	3.68		
	1	physician	100	0.63	10	6.33	10.02	50.08
S2	4	nurse	100	0.28	13	14.73		
	2	physician	100	0.63	10	12.67	27.40	137.00
S3	8	nurse	100	0.28	13	29.47		
	4	physician	100	0.63	10	25.33		
	1	specialist	30	0.58	13	2.28	57.08	285.38

The frequency of follow-up BP measurement for the PCP scenario is approximated via responses from the H2000 study for the PCP scenario [2]. Regression methods were used to obtain the estimates in Table 5 and Table 6 (further details available from the authors).

Table 5: Observed BP measurement resource use (PCP, males) in €.

male PCP							Totals per year	5-year
	estimated freq. of sessions per year	resource	prop. (%)	mean cost per min. (€)	time (min.)	cost	Totals per year (€)	per 5-years (€)
S0	0.0328	nurse	100	0.28	13	0.12		
	0.0009	physician	100	0.63	10	0.01	0.13	0.63
3								
S1	0.8007	nurse	100	0.28	13	2.95		
	0.2390	physician	100	0.63	10	1.51	4.46	22.31
3								
S2	1.2047	nurse	100	0.28	13	4.44		
	0.3823	physician	100	0.63	10	2.42	6.86	34.29
3								
S3	2.1639	nurse	100	0.28	13	7.97		
	0.5724	physician	100	0.63	10	3.63		
	1.0000	specialist	30	0.58	13	2.28	13.87	69.35

Table 6: Observed BP measurement resource use (PCP, females) in €.

female PCP							Totals per year	5-year
	estimated freq. of sessions per year	resource	prop. (%)	mean cost per min. (€)	time (min.)	cost	Totals per year (€)	per 5-years (€)
S0	0.3967	nurse	100	0.28	13	1.46		
	0.1031	physician	100	0.63	10	0.65	2.11	10.57
S1	1.1646	nurse	100	0.28	13	4.29		
	0.3412	physician	100	0.63	10	2.16	6.45	32.25
S2	1.5686	nurse	100	0.28	13	5.78		
	0.4846	physician	100	0.63	10	3.07	8.85	44.23
S3	2.5277	nurse	100	0.28	13	9.31		
	0.6746	physician	100	0.63	10	4.27		
	1.0000	specialist	30	0.58	13	2.28	15.86	79.29

References used in the detailed cost calculations:

1. Kastarinen MJ, Puska PM, Korhonen MH, Mustonen JN, Salomaa VV, Sundvall JE, Tuomilehto JO, Uusitupa MI, Nissinen AM: **Non-pharmacological treatment of hypertension in primary health care: a 2-year open randomized controlled trial of lifestyle intervention against hypertension in eastern Finland**. *J Hypertens* 2002, **20**(12):2505-2512.
2. Aromaa A, Koskinen S (eds.): **Health and functional capacity in Finland : baseline results of the Health 2000 health examination survey**. Helsinki: Publications of the National Public Health Institute, B12; 2004.
