# Supplementary material for: Cost-effectiveness analysis of guidelines for antihypertensive care in Finland
Source: BMC Health Serv Res. 2007 Oct 24;7:172. doi: 10.1186/1472-6963-7-172 (PMC2174470; doi:10.1186/1472-6963-7-172)
Supplement: Additional File 5 — Sensitivity analysis. Full results of the sensitivity analysis undertaken on subgroups. [file 1472-6963-7-172-S5.pdf]

When the evidence base for certain model parameters was found to be of unknown quality, pragmatism resulted in the use of subjective estimates. Uncertainty surrounding both objective evidence and subjective input can only be partially dealt with using the deterministic sensitivity analysis employed here. The impact of various alternative assumptions is explored by performing sensitivity analyses; however, probabilistic sensitivity analysis is considered to be beyond the scope of this study. Many parameters were not considered to be readily amenable to probabilistic sensitivity analysis, due to the paucity of information concerning appropriate distributions.

| | |
|---|---|
| (red) | ACCG less effective and more costly than PCP |
| (yellow) | ACCG more effective and more costly than PCP |
| (orange) | ACCG less effective and less costly than PCP |
| (green) | ACCG more effective and less costly than PCP |

**Key for tables of results**

The above key is used in the tables of sensitivity analysis results (below) to highlight the quadrants of the cost-effectiveness plane in which the subgroup-specific ICERs are located.

**References used in Table 1 below.**


1. Evers SM, Struijs JN, Ament AJ, van Genugten ML, Jager JH, van den Bos GA: **International comparison of stroke cost studies**. *Stroke* 2004, **35**(5):1209-1215.
2. Payne KA, Huybrechts KF, Caro JJ, Craig Green TJ, Klittich WS: **Long term cost-of-illness in stroke: an international review**. *Pharmacoeconomics* 2002, **20**(12):813-825.
3. Zethraeus N, Molin T, Henriksson P, Jonsson B: **Costs of coronary heart disease and stroke: the case of Sweden**. *JInternMed* 1999, **246**(2):151-159.
4. Russell MW, Huse DM, Drowns S, Hamel EC, Hartz SC: **Direct medical costs of coronary artery disease in the United States**. *Am J Cardiol* 1998, **81**(9):1110-1115.
5. Kastarinen MJ, Puska PM, Korhonen MH, Mustonen JN, Salomaa VV, Sundvall JE, Tuomilehto JO, Uusitupa MI, Nissinen AM: **Non–pharmacological treatment of hypertension in primary health care: a 2-year open randomized controlled trial of lifestyle intervention against hypertension in eastern Finland**. *J Hypertens* 2002, **20**(12):2505-2512.
6. Hujanen T: **Terveydenhuollon yksikkökustannukset Suomessa vuonna 2001**. In.: Stakes. Aiheita 1/2003.; 2003.

**Table 1: Variables or variable information for the base case analysis.**

| **Variables or parameters** | Value in **base case** (in € unless otherwise stated) | | **Source** |
|---|---|---|---|
| Morbidity costs: | | | |
| CVE (one year) | 3,000 | | [1], [2], and expert opinion |
| CVE (initial) | 0 | | expert opinion |
| CHD (one year) | 1,000 | | [3], [4], and expert opinion |
| CHD (initial) | 0 | | expert opinion |
| Medication costs (**one year**): | | | |
| | **Female** | **Male** | |
| C03AA03 (**ACCG**) | 21 | 20 | Finnish Social Insurance Institution |
| C03EA (**PCP**) | 37 | 36 | Finnish Social Insurance Institution |
| C07A (**PCP**) | 121 | 129 | Finnish Social Insurance Institution |
| C07AB07 (**ACCG**) | 95 | 100 | Finnish Social Insurance Institution |
| C07B (**PCP**) | 125 | 125 | Finnish Social Insurance Institution |
| C07F (**PCP**) | 244 | 253 | Finnish Social Insurance Institution |
| C08 (**PCP**) | 203 | 218 | Finnish Social Insurance Institution |
| C08CA05 (**ACCG**) | 202 | 216 | Finnish Social Insurance Institution |
| C09A (**PCP**) | 171 | 180 | Finnish Social Insurance Institution |
| C09AA02 (**ACCG**) | 161 | 171 | Finnish Social Insurance Institution |
| C09BA (**PCP**) | 203 | 207 | Finnish Social Insurance Institution |
| C09BB (**PCP**) | 309 | 310 | Finnish Social Insurance Institution |
| C09C (**PCP**) | 222 | 223 | Finnish Social Insurance Institution |
| C09CA02 (**ACCG**) | 104 | 103 | Finnish Social Insurance Institution |
| C09D (**PCP**) | 244 | 241 | Finnish Social Insurance Institution |
| Lifestyle intervention costs (**ACCG** only): | | | |
| first year | 86 | | [5] |
| subsequent four-year period | 111 | | [5] |
| five-year transition probabilities, for BPG transitions | a table, by **Gen**, **BPG**, and **Age** | | H2000, the evidence set out in the section *Estimates of effects* in the main text, and expert opinion |
| five-year transition probabilities, for transitions to morbid states from morbidity-free states | a table, by **Gen**, **BPG**, and **Age2** | | MF combined with the Finnish Cause of Death and Hospital Care registers |
| five-year transition probabilities, for transitions to morbid states from a state of morbidity | a table, by **Gen** and **Age2** | | MF combined with the Finnish Cause of Death and Hospital Care registers |
| Other parameters: | | | |
| diagnostic costs (once only) | **PCP** = 62 and **ACCG** = 38 | | [6] and expert opinion |
| drug therapy switching (**ACCG** only) | 45% (percentage) | | expert opinion |
| initial drug therapy (**ACCG**) | 60% (percentage) | | expert opinion |
| recorded use of antihypertensives (**PCP**) | a table, by **Age2** and **BPG** | | H2000 |
| recorded use of classes of antihypertensives (**PCP**) | a table, by **Age**, **BPG**, and **Gen** | | Finnish Social Insurance Institution |
| annual discount rate, costs | 5% (percentage) | | Health Insurance Act, Section 5a |
| annual discount rate, benefits | 5% (percentage) | | Health Insurance Act, Section 5a |
| BPG HRQL weights | (not used in base case) | | H2000 |
| CVE morbidity HRQL weights | (not used in base case) | | H2000 |
| CHD morbidity HRQL weights | (not used in base case) | | H2000 |
| costs of anamnesis (one year, only if BPG > 1 in **ACCG** condition) | 44 | | [6] and expert opinion |

## Key:

| | | |
|---|---|---|
| **Age** = stratified by five-year age group | **Age2** = stratified by two age groups (40–59 and 60–79 years) | |
| **ACCG** = ACCG scenario | **PCP** = PCP scenario | |
| **BPG** = stratified by BPG | **Tre** = treatment | **Gen** = stratified by gender |

**Table 2: The values used in sensitivity analysis**

| | | | | | | |
|---|---|---|---|---|---|---|
| **effects of antihypertensive care on BP** | estimated effects of pharmacological treatment are that of 'full fraction of effect' | estimated effects of pharmacological treatment 20% less than in the base case | estimated effects of pharmacological treatment 50% less than in the base case | estimated effects of pharmacological treatment 20% more than in the base case | estimated effect of ageing on BP is 50% of that in the base case | |
| **HRQL** | no adjustment for health-related quality of life | adjustment for health-related quality of life for resultant morbidity and for BP group status | | | | |
| **lifestyle intervention costs (ACCG)** | 1st year = 36€ 2nd year = 24€ subsequent years = 17€ | Lifestyle intervention costs 50% of those used in base case | Lifestyle intervention costs 150% of those used in base case | Lifestyle intervention costs 200% of those used in base case | Lifestyle intervention costs 400% of those used in base case | |
| **medication costs** | SII and user costs (VAT deducted) | SII costs only (VAT deducted) | SII and user costs (includes estimated effect of generic substitution on prices, VAT deducted) | SII costs only (includes estimated effect of generic substitution on prices, VAT deducted) | SII and user costs (no VAT deducted) | PCP prices identically equal to ACCG prices |
| **morbidity costs** | yearly cost of CV 3,000€, yearly cost of CHD 1,000€ | initial cost of CV 3,000€, yearly cost 3,000€. Initial cost of CHD 1,000€, yearly cost 1,000€ | initial cost of CV 7,500€, yearly cost 1,500€. Initial cost of CHD 2,500€, yearly cost 500€ | initial cost of CV 10,000€, yearly cost 10,000€. Initial cost of CHD 5,000€, yearly cost 3,000€ | initial cost of CV 5,000€, yearly cost 3,000€. Initial cost of CHD 1,500€, yearly cost 1,000€ | no costs associated with morbidity |
| **switching medications (ACCG)** | switching between alternative drug therapies occurs at a rate of 45% | switching between alternative drug therapies occurs at a rate of 25% | switching between alternative drug therapies occurs at a rate of 65% | switching between alternative drug therapies occurs at a rate of 85% | | |
| **distribution of first line therapy (ACCG)** | 60% of initial pharmacological therapies are thiazide diuretics - ACE inhibitors, angiotensin II antagonists, beta blocking agents and calcium channel blockers are the equally likely alternatives | 20% of initial pharmacological therapies are thiazide diuretics - ACE inhibitors, angiotensin II antagonists, beta blocking agents and calcium channel blockers are the equally likely alternatives | 40% of initial pharmacological therapies are thiazide diuretics - ACE inhibitors, angiotensin II antagonists, beta blocking agents and calcium channel blockers are the equally likely alternatives | 80% of initial pharmacological therapies are thiazide diuretics - ACE inhibitors, angiotensin II antagonists, beta blocking agents and calcium channel blockers are the equally likely alternatives | 100% of initial pharmacological therapies are thiazide diuretics | |
| **discount rates** | benefit discount rate = 5%, cost discount rate = 5% | benefit discount rate = 0%, cost discount rate = 0% | benefit discount rate = 3%, cost discount rate = 3% | benefit discount rate = 3%, cost discount rate = 6% | | |

**(base case values in shaded cells)**

**base case analysis (see shaded section of table 'The values used in sensitivity analysis')**

blood pressure group classification

**males**

| age group | | BPG 0 | BPG 1 | BPG 2 | BPG 3 |
|---|---|---|---|---|---|
| 70–74 | | €217 000/LY | €74 000/LY | ACCG dominates | ACCG dominated |
| | | - €5m | - €1m | - €5m | €2m |
| | | -23 LY | -15 LY | 19 LY | -65 LY |
| 65–69 | | €103 000/LY | €32 000/LY | ACCG dominates | €7 000/LY |
| | | - €6m | - €4m | - €6m | €3m |
| | | -56 LY | -110 LY | 312 LY | 395 LY |
| 60–64 | | €42 000/LY | €9 000/LY | ACCG dominates | €3 000/LY |
| | | - €9m | - €8m | - €16m | €2m |
| | | -223 LY | -860 LY | 223 LY | 678 LY |
| 55–59 | | €15,000/LY | ACCG dominates | ACCG dominates | €3,000/LY |
| | | - €11m | - €3m | - €12m | €9m |
| | | -746 LY | 146 LY | 1 847 LY | 3 420 LY |
| 50–54 | | €42,000/LY | ACCG dominates | ACCG dominates | €5,000/LY |
| | | - €24m | - €7m | - €25m | €23m |
| | | -572 LY | 1 064 LY | 3,366 LY | 4,476 LY |
| 45–49 | | €34,000/LY | ACCG dominates | ACCG dominates | €10,000/LY |
| | | - €29m | - €10m | - €25m | €20m |
| | | -841 LY | 1 705 LY | 1 171 LY | 2,040 LY |
| 40–44 | | €42,000/LY | ACCG dominates | ACCG dominates | €6,000/LY |
| | | - €38m | - €7m | - €18m | €11m |
| | | -904 LY | 891 LY | 287 LY | 1,898 LY |

**females**

| age group | | BPG 0 | BPG 1 | BPG 2 | BPG 3 |
|---|---|---|---|---|---|
| 70–74 | | ACCG dominates | ACCG dominates | ACCG dominates | €13,000/LY |
| | | - €3m | - €5m | - €11m | €7m |
| | | 4 LY | 15 LY | 332 LY | 567 LY |
| 65–69 | | €95,000/LY | €48,000/LY | €32,000/LY | €4,000/LY |
| | | - €3m | - €12m | - €12m | €6m |
| | | -36 LY | -247 LY | -359 LY | 1,327 LY |
| 60–64 | | €50,000/LY | €20,000/LY | ACCG dominates | €3,000/LY |
| | | - €10m | - €14m | - €19m | €7m |
| | | -193 LY | -699 LY | 54 LY | 2,559 LY |
| 55–59 | | €30 000/LY | ACCG dominates | ACCG dominates | €1,000/LY |
| | | - €13m | - €5m | - €24m | €18m |
| | | -435 LY | 574 LY | 1 387 LY | 12,756 LY |
| 50–54 | | €42 000/LY | ACCG dominates | €55,000/LY | €2,000/LY |
| | | - €35m | - €9m | - €29m | €18m |
| | | -843 LY | 1,404 LY | -527 LY | 7,388 LY |
| 45–49 | | €69 000/LY | ACCG dominates | €15,000/LY | €3,000/LY |
| | | - €48m | - €10m | - €28m | €10m |
| | | -698 LY | 1,892 LY | -1 804 LY | 3 191 LY |
| 40–44 | | €52 000/LY | ACCG dominates | €45 000/LY | €4,000/LY |
| | | - €60m | - €7m | - €18m | €7m |
| | | -1,137 LY | 1,734 LY | -397 LY | 1 812 LY |

| | | | |
|---|---|---|---|
| aggregated incremental cost-effectiveness ratio | ACCG dominates | | |
| aggregated incremental costs | - €498m | -300 | euros per person* |
| aggregated incremental effects | 49,000 LY | 12 | days per person* |

*averaged figure in the target population (1.5 million Finns) over the period of study (10-40 years)

**Estimated effects of pharmacological treatment 20% less than in the base case**

**males**

| | blood pressure group classification | | | |
|---|---|---|---|---|
| age group | BPG 0 | BPG 1 | BPG 2 | BPG 3 |
| 70–74 | €217 000/LY | €84 000/LY | ACCG dominates | ACCG dominated |
| | - €5m | - €1m | - €4m | €3m |
| | -23 LY | -13 LY | 7 LY | -51 LY |
| 65–69 | €103 000/LY | €46 000/LY | ACCG dominates | €12 000/LY |
| | - €6m | - €4m | - €5m | €4m |
| | -56 LY | -77 LY | 258 LY | 308 LY |
| 60–64 | €42 000/LY | €12 000/LY | ACCG dominates | €6 000/LY |
| | - €9m | - €8m | - €15m | €3m |
| | -223 LY | -678 LY | 200 LY | 476 LY |
| 55–59 | €15,000/LY | ACCG dominates | ACCG dominates | €5,000/LY |
| | - €11m | - €3m | - €11m | €12m |
| | -746 LY | 242 LY | 1 985 LY | 2 436 LY |
| 50–54 | €42,000/LY | ACCG dominates | ACCG dominates | €8,000/LY |
| | - €24m | - €7m | - €24m | €29m |
| | -572 LY | 1 223 LY | 3,701 LY | 3,467 LY |
| 45–49 | €34,000/LY | ACCG dominates | ACCG dominates | €14,000/LY |
| | - €29m | - €10m | - €24m | €23m |
| | -841 LY | 1 982 LY | 1 488 LY | 1,613 LY |
| 40–44 | €42,000/LY | ACCG dominates | ACCG dominates | €11,000/LY |
| | - €38m | - €7m | - €18m | €15m |
| | -904 LY | 1 114 LY | 585 LY | 1,297 LY |

**females**

| age group | BPG 0 | BPG 1 | BPG 2 | BPG 3 |
|---|---|---|---|---|
| 70–74 | ACCG dominates | ACCG dominates | ACCG dominates | €21,000/LY |
| | - €3m | - €5m | - €11m | €8m |
| | 4 LY | 13 LY | 326 LY | 376 LY |
| 65–69 | €95,000/LY | €70,000/LY | €37,000/LY | €9,000/LY |
| | - €3m | - €12m | - €12m | €6m |
| | -36 LY | -175 LY | -313 LY | ,664 LY |
| 60–64 | €50,000/LY | €23,000/LY | ACCG dominates | €3,000/LY |
| | - €10m | - €14m | - €19m | €8m |
| | -193 LY | -599 LY | 245 LY | 2,529 LY |
| 55–59 | €30 000/LY | ACCG dominates | ACCG dominates | €3,000/LY |
| | - €13m | - €5m | - €24m | €19m |
| | -435 LY | 746 LY | 1 973 LY | 6,047 LY |
| 50–54 | €42 000/LY | ACCG dominates | ACCG dominates | €3,000/LY |
| | - €35m | - €9m | - €29m | €20m |
| | -843 LY | 1,796 LY | 233 LY | 5,798 LY |
| 45–49 | €69 000/LY | ACCG dominates | €28,000/LY | €5,000/LY |
| | - €48m | - €10m | - €28m | €12m |
| | -698 LY | 2,129 LY | -1 006 LY | 2 474 LY |
| 40–44 | €52 000/LY | ACCG dominates | ACCG dominates | €6,000/LY |
| | - €60m | - €7m | - €18m | €8m |
| | -1,137 LY | 1,954 LY | 156 LY | 1 531 LY |

| | | | |
|---|---|---|---|
| aggregated incremental cost-effectiveness ratio | ACCG dominates | | |
| aggregated incremental costs | - €469m | -300 | euros per person* |
| aggregated incremental effects | 42,000 LY | 10 | days per person* |

*averaged figure in the target population (1.5 million Finns) over the period of study (10-40 years)

**Estimated effects of pharmacological treatment 50% less than in the base case**

blood pressure group classification

**males**

| age group | BPG 0 | BPG 1 | BPG 2 | BPG 3 |
|---|---|---|---|---|
| 70–74 | €217 000/LY | €146 000/LY | €603,000/LY | ACCG dominated |
| | - €5m | - €1m | - €2m | €3m |
| | -23 LY | -8 LY | -3 LY | -24 LY |
| 65–69 | €133 000/LY | €412 000/LY | ACCG dominates | €22 000/LY |
| | - €6m | - €4m | - €2m | €5m |
| | -43 LY | -9 LY | 154 LY | 205 LY |
| 60–64 | €55 000/LY | €24 000/LY | ACCG dominates | €25 000/LY |
| | - €9m | - €8m | - €12m | €4m |
| | -170 LY | -341 LY | 164 LY | 159 LY |
| 55–59 | €16,000/LY | ACCG dominates | ACCG dominates | €10,000/LY |
| | - €11m | - €3m | - €7m | €15m |
| | -702 LY | 287 LY | 1 831 LY | 1 427 LY |
| 50–54 | €44,000/LY | ACCG dominates | ACCG dominates | €23,000/LY |
| | - €24m | - €6m | - €17m | €34m |
| | -539 LY | 844 LY | 2,888 LY | 1,508 LY |
| 45–49 | €38,000/LY | ACCG dominates | ACCG dominates | €38,000/LY |
| | - €29m | - €8m | - €20m | €26m |
| | -755 LY | 1 419 LY | 1 033 LY | ,680 LY |
| 40–44 | €47,000/LY | ACCG dominates | ACCG dominates | €20,000/LY |
| | - €38m | - €7m | - €16m | €17m |
| | -806 LY | 1 156 LY | 637 LY | ,836 LY |

**females**

| age group | BPG 0 | BPG 1 | BPG 2 | BPG 3 |
|---|---|---|---|---|
| 70–74 | ACCG dominates | ACCG dominates | ACCG dominates | €49,000/LY |
| | - €3m | - €5m | - €11m | €8m |
| | 4 LY | 9 LY | 19 LY | 167 LY |
| 65–69 | €118,000/LY | €151,000/LY | €22,000/LY | €21,000/LY |
| | - €3m | - €13m | - €11m | €6m |
| | -29 LY | -83 LY | -522 LY | ,313 LY |
| 60–64 | €50,000/LY | €29,000/LY | €74 000/LY | €13,000/LY |
| | - €10m | - €14m | - €19m | €8m |
| | -193 LY | -482 LY | -259 LY | ,628 LY |
| 55–59 | €31 000/LY | ACCG dominates | €64,000/LY | €4,000/LY |
| | - €13m | - €4m | - €24m | €19m |
| | -420 LY | 356 LY | -379 LY | 5,097 LY |
| 50–54 | €50 000/LY | ACCG dominates | €130,000/LY | €6,000/LY |
| | - €35m | - €8m | - €28m | €21m |
| | -699 LY | 1,358 LY | -219 LY | 3,434 LY |
| 45–49 | €76 000/LY | ACCG dominates | €43,000/LY | €8,000/LY |
| | - €48m | - €8m | - €27m | €12m |
| | -637 LY | 1,003 LY | -638 LY | 1 516 LY |
| 40–44 | €56 000/LY | ACCG dominates | ACCG dominates | €9,000/LY |
| | - €60m | - €7m | - €17m | €9m |
| | -1,059 LY | 1,394 LY | 11 LY | 1 057 LY |

| | | |
|---|---|---|
| aggregated incremental cost-effectiveness ratio | ACCG dominates | |
| aggregated incremental costs | - €419m | -300 euros per person* |
| aggregated incremental effects | 23,000 LY | 6 days per person* |

*averaged figure in the target population (1.5 million Finns) over the period of study (10-40 years)

**Estimated effects of pharmacological treatment 20% more than in the base case**

| | | blood pressure group classification | | | |
|---|---|---|---|---|---|
| **males** | | BPG 0 | BPG 1 | BPG 2 | BPG 3 |
| age group | 70–74 | €217 000/LY | €92 000/LY | ACCG dominates | ACCG dominated |
| | | - €5m | - €1m | - €6m | €2m |
| | | -23 LY | -13 LY | 16 LY | -58 LY |
| | 65–69 | €103 000/LY | €1 078 000/LY | ACCG dominates | €1 000/LY |
| | | - €6m | - €4m | - €8m | €1m |
| | | -56 LY | -4 LY | 371 LY | 552 LY |
| | 60–64 | €42 000/LY | €14 000/LY | ACCG dominates | €2 000/LY |
| | | - €9m | - €9m | - €17m | €1m |
| | | -223 LY | -617 LY | 263 LY | 665 LY |
| | 55–59 | €15,000/LY | ACCG dominates | ACCG dominates | €1,000/LY |
| | | - €11m | - €4m | - €15m | €5m |
| | | -746 LY | 1 052 LY | 2 251 LY | 4 401 LY |
| | 50–54 | €42,000/LY | ACCG dominates | ACCG dominates | €3,000/LY |
| | | - €24m | - €9m | - €29m | €17m |
| | | -572 LY | 2 011 LY | 3,586 LY | 5,705 LY |
| | 45–49 | €34,000/LY | ACCG dominates | ACCG dominates | €5,000/LY |
| | | - €29m | - €12m | - €26m | €15m |
| | | -841 LY | 2 715 LY | 869 LY | 3,027 LY |
| | 40–44 | €42,000/LY | ACCG dominates | ACCG dominates | €3,000/LY |
| | | - €38m | - €9m | - €19m | €7m |
| | | -904 LY | 1 390 LY | 220 LY | 2,248 LY |
| **females** | | BPG 0 | BPG 1 | BPG 2 | BPG 3 |
| age group | 70–74 | ACCG dominates | ACCG dominates | ACCG dominates | €11,000/LY |
| | | - €3m | - €5m | - €11m | €6m |
| | | 4 LY | 14 LY | 349 LY | 550 LY |
| | 65–69 | €95,000/LY | €45,000/LY | ACCG dominates | €3,000/LY |
| | | - €3m | - €12m | - €12m | €4m |
| | | -36 LY | -264 LY | 788 LY | 1,622 LY |
| | 60–64 | €50,000/LY | €22,000/LY | €40 000/LY | €2,000/LY |
| | | - €10m | - €14m | - €18m | €6m |
| | | -193 LY | -624 LY | -460 LY | 3,329 LY |
| | 55–59 | €30 000/LY | ACCG dominates | ACCG dominates | €1,000/LY |
| | | - €13m | - €5m | - €23m | €15m |
| | | -435 LY | 749 LY | 2 540 LY | 12,484 LY |
| | 50–54 | €42 000/LY | ACCG dominates | ACCG dominates | €2,000/LY |
| | | - €35m | - €9m | - €29m | €15m |
| | | -843 LY | 2,012 LY | 15 LY | 8,881 LY |
| | 45–49 | €69 000/LY | ACCG dominates | €24,000/LY | €2,000/LY |
| | | - €48m | - €11m | - €28m | €8m |
| | | -698 LY | 2,810 LY | -1 163 LY | 3 762 LY |
| | 40–44 | €52 000/LY | ACCG dominates | €19 000/LY | €2,000/LY |
| | | - €60m | - €8m | - €18m | €5m |
| | | -1,137 LY | 1,910 LY | -939 LY | 2 422 LY |

| | | | |
|---|---|---|---|
| aggregated incremental cost-effectiveness ratio | ACCG dominates | | |
| aggregated incremental costs | - €559m | -400 | euros per person* |
| aggregated incremental effects | 65,000 LY | 16 | days per person* |

*averaged figure in the target population (1.5 million Finns) over the period of study (10-40 years)

**Estimated effect of ageing on BP is 50% of that in the base case**

blood pressure group classification

**males**

| age group | | BPG 0 | BPG 1 | BPG 2 | BPG 3 |
|---|---|---|---|---|---|
| 70–74 | | €1 410 000/LY | €334 000/LY | ACCG dominates | ACCG dominated |
| | | - €5m | - €1m | - €5m | €2m |
| | | -4 LY | -4 LY | 22 LY | -57 LY |
| 65–69 | | €102 000/LY | €133 000/LY | ACCG dominates | €3 000/LY |
| | | - €6m | - €4m | - €7m | €1m |
| | | -57 LY | -29 LY | 328 LY | 485 LY |
| 60–64 | | €85 000/LY | €12 000/LY | ACCG dominates | €2 000/LY |
| | | - €10m | - €8m | - €17m | €1m |
| | | -113 LY | -696 LY | 136 LY | 699 LY |
| 55–59 | | €24,000/LY | ACCG dominates | ACCG dominates | €2,000/LY |
| | | - €12m | - €4m | - €12m | €7m |
| | | -499 LY | 794 LY | 931 LY | 3 790 LY |
| 50–54 | | €98,000/LY | ACCG dominates | ACCG dominates | €3,000/LY |
| | | - €26m | - €9m | - €26m | €18m |
| | | -263 LY | 1 838 LY | 1,982 LY | 5,704 LY |
| 45–49 | | €59,000/LY | ACCG dominates | ACCG dominates | €5,000/LY |
| | | - €32m | - €13m | - €27m | €16m |
| | | -539 LY | 2 634 LY | 762 LY | 2,952 LY |
| 40–44 | | €117,000/LY | ACCG dominates | ACCG dominates | €3,000/LY |
| | | - €43m | - €8m | - €21m | €8m |
| | | -367 LY | 618 LY | 441 LY | 2,393 LY |

**females**

| age group | | BPG 0 | BPG 1 | BPG 2 | BPG 3 |
|---|---|---|---|---|---|
| 70–74 | | ACCG dominates | ACCG dominates | ACCG dominates | €13,000/LY |
| | | - €3m | - €5m | - €11m | €7m |
| | | 1 LY | 12 LY | 141 LY | 503 LY |
| 65–69 | | €168,000/LY | €44,000/LY | ACCG dominates | €3,000/LY |
| | | - €4m | - €12m | - €12m | €5m |
| | | -22 LY | -272 LY | 456 LY | 1,655 LY |
| 60–64 | | €283,000/LY | €22,000/LY | €62 000/LY | €2,000/LY |
| | | - €11m | - €14m | - €19m | €6m |
| | | -38 LY | -631 LY | -311 LY | 3,751 LY |
| 55–59 | | €96 000/LY | ACCG dominates | ACCG dominates | €1,000/LY |
| | | - €15m | - €6m | - €25m | €17m |
| | | -158 LY | 462 LY | 1 078 LY | 11,488 LY |
| 50–54 | | €79 000/LY | ACCG dominates | ACCG dominates | €2,000/LY |
| | | - €39m | - €11m | - €31m | €17m |
| | | -500 LY | ,469 LY | 1 149 LY | 8,567 LY |
| 45–49 | | €199 000/LY | ACCG dominates | €82,000/LY | €3,000/LY |
| | | - €53m | - €13m | - €29m | €10m |
| | | -269 LY | 1,705 LY | -357 LY | 3 426 LY |
| 40–44 | | €179 000/LY | ACCG dominates | €87 000/LY | €3,000/LY |
| | | - €67m | - €9m | - €19m | €6m |
| | | -,373 LY | 1,083 LY | -214 LY | 2 288 LY |

| | | |
|---|---|---|
| aggregated incremental cost-effectiveness ratio | ACCG dominates | |
| aggregated incremental costs | - €583m | -400 euros per person* |
| aggregated incremental effects | 59,000 LY | 14 days per person* |

*averaged figure in the target population (1.5 million Finns) over the period of study (10-40 years)

**adjustment for health-related quality of life for resultant morbidity and for BP group status**

blood pressure group classification

**males** (age group)

| | BPG 0 | BPG 1 | BPG 2 | BPG 3 |
|---|---|---|---|---|
| 70–74 | ACCG dominates | ACCG dominates | ACCG dominates | €131 000/LY |
| | - €5m | - €1m | - €5m | €2m |
| | 10 LY | 6 LY | 76 LY | 19 LY |
| 65–69 | €153 000/LY | €76 000/LY | ACCG dominates | €5 000/LY |
| | - €6m | - €4m | - €6m | €3m |
| | -37 LY | -46 LY | 382 LY | 529 LY |
| 60–64 | €52 000/LY | €12 000/LY | ACCG dominates | €2 000/LY |
| | - €9m | - €8m | - €16m | €2m |
| | -181 LY | -655 LY | 159 LY | 761 LY |
| 55–59 | €18,000/LY | ACCG dominates | ACCG dominates | €2,000/LY |
| | - €11m | - €3m | - €12m | €9m |
| | -617 LY | 352 LY | 1 972 LY | 3 673 LY |
| 50–54 | €53,000/LY | ACCG dominates | ACCG dominates | €5,000/LY |
| | - €24m | - €7m | - €25m | €23m |
| | -445 LY | 1 637 LY | 3,820 LY | 5,125 LY |
| 45–49 | €42,000/LY | ACCG dominates | ACCG dominates | €8,000/LY |
| | - €29m | - €10m | - €25m | €20m |
| | -685 LY | 2 819 LY | 1 467 LY | 2,478 LY |
| 40–44 | €50,000/LY | ACCG dominates | ACCG dominates | €4,000/LY |
| | - €38m | - €7m | - €18m | €11m |
| | -763 LY | 1 838 LY | 376 LY | 2,407 LY |

**females** (age group)

| | BPG 0 | BPG 1 | BPG 2 | BPG 3 |
|---|---|---|---|---|
| 70–74 | €742 000/LY | €144 000/LY | €172 000/LY | €13,000/LY |
| | - €3m | - €5m | - €11m | €7m |
| | -4 LY | -32 LY | -66 LY | 546 LY |
| 65–69 | €59,000/LY | €31,000/LY | €15,000/LY | €5,000/LY |
| | - €3m | - €12m | - €12m | €6m |
| | -57 LY | -385 LY | -754 LY | 1,228 LY |
| 60–64 | €39,000/LY | €15,000/LY | €37 000/LY | €3,000/LY |
| | - €10m | - €14m | - €19m | €7m |
| | -245 LY | -924 LY | -515 LY | 2,366 LY |
| 55–59 | €26 000/LY | ACCG dominates | ACCG dominates | €2,000/LY |
| | - €13m | - €5m | - €24m | €18m |
| | -497 LY | 1068 LY | 328 LY | 11,649 LY |
| 50–54 | €34 000/LY | ACCG dominates | €18,000/LY | €3,000/LY |
| | - €35m | - €9m | - €29m | €18m |
| | -1 032 LY | 1,825 LY | -1 658 LY | 6,808 LY |
| 45–49 | €57 000/LY | ACCG dominates | €11,000/LY | €3,000/LY |
| | - €48m | - €10m | - €28m | €10m |
| | -846 LY | 2,403 LY | -2 612 LY | 2 993 LY |
| 40–44 | €44 000/LY | ACCG dominates | €20 000/LY | €4,000/LY |
| | - €60m | - €7m | - €18m | €7m |
| | -1,366 LY | 2,449 LY | -886 LY | 1 735 LY |

| | | |
|---|---|---|
| aggregated incremental cost-effectiveness ratio | ACCG dominates | |
| aggregated incremental costs | - €498m | -300 euros per person* |
| aggregated incremental effects | 50,000 LY | 12 days per person* |

*averaged figure in the target population (1.5 million Finns) over the period of study (10-40 years)

**Lifestyle intervention costs 50% of those used in base case estimates**

blood pressure group classification

**males**

| age group | BPG 0 | BPG 1 | BPG 2 | BPG 3 |
|---|---|---|---|---|
| 70–74 | €220 000/LY | €91 000/LY | ACCG dominates | €/LY |
| | - €5m | - €1m | - €7m | €m |
| | -23 LY | -15 LY | 19 LY | -65 LY |
| 65–69 | €104 000/LY | €38 000/LY | ACCG dominates | ACCG dominates |
| | - €6m | - €4m | - €9m | - €2m |
| | -56 LY | -110 LY | 312 LY | 395 LY |
| 60–64 | €43 000/LY | €11 000/LY | ACCG dominates | ACCG dominates |
| | - €9m | - €9m | - €19m | - €2m |
| | -223 LY | -860 LY | 223 LY | 678 LY |
| 55–59 | €16,000/LY | ACCG dominates | ACCG dominates | €1,000/LY |
| | - €12m | - €5m | - €16m | €2m |
| | -746 LY | 146 LY | 1 847 LY | 3 420 LY |
| 50–54 | €44,000/LY | ACCG dominates | ACCG dominates | €2,000/LY |
| | - €25m | - €12m | - €32m | €9m |
| | -572 LY | 1 064 LY | 3,366 LY | 4,476 LY |
| 45–49 | €37,000/LY | ACCG dominates | ACCG dominates | €5,000/LY |
| | - €31m | - €16m | - €30m | €9m |
| | -841 LY | 1 705 LY | 1 171 LY | 2,040 LY |
| 40–44 | €45,000/LY | ACCG dominates | ACCG dominates | €2,000/LY |
| | - €41m | - €13m | - €22m | €4m |
| | -904 LY | 891 LY | 287 LY | 1,898 LY |

**females**

| age group | BPG 0 | BPG 1 | BPG 2 | BPG 3 |
|---|---|---|---|---|
| 70–74 | ACCG dominates | ACCG dominates | ACCG dominates | €2,000/LY |
| | - €3m | - €5m | - €12m | €1m |
| | 4 LY | 15 LY | 332 LY | 567 LY |
| 65–69 | €99,000/LY | €54,000/LY | €35,000/LY | €,/LY |
| | - €4m | - €13m | - €13m | €1m |
| | -36 LY | -247 LY | -359 LY | 1,327 LY |
| 60–64 | €52,000/LY | €22,000/LY | ACCG dominates | €,/LY |
| | - €10m | - €15m | - €20m | €1m |
| | -193 LY | -699 LY | 54 LY | 2,559 LY |
| 55–59 | €32 000/LY | ACCG dominates | ACCG dominates | €1,000/LY |
| | - €14m | - €8m | - €27m | €8m |
| | -435 LY | 574 LY | 1 387 LY | 12,756 LY |
| 50–54 | €45 000/LY | ACCG dominates | €62,000/LY | €1,000/LY |
| | - €38m | - €14m | - €33m | €7m |
| | -843 LY | 1,404 LY | -527 LY | 7,388 LY |
| 45–49 | €73 000/LY | ACCG dominates | €17,000/LY | €1,000/LY |
| | - €51m | - €15m | - €31m | €4m |
| | -698 LY | 1,892 LY | -1 804 LY | 3 191 LY |
| 40–44 | €56 000/LY | ACCG dominates | €50 000/LY | €1,000/LY |
| | - €64m | - €11m | - €20m | €3m |
| | -1,137 LY | 1,734 LY | -397 LY | 1 812 LY |

| | | |
|---|---|---|
| aggregated incremental cost-effectiveness ratio | ACCG dominates | |
| aggregated incremental costs | - €696m | -500 euros per person* |
| aggregated incremental effects | 49,000 LY | 12 days per person* |

*averaged figure in the target population (1.5 million Finns) over the period of study (10-40 years)

**Lifestyle intervention costs 150% of those used in base case estimates**

blood pressure group classification

**males**

| age group | BPG 0 | BPG 1 | BPG 2 | BPG 3 |
|---|---|---|---|---|
| 70–74 | €213 000/LY | €56 000/LY | ACCG dominates | ACCG dominated |
| | - €5m | - €1m | - €3m | €4m |
| | -23 LY | -15 LY | 19 LY | -65 LY |
| 65–69 | €101 000/LY | €26 000/LY | ACCG dominates | €12 000/LY |
| | - €6m | - €3m | - €4m | €5m |
| | -56 LY | -110 LY | 312 LY | 395 LY |
| 60–64 | €41 000/LY | €7 000/LY | ACCG dominates | €5 000/LY |
| | - €9m | - €6m | - €13m | €3m |
| | -223 LY | -860 LY | 223 LY | 678 LY |
| 55–59 | €14,000/LY | ACCG dominates | ACCG dominates | €3,000/LY |
| | - €11m | - €1m | - €8m | €11m |
| | -746 LY | 146 LY | 1 847 LY | 3 420 LY |
| 50–54 | €39,000/LY | ACCG dominates | ACCG dominates | €6,000/LY |
| | - €22m | - €3m | - €18m | €28m |
| | -572 LY | 1 064 LY | 3,366 LY | 4,476 LY |
| 45–49 | €32,000/LY | ACCG dominates | ACCG dominates | €11,000/LY |
| | - €27m | - €4m | - €17m | €23m |
| | -841 LY | 1 705 LY | 1 171 LY | 2,040 LY |
| 40–44 | €39,000/LY | ACCG dominates | ACCG dominates | €7,000/LY |
| | - €35m | - €2m | - €12m | €13m |
| | -904 LY | 891 LY | 287 LY | 1,898 LY |

**females**

| age group | BPG 0 | BPG 1 | BPG 2 | BPG 3 |
|---|---|---|---|---|
| 70–74 | ACCG dominates | ACCG dominates | ACCG dominates | €17,000/LY |
| | - €3m | - €4m | - €9m | €10m |
| | 4 LY | 15 LY | 332 LY | 567 LY |
| 65–69 | €91,000/LY | €42,000/LY | €26,000/LY | €6,000/LY |
| | - €3m | - €10m | - €9m | €8m |
| | -36 LY | -247 LY | -359 LY | 1,327 LY |
| 60–64 | €47,000/LY | €17,000/LY | ACCG dominates | €4,000/LY |
| | - €9m | - €12m | - €16m | €10m |
| | -193 LY | -699 LY | 54 LY | 2,559 LY |
| 55–59 | €28 000/LY | ACCG dominates | ACCG dominates | €2,000/LY |
| | - €12m | - €2m | - €19m | €22m |
| | -435 LY | 574 LY | 1 387 LY | 12,756 LY |
| 50–54 | €39 000/LY | ACCG dominates | €45,000/LY | €3,000/LY |
| | - €33m | - €4m | - €24m | €22m |
| | -843 LY | 1,404 LY | -527 LY | 7,388 LY |
| 45–49 | €65 000/LY | ACCG dominates | €13,000/LY | €4,000/LY |
| | - €45m | - €5m | - €23m | €12m |
| | -698 LY | 1,892 LY | -1 804 LY | 3 191 LY |
| 40–44 | €49 000/LY | ACCG dominates | €36 000/LY | €5,000/LY |
| | - €56m | - €3m | - €14m | €9m |
| | -1,137 LY | 1,734 LY | -397 LY | 1 812 LY |

| | | |
|---|---|---|
| aggregated incremental cost-effectiveness ratio | ACCG dominates | |
| aggregated incremental costs | - €347m | -200 euros per person* |
| aggregated incremental effects | 49,000 LY | 12 days per person* |

*averaged figure in the target population (1.5 million Finns) over the period of study (10-40 years)

**Lifestyle intervention costs 200% of those used in base case estimates**

blood pressure group classification

**males** (age group)

| | BPG 0 | BPG 1 | BPG 2 | BPG 3 |
|---|---|---|---|---|
| 70–74 | €210 000/LY | €39 000/LY | ACCG dominates | ACCG dominated |
| | - €5m | - €1m | - €2m | €5m |
| | -23 LY | -15 LY | 19 LY | -65 LY |
| 65–69 | €100 000/LY | €20 000/LY | ACCG dominates | €16 000/LY |
| | - €6m | - €2m | - €2m | €6m |
| | -56 LY | -110 LY | 312 LY | 395 LY |
| 60–64 | €41 000/LY | €6 000/LY | ACCG dominates | €7 000/LY |
| | - €9m | - €5m | - €10m | €5m |
| | -223 LY | -860 LY | 223 LY | 678 LY |
| 55–59 | €13,000/LY | €10 000/LY | ACCG dominates | €4,000/LY |
| | - €10m | €1m | - €3m | €14m |
| | -746 LY | 146 LY | 1 847 LY | 3 420 LY |
| 50–54 | €37,000/LY | €2 000/LY | ACCG dominates | €7,000/LY |
| | - €21m | €2m | - €10m | €33m |
| | -572 LY | 1 064 LY | 3,366 LY | 4,476 LY |
| 45–49 | €30,000/LY | €1 000/LY | ACCG dominates | €13,000/LY |
| | - €25m | €2m | - €10m | €26m |
| | -841 LY | 1 705 LY | 1 171 LY | 2,040 LY |
| 40–44 | €36,000/LY | €4 000/LY | ACCG dominates | €8,000/LY |
| | - €33m | €4m | - €7m | €15m |
| | -904 LY | 891 LY | 287 LY | 1,898 LY |

**females** (age group)

| | BPG 0 | BPG 1 | BPG 2 | BPG 3 |
|---|---|---|---|---|
| 70–74 | ACCG dominates | ACCG dominates | ACCG dominates | €22,000/LY |
| | - €3m | - €3m | - €7m | €13m |
| | 4 LY | 15 LY | 332 LY | 567 LY |
| 65–69 | €87,000/LY | €36,000/LY | €20,000/LY | €7,000/LY |
| | - €3m | - €9m | - €7m | €10m |
| | -36 LY | -247 LY | -359 LY | 1,327 LY |
| 60–64 | €45,000/LY | €15,000/LY | ACCG dominates | €5,000/LY |
| | - €9m | - €10m | - €13m | €13m |
| | -193 LY | -699 LY | 54 LY | 2,559 LY |
| 55–59 | €26 000/LY | €/LY | ACCG dominates | €2,000/LY |
| | - €11m | €m | - €14m | €25m |
| | -435 LY | 574 LY | 1 387 LY | 12,756 LY |
| 50–54 | €36 000/LY | €1 000/LY | €35,000/LY | €3,000/LY |
| | - €30m | €1m | - €18m | €25m |
| | -843 LY | 1,404 LY | -527 LY | 7,388 LY |
| 45–49 | €60 000/LY | ACCG dominates | €10,000/LY | €4,000/LY |
| | - €42m | - €1m | - €17m | €14m |
| | -698 LY | 1,892 LY | -1 804 LY | 3 191 LY |
| 40–44 | €45 000/LY | €1 000/LY | €28 000/LY | €5,000/LY |
| | - €52m | €1m | - €11m | €10m |
| | -1,137 LY | 1,734 LY | -397 LY | 1 812 LY |

| | | |
|---|---|---|
| aggregated incremental cost-effectiveness ratio | ACCG dominates | |
| aggregated incremental costs | - €196m | -100 euros per person* |
| aggregated incremental effects | 49,000 LY | 12 days per person* |

**Lifestyle intervention costs 400% of those used in base case estimates**

blood pressure group classification

**males**

| age group | BPG 0 | BPG 1 | BPG 2 | BPG 3 |
|---|---|---|---|---|
| 70–74 | €196 000/LY | ACCG dominated | €229,000/LY | ACCG dominated |
| | - €4m | €m | €4m | €9m |
| | -23 LY | -15 LY | 19 LY | -65 LY |
| 65–69 | €94 000/LY | ACCG dominated | €18 000/LY | €36 000/LY |
| | - €5m | €m | €6m | €14m |
| | -56 LY | -110 LY | 312 LY | 395 LY |
| 60–64 | €38 000/LY | ACCG dominated | €6 000/LY | €16 000/LY |
| | - €8m | €1m | €1m | €11m |
| | -223 LY | -860 LY | 223 LY | 678 LY |
| 55–59 | €10,000/LY | €70 000/LY | €8 000/LY | €7,000/LY |
| | - €7m | €10m | €15m | €23m |
| | -746 LY | 146 LY | 1 847 LY | 3 420 LY |
| 50–54 | €28,000/LY | €19 000/LY | €6 000/LY | €11,000/LY |
| | - €16m | €20m | €22m | €51m |
| | -572 LY | 1 064 LY | 3,366 LY | 4,476 LY |
| 45–49 | €21,000/LY | €15 000/LY | €17 000/LY | €19,000/LY |
| | - €18m | €26m | €20m | €39m |
| | -841 LY | 1 705 LY | 1 171 LY | 2,040 LY |
| 40–44 | €25,000/LY | €28 000/LY | €53 000/LY | €13,000/LY |
| | - €23m | €25m | €15m | €24m |
| | -904 LY | 891 LY | 287 LY | 1,898 LY |

**females**

| age group | BPG 0 | BPG 1 | BPG 2 | BPG 3 |
|---|---|---|---|---|
| 70–74 | ACCG dominates | ACCG dominates | €6 000/LY | €41,000/LY |
| | - €2m | - €1m | €2m | €23m |
| | 4 LY | 15 LY | 332 LY | 567 LY |
| 65–69 | €71,000/LY | €12,000/LY | ACCG dominated | €14,000/LY |
| | - €3m | - €3m | €1m | €18m |
| | -36 LY | -247 LY | -359 LY | 1,327 LY |
| 60–64 | €36,000/LY | €6,000/LY | €/LY | €9,000/LY |
| | - €7m | - €4m | €m | €23m |
| | -193 LY | -699 LY | 54 LY | 2,559 LY |
| 55–59 | €17 000/LY | €18 000/LY | €3,000/LY | €3,000/LY |
| | - €8m | €11m | €5m | €39m |
| | -435 LY | 574 LY | 1 387 LY | 12,756 LY |
| 50–54 | €24 000/LY | €14 000/LY | ACCG dominated | €5,000/LY |
| | - €20m | €20m | €4m | €40m |
| | -843 LY | 1,404 LY | -527 LY | 7,388 LY |
| 45–49 | €43 000/LY | €9 000/LY | ACCG dominated | €7,000/LY |
| | - €30m | €18m | €3m | €22m |
| | -698 LY | 1,892 LY | -1 804 LY | 3 191 LY |
| 40–44 | €31 000/LY | €10 000/LY | ACCG dominated | €9,000/LY |
| | - €36m | €17m | €2m | €15m |
| | -1,137 LY | 1,734 LY | -397 LY | 1 812 LY |

| | | |
|---|---|---|
| aggregated incremental cost-effectiveness ratio | €8 000/LY | |
| aggregated incremental costs | €407m | 300 euros per person* |
| aggregated incremental effects | 49,000 LY | 12 days per person* |

*averaged figure in the target population (1.5 million Finns) over the period of study (10-40 years)

# Sensitivity analysis



**medication costs only from the SII perspective (VAT deducted)**

blood pressure group classification

## males

| age group | BPG 0 | BPG 1 | BPG 2 | BPG 3 |
|---|---|---|---|---|
| 70–74 | €139 000/LY | €34 000/LY | ACCG dominates | ACCG dominated |
| | - €3m | - €1m | - €2m | €3m |
| | -23 LY | -15 LY | 19 LY | -65 LY |
| 65–69 | €67 000/LY | €16 000/LY | ACCG dominates | €9 000/LY |
| | - €4m | - €2m | - €3m | €4m |
| | -56 LY | -110 LY | 312 LY | 395 LY |
| 60–64 | €27 000/LY | €4 000/LY | ACCG dominates | €4 000/LY |
| | - €6m | - €4m | - €8m | €3m |
| | -223 LY | -860 LY | 223 LY | 678 LY |
| 55–59 | €9,000/LY | ACCG dominates | ACCG dominates | €2,000/LY |
| | - €7m | €m | - €5m | €7m |
| | -746 LY | 146 LY | 1 847 LY | 3 420 LY |
| 50–54 | €25,000/LY | ACCG dominates | ACCG dominates | €4,000/LY |
| | - €14m | - €1m | - €11m | €19m |
| | -572 LY | 1 064 LY | 3,366 LY | 4,476 LY |
| 45–49 | €20,000/LY | ACCG dominates | ACCG dominates | €8,000/LY |
| | - €17m | - €3m | - €9m | €16m |
| | -841 LY | 1 705 LY | 1 171 LY | 2,040 LY |
| 40–44 | €25,000/LY | ACCG dominates | ACCG dominates | €4,000/LY |
| | - €22m | €m | - €6m | €8m |
| | -904 LY | 891 LY | 287 LY | 1,898 LY |

## females

| age group | BPG 0 | BPG 1 | BPG 2 | BPG 3 |
|---|---|---|---|---|
| 70–74 | ACCG dominates | ACCG dominates | ACCG dominates | €13,000/LY |
| | - €2m | - €3m | - €7m | €8m |
| | 4 LY | 15 LY | 332 LY | 567 LY |
| 65–69 | €66,000/LY | €31,000/LY | €19,000/LY | €5,000/LY |
| | - €2m | - €8m | - €7m | €6m |
| | -36 LY | -247 LY | -359 LY | 1,327 LY |
| 60–64 | €34,000/LY | €12,000/LY | ACCG dominates | €3,000/LY |
| | - €7m | - €9m | - €11m | €8m |
| | -193 LY | -699 LY | 54 LY | 2,559 LY |
| 55–59 | €20 000/LY | ACCG dominates | ACCG dominates | €1,000/LY |
| | - €9m | - €2m | - €13m | €17m |
| | -435 LY | 574 LY | 1 387 LY | 12,756 LY |
| 50–54 | €28 000/LY | ACCG dominates | €31,000/LY | €2,000/LY |
| | - €24m | - €3m | - €16m | €17m |
| | -843 LY | 1,404 LY | -527 LY | 7,388 LY |
| 45–49 | €47 000/LY | ACCG dominates | €9,000/LY | €3,000/LY |
| | - €33m | - €4m | - €16m | €10m |
| | -698 LY | 1,892 LY | -1 804 LY | 3 191 LY |
| 40–44 | €35 000/LY | ACCG dominates | €25 000/LY | €4,000/LY |
| | - €40m | - €2m | - €10m | €7m |
| | -1,137 LY | 1,734 LY | -397 LY | 1 812 LY |

| | | |
|---|---|---|
| aggregated incremental cost-effectiveness ratio | ACCG dominates | |
| aggregated incremental costs | - €223m | -100 euros per person* |
| aggregated incremental effects | 49,000 LY | 12 days per person* |

*averaged figure in the target population (1.5 million Finns) over the period of study (10-40 years)

**medication costs inclusive of user charges (estimated effect of generic substitution on prices)**

blood pressure group classification

**males**

| age group | BPG 0 | BPG 1 | BPG 2 | BPG 3 |
|---|---|---|---|---|
| 70–74 | €169 000/LY | €49 000/LY | ACCG dominates | ACCG dominated |
| | - €4m | - €1m | - €3m | €2m |
| | -23 LY | -15 LY | 19 LY | -65 LY |
| 65–69 | €80 000/LY | €22 000/LY | ACCG dominates | €8 000/LY |
| | - €4m | - €2m | - €4m | €3m |
| | -56 LY | -110 LY | 312 LY | 395 LY |
| 60–64 | €32 000/LY | €6 000/LY | ACCG dominates | €3 000/LY |
| | - €7m | - €5m | - €11m | €2m |
| | -223 LY | -860 LY | 223 LY | 678 LY |
| 55–59 | €11,000/LY | ACCG dominates | ACCG dominates | €2,000/LY |
| | - €8m | - €1m | - €7m | €8m |
| | -746 LY | 146 LY | 1 847 LY | 3 420 LY |
| 50–54 | €30,000/LY | ACCG dominates | ACCG dominates | €5,000/LY |
| | - €17m | - €3m | - €16m | €20m |
| | -572 LY | 1 064 LY | 3,366 LY | 4,476 LY |
| 45–49 | €25,000/LY | ACCG dominates | ACCG dominates | €9,000/LY |
| | - €21m | - €5m | - €14m | €17m |
| | -841 LY | 1 705 LY | 1 171 LY | 2,040 LY |
| 40–44 | €30,000/LY | ACCG dominates | ACCG dominates | €5,000/LY |
| | - €27m | - €3m | - €10m | €9m |
| | -904 LY | 891 LY | 287 LY | 1,898 LY |

**females**

| age group | BPG 0 | BPG 1 | BPG 2 | BPG 3 |
|---|---|---|---|---|
| 70–74 | ACCG dominates | ACCG dominates | ACCG dominates | €12,000/LY |
| | - €2m | - €3m | - €8m | €7m |
| | 4 LY | 15 LY | 332 LY | 567 LY |
| 65–69 | €74,000/LY | €36,000/LY | €23,000/LY | €4,000/LY |
| | - €3m | - €9m | - €8m | €5m |
| | -36 LY | -247 LY | -359 LY | 1,327 LY |
| 60–64 | €38,000/LY | €14,000/LY | ACCG dominates | €3,000/LY |
| | - €7m | - €10m | - €13m | €7m |
| | -193 LY | -699 LY | 54 LY | 2,559 LY |
| 55–59 | €22 000/LY | ACCG dominates | ACCG dominates | €1,000/LY |
| | - €10m | - €3m | - €16m | €17m |
| | -435 LY | 574 LY | 1 387 LY | 12,756 LY |
| 50–54 | €31 000/LY | ACCG dominates | €37,000/LY | €2,000/LY |
| | - €26m | - €4m | - €19m | €17m |
| | -843 LY | 1,404 LY | -527 LY | 7,388 LY |
| 45–49 | €52 000/LY | ACCG dominates | €10,000/LY | €3,000/LY |
| | - €36m | - €5m | - €18m | €9m |
| | -698 LY | 1,892 LY | -1 804 LY | 3 191 LY |
| 40–44 | €40 000/LY | ACCG dominates | €30 000/LY | €4,000/LY |
| | - €45m | - €4m | - €12m | €6m |
| | -1,137 LY | 1,734 LY | -397 LY | 1 812 LY |

| | | |
|---|---|---|
| aggregated incremental cost-effectiveness ratio | ACCG dominates | |
| aggregated incremental costs | - €306m | -200 euros per person* |
| aggregated incremental effects | 49,000 LY | 12 days per person* |

*averaged figure in the target population (1.5 million Finns) over the period of study (10-40 years)

**medication costs only from the SII perspective (estimated effect of generic substitution on prices)**

blood pressure group classification

**males**

| age group | BPG 0 | BPG 1 | BPG 2 | BPG 3 |
|---|---|---|---|---|
| 70–74 | €107 000/LY | €17 000/LY | ACCG dominates | ACCG dominated |
| | - €2m | €m | - €1m | €3m |
| | -23 LY | -15 LY | 19 LY | -65 LY |
| 65–69 | €51 000/LY | €9 000/LY | ACCG dominates | €9 000/LY |
| | - €3m | - €1m | - €1m | €4m |
| | -56 LY | -110 LY | 312 LY | 395 LY |
| 60–64 | €20 000/LY | €2 000/LY | ACCG dominates | €4 000/LY |
| | - €5m | - €2m | - €5m | €3m |
| | -223 LY | -860 LY | 223 LY | 678 LY |
| 55–59 | €6,000/LY | €7 000/LY | ACCG dominates | €2,000/LY |
| | - €4m | €1m | - €1m | €6m |
| | -746 LY | 146 LY | 1 847 LY | 3 420 LY |
| 50–54 | €17,000/LY | €1 000/LY | ACCG dominates | €4,000/LY |
| | - €10m | €1m | - €4m | €17m |
| | -572 LY | 1 064 LY | 3,366 LY | 4,476 LY |
| 45–49 | €14,000/LY | €/LY | ACCG dominates | €7,000/LY |
| | - €11m | €1m | - €2m | €15m |
| | -841 LY | 1 705 LY | 1 171 LY | 2,040 LY |
| 40–44 | €16,000/LY | €3 000/LY | ACCG dominates | €4,000/LY |
| | - €15m | €3m | - €1m | €7m |
| | -904 LY | 891 LY | 287 LY | 1,898 LY |

**females**

| age group | BPG 0 | BPG 1 | BPG 2 | BPG 3 |
|---|---|---|---|---|
| 70–74 | ACCG dominates | ACCG dominates | ACCG dominates | €13,000/LY |
| | - €2m | - €2m | - €4m | €7m |
| | 4 LY | 15 LY | 332 LY | 567 LY |
| 65–69 | €51,000/LY | €22,000/LY | €11,000/LY | €4,000/LY |
| | - €2m | - €5m | - €4m | €6m |
| | -36 LY | -247 LY | -359 LY | 1,327 LY |
| 60–64 | €26,000/LY | €9,000/LY | ACCG dominates | €3,000/LY |
| | - €5m | - €6m | - €7m | €8m |
| | -193 LY | -699 LY | 54 LY | 2,559 LY |
| 55–59 | €14 000/LY | ACCG dominates | ACCG dominates | €1,000/LY |
| | - €6m | €m | - €7m | €16m |
| | -435 LY | 574 LY | 1 387 LY | 12,756 LY |
| 50–54 | €20 000/LY | €/LY | €17,000/LY | €2,000/LY |
| | - €17m | €m | - €9m | €16m |
| | -843 LY | 1,404 LY | -527 LY | 7,388 LY |
| 45–49 | €34 000/LY | ACCG dominates | €5,000/LY | €3,000/LY |
| | - €24m | - €1m | - €9m | €9m |
| | -698 LY | 1,892 LY | -1 804 LY | 3 191 LY |
| 40–44 | €26 000/LY | €/LY | €14 000/LY | €3,000/LY |
| | - €30m | €m | - €6m | €6m |
| | -1,137 LY | 1,734 LY | -397 LY | 1 812 LY |

| | | | |
|---|---|---|---|
| aggregated incremental cost-effectiveness ratio | ACCG dominates | | |
| aggregated incremental costs | - €85m | -100 | euros per person* |
| aggregated incremental effects | 49,000 LY | 12 | days per person* |

*averaged figure in the target population (1.5 million Finns) over the period of study (10-40 years)

**medication costs inclusive of user charges (VAT not deducted)**

blood pressure group classification

**males**

| age group | BPG 0 | BPG 1 | BPG 2 | BPG 3 |
|---|---|---|---|---|
| 70–74 | €247 000/LY | €89 000/LY | ACCG dominates | ACCG dominated |
| | - €6m | - €1m | - €6m | €2m |
| | -23 LY | -15 LY | 19 LY | -65 LY |
| 65–69 | €117 000/LY | €38 000/LY | ACCG dominates | €6 000/LY |
| | - €7m | - €4m | - €8m | €2m |
| | -56 LY | -110 LY | 312 LY | 395 LY |
| 60–64 | €48 000/LY | €11 000/LY | ACCG dominates | €2 000/LY |
| | - €11m | - €10m | - €19m | €2m |
| | -223 LY | -860 LY | 223 LY | 678 LY |
| 55–59 | €18,000/LY | ACCG dominates | ACCG dominates | €3,000/LY |
| | - €13m | - €4m | - €15m | €10m |
| | -746 LY | 146 LY | 1 847 LY | 3 420 LY |
| 50–54 | €48,000/LY | ACCG dominates | ACCG dominates | €6,000/LY |
| | - €27m | - €9m | - €31m | €25m |
| | -572 LY | 1 064 LY | 3,366 LY | 4,476 LY |
| 45–49 | €40,000/LY | ACCG dominates | ACCG dominates | €10,000/LY |
| | - €34m | - €13m | - €31m | €21m |
| | -841 LY | 1 705 LY | 1 171 LY | 2,040 LY |
| 40–44 | €49,000/LY | ACCG dominates | ACCG dominates | €6,000/LY |
| | - €44m | - €10m | - €23m | €11m |
| | -904 LY | 891 LY | 287 LY | 1,898 LY |

**females**

| age group | BPG 0 | BPG 1 | BPG 2 | BPG 3 |
|---|---|---|---|---|
| 70–74 | ACCG dominates | ACCG dominates | ACCG dominates | €12,000/LY |
| | - €3m | - €5m | - €14m | €7m |
| | 4 LY | 15 LY | 332 LY | 567 LY |
| 65–69 | €109,000/LY | €57,000/LY | €39,000/LY | €4,000/LY |
| | - €4m | - €14m | - €14m | €5m |
| | -36 LY | -247 LY | -359 LY | 1,327 LY |
| 60–64 | €57,000/LY | €23,000/LY | ACCG dominates | €3,000/LY |
| | - €11m | - €16m | - €23m | €7m |
| | -193 LY | -699 LY | 54 LY | 2,559 LY |
| 55–59 | €35 000/LY | ACCG dominates | ACCG dominates | €1,000/LY |
| | - €15m | - €7m | - €29m | €19m |
| | -435 LY | 574 LY | 1 387 LY | 12,756 LY |
| 50–54 | €48 000/LY | ACCG dominates | €67,000/LY | €3,000/LY |
| | - €41m | - €12m | - €35m | €19m |
| | -843 LY | 1,404 LY | -527 LY | 7,388 LY |
| 45–49 | €80 000/LY | ACCG dominates | €19,000/LY | €3,000/LY |
| | - €56m | - €13m | - €34m | €10m |
| | -698 LY | 1,892 LY | -1 804 LY | 3 191 LY |
| 40–44 | €61 000/LY | ACCG dominates | €54 000/LY | €4,000/LY |
| | - €69m | - €9m | - €21m | €7m |
| | -1,137 LY | 1,734 LY | -397 LY | 1 812 LY |

| | | |
|---|---|---|
| aggregated incremental cost-effectiveness ratio | ACCG dominates | |
| aggregated incremental costs | - €621m | -400 euros per person* |
| aggregated incremental effects | 49,000 LY | 12 days per person* |

*averaged figure in the target population (1.5 million Finns) over the period of study (10-40 years)

**medication costs inclusive of user charges (VAT deducted) - range of medications used identical for ACCG and PCP**

blood pressure group classification

**males**

| age group | | BPG 0 | BPG 1 | BPG 2 | BPG 3 |
|---|---|---|---|---|---|
| 70–74 | | €247 000/LY | €89 000/LY | ACCG dominates | ACCG dominated |
| | | - €6m | - €1m | - €4m | €5m |
| | | -23 LY | -15 LY | 19 LY | -65 LY |
| 65–69 | | €117 000/LY | €38 000/LY | ACCG dominates | €19 000/LY |
| | | - €7m | - €4m | - €4m | €8m |
| | | -56 LY | -110 LY | 312 LY | 395 LY |
| 60–64 | | €48 000/LY | €11 000/LY | ACCG dominates | €9 000/LY |
| | | - €11m | - €10m | - €16m | €6m |
| | | -223 LY | -860 LY | 223 LY | 678 LY |
| 55–59 | | €18,000/LY | ACCG dominates | ACCG dominates | €5,000/LY |
| | | - €13m | - €4m | - €12m | €18m |
| | | -746 LY | 146 LY | 1 847 LY | 3 420 LY |
| 50–54 | | €48,000/LY | ACCG dominates | ACCG dominates | €9,000/LY |
| | | - €27m | - €9m | - €28m | €42m |
| | | -572 LY | 1 064 LY | 3,366 LY | 4,476 LY |
| 45–49 | | €40,000/LY | ACCG dominates | ACCG dominates | €16,000/LY |
| | | - €34m | - €13m | - €29m | €33m |
| | | -841 LY | 1 705 LY | 1 171 LY | 2,040 LY |
| 40–44 | | €49,000/LY | ACCG dominates | ACCG dominates | €10,000/LY |
| | | - €44m | - €10m | - €22m | €20m |
| | | -904 LY | 891 LY | 287 LY | 1,898 LY |

**females**

| age group | | BPG 0 | BPG 1 | BPG 2 | BPG 3 |
|---|---|---|---|---|---|
| 70–74 | | ACCG dominates | ACCG dominates | ACCG dominates | €25,000/LY |
| | | - €3m | - €5m | - €13m | €14m |
| | | 4 LY | 15 LY | 332 LY | 567 LY |
| 65–69 | | €109,000/LY | €57,000/LY | €38,000/LY | €9,000/LY |
| | | - €4m | - €14m | - €14m | €11m |
| | | -36 LY | -247 LY | -359 LY | 1,327 LY |
| 60–64 | | €57,000/LY | €23,000/LY | ACCG dominates | €6,000/LY |
| | | - €11m | - €16m | - €23m | €15m |
| | | -193 LY | -699 LY | 54 LY | 2,559 LY |
| 55–59 | | €35 000/LY | ACCG dominates | ACCG dominates | €2,000/LY |
| | | - €15m | - €7m | - €29m | €31m |
| | | -435 LY | 574 LY | 1 387 LY | 12,756 LY |
| 50–54 | | €48 000/LY | ACCG dominates | €67,000/LY | €4,000/LY |
| | | - €41m | - €12m | - €35m | €32m |
| | | -843 LY | 1,404 LY | -527 LY | 7,388 LY |
| 45–49 | | €80 000/LY | ACCG dominates | €19,000/LY | €6,000/LY |
| | | - €56m | - €13m | - €34m | €18m |
| | | -698 LY | 1,892 LY | -1 804 LY | 3 191 LY |
| 40–44 | | €61 000/LY | ACCG dominates | €54 000/LY | €7,000/LY |
| | | - €69m | - €9m | - €21m | €13m |
| | | -1,137 LY | 1,734 LY | -397 LY | 1 812 LY |

| | | | |
|---|---|---|---|
| aggregated incremental cost-effectiveness ratio | ACCG dominates | | |
| aggregated incremental costs | - €485m | -300 | euros per person* |
| aggregated incremental effects | 49,000 LY | 12 | days per person* |

*averaged figure in the target population (1.5 million Finns) over the period of study (10-40 years)

**morbidity costs: Initial cost of stroke 3,000€, yearly cost 3,000€. Initial cost of CHD 1,000€, yearly cost 1,000€.**

blood pressure group classification

**males**

| age group | BPG 0 | BPG 1 | BPG 2 | BPG 3 |
|---|---|---|---|---|
| 70–74 | €215 000/LY | €72 000/LY | ACCG dominates | ACCG dominated |
| | - €5m | - €1m | - €5m | €2m |
| | -23 LY | -15 LY | 19 LY | -65 LY |
| 65–69 | €102 000/LY | €32 000/LY | ACCG dominates | €6 000/LY |
| | - €6m | - €3m | - €6m | €2m |
| | -56 LY | -110 LY | 312 LY | 395 LY |
| 60–64 | €42 000/LY | €9 000/LY | ACCG dominates | €2 000/LY |
| | - €9m | - €8m | - €16m | €2m |
| | -223 LY | -860 LY | 223 LY | 678 LY |
| 55–59 | €15,000/LY | ACCG dominates | ACCG dominates | €2,000/LY |
| | - €11m | - €3m | - €13m | €8m |
| | -746 LY | 146 LY | 1 847 LY | 3 420 LY |
| 50–54 | €41,000/LY | ACCG dominates | ACCG dominates | €5,000/LY |
| | - €24m | - €7m | - €27m | €22m |
| | -572 LY | 1 064 LY | 3,366 LY | 4,476 LY |
| 45–49 | €34,000/LY | ACCG dominates | ACCG dominates | €9,000/LY |
| | - €29m | - €11m | - €25m | €19m |
| | -841 LY | 1 705 LY | 1 171 LY | 2,040 LY |
| 40–44 | €42,000/LY | ACCG dominates | ACCG dominates | €5,000/LY |
| | - €38m | - €7m | - €18m | €10m |
| | -904 LY | 891 LY | 287 LY | 1,898 LY |

**females**

| age group | BPG 0 | BPG 1 | BPG 2 | BPG 3 |
|---|---|---|---|---|
| 70–74 | ACCG dominates | ACCG dominates | ACCG dominates | €12,000/LY |
| | - €3m | - €5m | - €11m | €7m |
| | 4 LY | 15 LY | 332 LY | 567 LY |
| 65–69 | €94,000/LY | €47,000/LY | €32,000/LY | €4,000/LY |
| | - €3m | - €12m | - €11m | €6m |
| | -36 LY | -247 LY | -359 LY | 1,327 LY |
| 60–64 | €49,000/LY | €19,000/LY | ACCG dominates | €3,000/LY |
| | - €10m | - €13m | - €19m | €7m |
| | -193 LY | -699 LY | 54 LY | 2,559 LY |
| 55–59 | €30 000/LY | ACCG dominates | ACCG dominates | €1,000/LY |
| | - €13m | - €5m | - €24m | €18m |
| | -435 LY | 574 LY | 1 387 LY | 12,756 LY |
| 50–54 | €41 000/LY | ACCG dominates | €55,000/LY | €2,000/LY |
| | - €35m | - €9m | - €29m | €18m |
| | -843 LY | 1,404 LY | -527 LY | 7,388 LY |
| 45–49 | €69 000/LY | ACCG dominates | €15,000/LY | €3,000/LY |
| | - €48m | - €10m | - €28m | €10m |
| | -698 LY | 1,892 LY | -1 804 LY | 3 191 LY |
| 40–44 | €52 000/LY | ACCG dominates | €45 000/LY | €4,000/LY |
| | - €59m | - €7m | - €18m | €7m |
| | -1,137 LY | 1,734 LY | -397 LY | 1 812 LY |

| | | |
|---|---|---|
| aggregated incremental cost-effectiveness ratio | ACCG dominates | |
| aggregated incremental costs | - €505m | -300 euros per person* |
| aggregated incremental effects | 49,000 LY | 12 days per person* |

*averaged figure in the target population (1.5 million Finns) over the period of study (10-40 years)

**morbidity costs: Initial cost of stroke 7,500€, yearly cost 1,500€. Initial cost of CHD 2,500€, yearly cost 500€.**

blood pressure group classification

**males**

| age group | | BPG 0 | BPG 1 | BPG 2 | BPG 3 |
|---|---|---|---|---|---|
| | 70–74 | €216 000/LY | €73 000/LY | ACCG dominates | ACCG dominated |
| | | - €5m | - €1m | - €5m | €2m |
| | | -23 LY | -15 LY | 19 LY | -65 LY |
| | 65–69 | €103 000/LY | €32 000/LY | ACCG dominates | €7 000/LY |
| | | - €6m | - €4m | - €6m | €3m |
| | | -56 LY | -110 LY | 312 LY | 395 LY |
| | 60–64 | €42 000/LY | €9 000/LY | ACCG dominates | €3 000/LY |
| | | - €9m | - €8m | - €16m | €2m |
| | | -223 LY | -860 LY | 223 LY | 678 LY |
| | 55–59 | €15,000/LY | ACCG dominates | ACCG dominates | €3,000/LY |
| | | - €11m | - €3m | - €12m | €10m |
| | | -746 LY | 146 LY | 1 847 LY | 3 420 LY |
| | 50–54 | €42,000/LY | ACCG dominates | ACCG dominates | €6,000/LY |
| | | - €24m | - €7m | - €25m | €25m |
| | | -572 LY | 1 064 LY | 3,366 LY | 4,476 LY |
| | 45–49 | €35,000/LY | ACCG dominates | ACCG dominates | €10,000/LY |
| | | - €29m | - €10m | - €24m | €21m |
| | | -841 LY | 1 705 LY | 1 171 LY | 2,040 LY |
| | 40–44 | €42,000/LY | ACCG dominates | ACCG dominates | €6,000/LY |
| | | - €38m | - €7m | - €18m | €12m |
| | | -904 LY | 891 LY | 287 LY | 1,898 LY |

**females**

| age group | | BPG 0 | BPG 1 | BPG 2 | BPG 3 |
|---|---|---|---|---|---|
| | 70–74 | ACCG dominates | ACCG dominates | ACCG dominates | €12,000/LY |
| | | - €3m | - €5m | - €11m | €7m |
| | | 4 LY | 15 LY | 332 LY | 567 LY |
| | 65–69 | €94,000/LY | €48,000/LY | €32,000/LY | €4,000/LY |
| | | - €3m | - €12m | - €12m | €6m |
| | | -36 LY | -247 LY | -359 LY | 1,327 LY |
| | 60–64 | €50,000/LY | €19,000/LY | ACCG dominates | €3,000/LY |
| | | - €10m | - €14m | - €19m | €7m |
| | | -193 LY | -699 LY | 54 LY | 2,559 LY |
| | 55–59 | €30 000/LY | ACCG dominates | ACCG dominates | €1,000/LY |
| | | - €13m | - €5m | - €24m | €18m |
| | | -435 LY | 574 LY | 1 387 LY | 12,756 LY |
| | 50–54 | €42 000/LY | ACCG dominates | €56,000/LY | €2,000/LY |
| | | - €35m | - €9m | - €29m | €18m |
| | | -843 LY | 1,404 LY | -527 LY | 7,388 LY |
| | 45–49 | €69 000/LY | ACCG dominates | €15,000/LY | €3,000/LY |
| | | - €48m | - €10m | - €28m | €10m |
| | | -698 LY | 1,892 LY | -1 804 LY | 3 191 LY |
| | 40–44 | €52 000/LY | ACCG dominates | €45 000/LY | €4,000/LY |
| | | - €60m | - €7m | - €18m | €7m |
| | | -1,137 LY | 1,734 LY | -397 LY | 1 812 LY |

| | | | |
|---|---|---|---|
| aggregated incremental cost-effectiveness ratio | ACCG dominates | | |
| aggregated incremental costs | - €491m | -300 | euros per person* |
| aggregated incremental effects | 49,000 LY | 12 | days per person* |

*averaged figure in the target population (1.5 million Finns) over the period of study (10-40 years)

**morbidity costs: Initial cost of stroke 45,000€, yearly cost 10,000€. Initial cost of CHD 15,000€, yearly cost 3,000€.**

blood pressure group classification

**males**

| age group | BPG 0 | BPG 1 | BPG 2 | BPG 3 |
|---|---|---|---|---|
| 70–74 | €204 000/LY | €60 000/LY | ACCG dominates | €1 000/LY |
| | - €5m | - €1m | - €6m | €m |
| | -23 LY | -15 LY | 19 LY | -65 LY |
| 65–69 | €99 000/LY | €28 000/LY | ACCG dominates | ACCG dominates |
| | - €6m | - €3m | - €9m | - €3m |
| | -56 LY | -110 LY | 312 LY | 395 LY |
| 60–64 | €39 000/LY | €7 000/LY | ACCG dominates | ACCG dominates |
| | - €9m | - €6m | - €18m | - €3m |
| | -223 LY | -860 LY | 223 LY | 678 LY |
| 55–59 | €13,000/LY | ACCG dominates | ACCG dominates | ACCG dominates |
| | - €10m | - €4m | - €22m | - €9m |
| | -746 LY | 146 LY | 1 847 LY | 3 420 LY |
| 50–54 | €39,000/LY | ACCG dominates | ACCG dominates | ACCG dominates |
| | - €22m | - €12m | - €45m | - €2m |
| | -572 LY | 1 064 LY | 3,366 LY | 4,476 LY |
| 45–49 | €32,000/LY | ACCG dominates | ACCG dominates | €3,000/LY |
| | - €27m | - €19m | - €34m | €7m |
| | -841 LY | 1 705 LY | 1 171 LY | 2,040 LY |
| 40–44 | €39,000/LY | ACCG dominates | ACCG dominates | ACCG dominates |
| | - €35m | - €13m | - €22m | - €3m |
| | -904 LY | 891 LY | 287 LY | 1,898 LY |

**females**

| age group | BPG 0 | BPG 1 | BPG 2 | BPG 3 |
|---|---|---|---|---|
| 70–74 | ACCG dominates | ACCG dominates | ACCG dominates | €10,000/LY |
| | - €3m | - €4m | - €11m | €6m |
| | 4 LY | 15 LY | 332 LY | 567 LY |
| 65–69 | €82,000/LY | €35,000/LY | €28,000/LY | €4,000/LY |
| | - €3m | - €9m | - €10m | €5m |
| | -36 LY | -247 LY | -359 LY | 1,327 LY |
| 60–64 | €44,000/LY | €15,000/LY | ACCG dominates | €3,000/LY |
| | - €9m | - €10m | - €17m | €7m |
| | -193 LY | -699 LY | 54 LY | 2,559 LY |
| 55–59 | €26 000/LY | ACCG dominates | ACCG dominates | €2,000/LY |
| | - €11m | - €5m | - €20m | €20m |
| | -435 LY | 574 LY | 1 387 LY | 12,756 LY |
| 50–54 | €39 000/LY | ACCG dominates | €52,000/LY | €2,000/LY |
| | - €33m | - €7m | - €27m | €18m |
| | -843 LY | 1,404 LY | -527 LY | 7,388 LY |
| 45–49 | €67 000/LY | ACCG dominates | €15,000/LY | €3,000/LY |
| | - €47m | - €10m | - €27m | €9m |
| | -698 LY | 1,892 LY | -1 804 LY | 3 191 LY |
| 40–44 | €51 000/LY | ACCG dominates | €44 000/LY | €3,000/LY |
| | - €57m | - €7m | - €17m | €6m |
| | -1,137 LY | 1,734 LY | -397 LY | 1 812 LY |

| | | |
|---|---|---|
| aggregated incremental cost-effectiveness ratio | ACCG dominates | |
| aggregated incremental costs | - €614m | -400 euros per person* |
| aggregated incremental effects | 49,000 LY | 12 days per person* |

*averaged figure in the target population (1.5 million Finns) over the period of study (10-40 years)

**morbidity costs: Initial cost of stroke 5,000€, yearly cost 3,000€. Initial cost of CHD 1,500€, yearly cost 1,000€.**

blood pressure group classification

**males**

| age group | BPG 0 | BPG 1 | BPG 2 | BPG 3 |
|---|---|---|---|---|
| 70–74 | €215 000/LY | €71 000/LY | ACCG dominates | ACCG dominated |
| | - €5m | - €1m | - €5m | €2m |
| | -23 LY | -15 LY | 19 LY | -65 LY |
| 65–69 | €102 000/LY | €32 000/LY | ACCG dominates | €5 000/LY |
| | - €6m | - €3m | - €7m | €2m |
| | -56 LY | -110 LY | 312 LY | 395 LY |
| 60–64 | €42 000/LY | €9 000/LY | ACCG dominates | €2 000/LY |
| | - €9m | - €8m | - €16m | €1m |
| | -223 LY | -860 LY | 223 LY | 678 LY |
| 55–59 | €15,000/LY | ACCG dominates | ACCG dominates | €2,000/LY |
| | - €11m | - €3m | - €13m | €7m |
| | -746 LY | 146 LY | 1 847 LY | 3 420 LY |
| 50–54 | €41,000/LY | ACCG dominates | ACCG dominates | €5,000/LY |
| | - €24m | - €8m | - €27m | €21m |
| | -572 LY | 1 064 LY | 3,366 LY | 4,476 LY |
| 45–49 | €34,000/LY | ACCG dominates | ACCG dominates | €9,000/LY |
| | - €29m | - €11m | - €26m | €19m |
| | -841 LY | 1 705 LY | 1 171 LY | 2,040 LY |
| 40–44 | €42,000/LY | ACCG dominates | ACCG dominates | €5,000/LY |
| | - €38m | - €8m | - €18m | €10m |
| | -904 LY | 891 LY | 287 LY | 1,898 LY |

**females**

| age group | BPG 0 | BPG 1 | BPG 2 | BPG 3 |
|---|---|---|---|---|
| 70–74 | ACCG dominates | ACCG dominates | ACCG dominates | €12,000/LY |
| | - €3m | - €4m | - €11m | €7m |
| | 4 LY | 15 LY | 332 LY | 567 LY |
| 65–69 | €93,000/LY | €46,000/LY | €32,000/LY | €4,000/LY |
| | - €3m | - €11m | - €11m | €6m |
| | -36 LY | -247 LY | -359 LY | 1,327 LY |
| 60–64 | €49,000/LY | €19,000/LY | ACCG dominates | €3,000/LY |
| | - €9m | - €13m | - €19m | €7m |
| | -193 LY | -699 LY | 54 LY | 2,559 LY |
| 55–59 | €30 000/LY | ACCG dominates | ACCG dominates | €1,000/LY |
| | - €13m | - €5m | - €23m | €19m |
| | -435 LY | 574 LY | 1 387 LY | 12,756 LY |
| 50–54 | €41 000/LY | ACCG dominates | €55,000/LY | €2,000/LY |
| | - €35m | - €9m | - €29m | €18m |
| | -843 LY | 1,404 LY | -527 LY | 7,388 LY |
| 45–49 | €69 000/LY | ACCG dominates | €15,000/LY | €3,000/LY |
| | - €48m | - €10m | - €28m | €10m |
| | -698 LY | 1,892 LY | -1 804 LY | 3 191 LY |
| 40–44 | €52 000/LY | ACCG dominates | €45 000/LY | €4,000/LY |
| | - €59m | - €7m | - €18m | €7m |
| | -1,137 LY | 1,734 LY | -397 LY | 1 812 LY |

| | | | |
|---|---|---|---|
| aggregated incremental cost-effectiveness ratio | ACCG dominates | | |
| aggregated incremental costs | - €509m | -300 | euros per person* |
| aggregated incremental effects | 49,000 LY | 12 | days per person* |

*averaged figure in the target population (1.5 million Finns) over the period of study (10-40 years)

**no costs associated with morbidity**

blood pressure group classification

**males**

| age group | | BPG 0 | BPG 1 | BPG 2 | BPG 3 |
|---|---|---|---|---|---|
| | 70–74 | €221 000/LY | €78 000/LY | ACCG dominates | ACCG dominated |
| | | - €5m | - €1m | - €4m | €3m |
| | | -23 LY | -15 LY | 19 LY | -65 LY |
| | 65–69 | €104 000/LY | €33 000/LY | ACCG dominates | €12 000/LY |
| | | - €6m | - €4m | - €5m | €5m |
| | | -56 LY | -110 LY | 312 LY | 395 LY |
| | 60–64 | €43 000/LY | €10 000/LY | ACCG dominates | €5 000/LY |
| | | - €10m | - €9m | - €15m | €4m |
| | | -223 LY | -860 LY | 223 LY | 678 LY |
| | 55–59 | €16,000/LY | ACCG dominates | ACCG dominates | €5,000/LY |
| | | - €12m | - €2m | - €8m | €16m |
| | | -746 LY | 146 LY | 1 847 LY | 3 420 LY |
| | 50–54 | €42,000/LY | ACCG dominates | ACCG dominates | €8,000/LY |
| | | - €24m | - €5m | - €18m | €34m |
| | | -572 LY | 1 064 LY | 3,366 LY | 4,476 LY |
| | 45–49 | €35,000/LY | ACCG dominates | ACCG dominates | €12,000/LY |
| | | - €30m | - €6m | - €21m | €25m |
| | | -841 LY | 1 705 LY | 1 171 LY | 2,040 LY |
| | 40–44 | €43,000/LY | ACCG dominates | ACCG dominates | €9,000/LY |
| | | - €39m | - €5m | - €16m | €16m |
| | | -904 LY | 891 LY | 287 LY | 1,898 LY |

**females**

| age group | | BPG 0 | BPG 1 | BPG 2 | BPG 3 |
|---|---|---|---|---|---|
| | 70–74 | ACCG dominates | ACCG dominates | ACCG dominates | €13,000/LY |
| | | - €3m | - €5m | - €11m | €8m |
| | | 4 LY | 15 LY | 332 LY | 567 LY |
| | 65–69 | €99,000/LY | €53,000/LY | €34,000/LY | €4,000/LY |
| | | - €4m | - €13m | - €12m | €6m |
| | | -36 LY | -247 LY | -359 LY | 1,327 LY |
| | 60–64 | €52,000/LY | €21,000/LY | ACCG dominates | €3,000/LY |
| | | - €10m | - €15m | - €20m | €7m |
| | | -193 LY | -699 LY | 54 LY | 2,559 LY |
| | 55–59 | €31 000/LY | ACCG dominates | ACCG dominates | €1,000/LY |
| | | - €14m | - €5m | - €25m | €18m |
| | | -435 LY | 574 LY | 1 387 LY | 12,756 LY |
| | 50–54 | €43 000/LY | ACCG dominates | €57,000/LY | €3,000/LY |
| | | - €36m | - €10m | - €30m | €18m |
| | | -843 LY | 1,404 LY | -527 LY | 7,388 LY |
| | 45–49 | €70 000/LY | ACCG dominates | €16,000/LY | €3,000/LY |
| | | - €49m | - €10m | - €28m | €11m |
| | | -698 LY | 1,892 LY | -1 804 LY | 3 191 LY |
| | 40–44 | €53 000/LY | ACCG dominates | €45 000/LY | €4,000/LY |
| | | - €60m | - €7m | - €18m | €8m |
| | | -1,137 LY | 1,734 LY | -397 LY | 1 812 LY |

| | | | |
|---|---|---|---|
| aggregated incremental cost-effectiveness ratio | ACCG dominates | | |
| aggregated incremental costs | - €451m | -300 | euros per person* |
| aggregated incremental effects | 49,000 LY | 12 | days per person* |

***averaged figure in the target population (1.5 million Finns) over the period of study (10-40 years)

**(under CCG) switching between alternative drug therapies occurs at a rate of 25%**

blood pressure group classification

**males**

| age group | BPG 0 | BPG 1 | BPG 2 | BPG 3 |
|---|---|---|---|---|
| 70–74 | €217 000/LY | €74 000/LY | €451,000/LY | ACCG dominated |
| | - €5m | - €1m | - €6m | €m |
| | -23 LY | -15 LY | -12 LY | -46 LY |
| 65–69 | €103 000/LY | €32 000/LY | ACCG dominates | ACCG dominates |
| | - €6m | - €4m | - €7m | - €1m |
| | -56 LY | -110 LY | 160 LY | 265 LY |
| 60–64 | €42 000/LY | €9 000/LY | €444 000/LY | ACCG dominates |
| | - €9m | - €8m | - €17m | - €1m |
| | -223 LY | -860 LY | -37 LY | 412 LY |
| 55–59 | €15,000/LY | ACCG dominates | ACCG dominates | €2,000/LY |
| | - €11m | - €3m | - €12m | €4m |
| | -746 LY | 146 LY | 1 367 LY | 2 605 LY |
| 50–54 | €42,000/LY | ACCG dominates | ACCG dominates | €4,000/LY |
| | - €24m | - €7m | - €26m | €14m |
| | -572 LY | 1 064 LY | 2,904 LY | 3,238 LY |
| 45–49 | €34,000/LY | ACCG dominates | ACCG dominates | €9,000/LY |
| | - €29m | - €10m | - €25m | €12m |
| | -841 LY | 1 705 LY | 983 LY | 1,309 LY |
| 40–44 | €42,000/LY | ACCG dominates | ACCG dominates | €3,000/LY |
| | - €38m | - €7m | - €18m | €5m |
| | -904 LY | 891 LY | 211 LY | 1,613 LY |

**females**

| age group | BPG 0 | BPG 1 | BPG 2 | BPG 3 |
|---|---|---|---|---|
| 70–74 | ACCG dominates | ACCG dominates | ACCG dominates | €7,000/LY |
| | - €3m | - €5m | - €11m | €2m |
| | 4 LY | 15 LY | 333 LY | 217 LY |
| 65–69 | €95,000/LY | €48,000/LY | €32,000/LY | €1,000/LY |
| | - €3m | - €12m | - €12m | €1m |
| | -36 LY | -247 LY | -367 LY | ,739 LY |
| 60–64 | €50,000/LY | €20,000/LY | ACCG dominates | €1,000/LY |
| | - €10m | - €14m | - €19m | €1m |
| | -193 LY | -699 LY | 54 LY | 1,081 LY |
| 55–59 | €30 000/LY | ACCG dominates | ACCG dominates | €1,000/LY |
| | - €13m | - €5m | - €24m | €8m |
| | -435 LY | 574 LY | 1 370 LY | 8,884 LY |
| 50–54 | €42 000/LY | ACCG dominates | €51,000/LY | €2,000/LY |
| | - €35m | - €9m | - €29m | €8m |
| | -843 LY | 1,404 LY | -576 LY | 4,843 LY |
| 45–49 | €69 000/LY | ACCG dominates | €15,000/LY | €2,000/LY |
| | - €48m | - €10m | - €28m | €5m |
| | -698 LY | 1,892 LY | -1 804 LY | 2 193 LY |
| 40–44 | €52 000/LY | ACCG dominates | €45 000/LY | €3,000/LY |
| | - €60m | - €7m | - €18m | €4m |
| | -1,137 LY | 1,734 LY | -397 LY | 1 302 LY |

| | | |
|---|---|---|
| aggregated incremental cost-effectiveness ratio | ACCG dominates | |
| aggregated incremental costs | - €585m | -400 euros per person* |
| aggregated incremental effects | 34,000 LY | 8 days per person* |

*averaged figure in the target population (1.5 million Finns) over the period of study (10-40 years)

**(under CCG) switching between alternative drug therapies occurs at a rate of 65%**

blood pressure group classification

**males**

| age group | | BPG 0 | BPG 1 | BPG 2 | BPG 3 |
|---|---|---|---|---|---|
| 70–74 | | €217 000/LY | €74 000/LY | ACCG dominates | ACCG dominated |
| | | - €5m | - €1m | - €4m | €6m |
| | | -23 LY | -15 LY | 67 LY | -90 LY |
| 65–69 | | €103 000/LY | €32 000/LY | ACCG dominates | €14 000/LY |
| | | - €6m | - €4m | - €5m | €8m |
| | | -56 LY | -110 LY | 546 LY | 572 LY |
| 60–64 | | €42 000/LY | €9 000/LY | ACCG dominates | €5 000/LY |
| | | - €9m | - €8m | - €15m | €6m |
| | | -223 LY | -860 LY | 603 LY | 1 060 LY |
| 55–59 | | €15,000/LY | ACCG dominates | ACCG dominates | €4,000/LY |
| | | - €11m | - €3m | - €12m | €16m |
| | | -746 LY | 146 LY | 2 567 LY | 4 517 LY |
| 50–54 | | €42,000/LY | ACCG dominates | ACCG dominates | €6,000/LY |
| | | - €24m | - €7m | - €25m | €38m |
| | | -572 LY | 1 064 LY | 4,047 LY | 6,261 LY |
| 45–49 | | €34,000/LY | ACCG dominates | ACCG dominates | €10,000/LY |
| | | - €29m | - €10m | - €25m | €31m |
| | | -841 LY | 1 705 LY | 1 439 LY | 3,061 LY |
| 40–44 | | €42,000/LY | ACCG dominates | ACCG dominates | €7,000/LY |
| | | - €38m | - €7m | - €18m | €18m |
| | | -904 LY | 891 LY | 397 LY | 2,364 LY |

**females**

| age group | | BPG 0 | BPG 1 | BPG 2 | BPG 3 |
|---|---|---|---|---|---|
| 70–74 | | ACCG dominates | ACCG dominates | ACCG dominates | €14,000/LY |
| | | - €3m | - €5m | - €11m | €15m |
| | | 4 LY | 15 LY | 332 LY | 1 052 LY |
| 65–69 | | €95,000/LY | €48,000/LY | €33,000/LY | €6,000/LY |
| | | - €3m | - €12m | - €11m | €13m |
| | | -36 LY | -247 LY | -347 LY | 2,145 LY |
| 60–64 | | €50,000/LY | €20,000/LY | ACCG dominates | €3,000/LY |
| | | - €10m | - €14m | - €19m | €16m |
| | | -193 LY | -699 LY | 54 LY | 4,713 LY |
| 55–59 | | €30 000/LY | ACCG dominates | ACCG dominates | €2,000/LY |
| | | - €13m | - €5m | - €24m | €33m |
| | | -435 LY | 574 LY | 1 412 LY | 17,898 LY |
| 50–54 | | €42 000/LY | ACCG dominates | €64,000/LY | €3,000/LY |
| | | - €35m | - €9m | - €29m | €33m |
| | | -843 LY | 1,404 LY | -456 LY | 10,857 LY |
| 45–49 | | €69 000/LY | ACCG dominates | €15,000/LY | €4,000/LY |
| | | - €48m | - €10m | - €28m | €18m |
| | | -698 LY | 1,892 LY | -1 804 LY | 4 636 LY |
| 40–44 | | €52 000/LY | ACCG dominates | €45 000/LY | €5,000/LY |
| | | - €60m | - €7m | - €18m | €12m |
| | | -1,137 LY | 1,734 LY | -397 LY | 2 529 LY |

| | | |
|---|---|---|
| aggregated incremental cost-effectiveness ratio | ACCG dominates | |
| aggregated incremental costs | - €375m | -300 euros per person* |
| aggregated incremental effects | 71,000 LY | 17 days per person* |

*averaged figure in the target population (1.5 million Finns) over the period of study (10-40 years)

**(under CCG) switching between alternative drug therapies occurs at a rate of 85%**

blood pressure group classification

**males** (age group)

| | BPG 0 | BPG 1 | BPG 2 | BPG 3 |
|---|---|---|---|---|
| 70–74 | €217 000/LY | €74 000/LY | ACCG dominates | ACCG dominated |
| | - €5m | - €1m | - €2m | €10m |
| | -23 LY | -15 LY | 133 LY | -122 LY |
| 65–69 | €103 000/LY | €32 000/LY | ACCG dominates | €19 000/LY |
| | - €6m | - €4m | - €3m | €15m |
| | -56 LY | -110 LY | 877 LY | 804 LY |
| 60–64 | €42 000/LY | €9 000/LY | ACCG dominates | €7 000/LY |
| | - €9m | - €8m | - €14m | €11m |
| | -223 LY | -860 LY | 1 121 LY | 1 577 LY |
| 55–59 | €15,000/LY | ACCG dominates | ACCG dominates | €4,000/LY |
| | - €11m | - €3m | - €12m | €25m |
| | -746 LY | 146 LY | 3 568 LY | 5 934 LY |
| 50–54 | €42,000/LY | ACCG dominates | ACCG dominates | €7,000/LY |
| | - €24m | - €7m | - €25m | €57m |
| | -572 LY | 1 064 LY | 4,985 LY | 8,682 LY |
| 45–49 | €34,000/LY | ACCG dominates | ACCG dominates | €10,000/LY |
| | - €29m | - €10m | - €24m | €46m |
| | -841 LY | 1 705 LY | 1 800 LY | 4,413 LY |
| 40–44 | €42,000/LY | ACCG dominates | ACCG dominates | €9,000/LY |
| | - €38m | - €7m | - €18m | €27m |
| | -904 LY | 891 LY | 547 LY | 3,047 LY |

**females** (age group)

| | BPG 0 | BPG 1 | BPG 2 | BPG 3 |
|---|---|---|---|---|
| 70–74 | ACCG dominates | ACCG dominates | ACCG dominates | €16,000/LY |
| | - €3m | - €5m | - €11m | €27m |
| | 4 LY | 15 LY | 331 LY | 1 692 LY |
| 65–69 | €95,000/LY | €48,000/LY | €34,000/LY | €7,000/LY |
| | - €3m | - €12m | - €11m | €23m |
| | -36 LY | -247 LY | -331 LY | 3,228 LY |
| 60–64 | €50,000/LY | €20,000/LY | ACCG dominates | €4,000/LY |
| | - €10m | - €14m | - €19m | €29m |
| | -193 LY | -699 LY | 54 LY | 7,654 LY |
| 55–59 | €30 000/LY | ACCG dominates | ACCG dominates | €2,000/LY |
| | - €13m | - €5m | - €24m | €54m |
| | -435 LY | 574 LY | 1 445 LY | 24,468 LY |
| 50–54 | €42 000/LY | ACCG dominates | €80,000/LY | €4,000/LY |
| | - €35m | - €9m | - €29m | €54m |
| | -843 LY | 1,404 LY | -361 LY | 15,378 LY |
| 45–49 | €69 000/LY | ACCG dominates | €15,000/LY | €4,000/LY |
| | - €48m | - €10m | - €28m | €28m |
| | -698 LY | 1,892 LY | -1 804 LY | 6 604 LY |
| 40–44 | €52 000/LY | ACCG dominates | €45 000/LY | €5,000/LY |
| | - €60m | - €7m | - €18m | €19m |
| | -1,137 LY | 1,734 LY | -397 LY | 3 484 LY |

| | | |
|---|---|---|
| aggregated incremental cost-effectiveness ratio | ACCG dominates | |
| aggregated incremental costs | - €206m | -100 euros per person* |
| aggregated incremental effects | 100,000 LY | 24 days per person* |

*averaged figure in the target population (1.5 million Finns) over the period of study (10-40 years)

**20% of initial pharmacological therapies are thiazide diuretics - ACE inhibitors, angiotensin II antagonists, beta blocking agents and calcium channel blockers are the equally likely alternatives**

blood pressure group classification

**males**

| age group | BPG 0 | BPG 1 | BPG 2 | BPG 3 |
|---|---|---|---|---|
| 70–74 | €217 000/LY | €74 000/LY | ACCG dominates | ACCG dominated |
| | - €5m | - €1m | - €3m | €5m |
| | -23 LY | -15 LY | 19 LY | -65 LY |
| 65–69 | €103 000/LY | €32 000/LY | ACCG dominates | €17 000/LY |
| | - €6m | - €4m | - €3m | €7m |
| | -56 LY | -110 LY | 310 LY | 394 LY |
| 60–64 | €42 000/LY | €9 000/LY | ACCG dominates | €7 000/LY |
| | - €9m | - €8m | - €13m | €5m |
| | -223 LY | -860 LY | 221 LY | 677 LY |
| 55–59 | €15,000/LY | ACCG dominates | ACCG dominates | €4,000/LY |
| | - €11m | - €3m | - €10m | €15m |
| | -746 LY | 146 LY | 1 843 LY | 3 417 LY |
| 50–54 | €42,000/LY | ACCG dominates | ACCG dominates | €8,000/LY |
| | - €24m | - €7m | - €22m | €36m |
| | -572 LY | 1 064 LY | 3,362 LY | 4,466 LY |
| 45–49 | €34,000/LY | ACCG dominates | ACCG dominates | €14,000/LY |
| | - €29m | - €10m | - €23m | €29m |
| | -841 LY | 1 705 LY | 1 169 LY | 2,036 LY |
| 40–44 | €42,000/LY | ACCG dominates | ACCG dominates | €9,000/LY |
| | - €38m | - €7m | - €17m | €17m |
| | -904 LY | 891 LY | 286 LY | 1,894 LY |

**females**

| age group | BPG 0 | BPG 1 | BPG 2 | BPG 3 |
|---|---|---|---|---|
| 70–74 | ACCG dominates | ACCG dominates | ACCG dominates | €22,000/LY |
| | - €3m | - €5m | - €11m | €12m |
| | 4 LY | 15 LY | 332 LY | 565 LY |
| 65–69 | €95,000/LY | €48,000/LY | €32,000/LY | €8,000/LY |
| | - €3m | - €12m | - €11m | €10m |
| | -36 LY | -247 LY | -359 LY | 1,323 LY |
| 60–64 | €50,000/LY | €20,000/LY | ACCG dominates | €5,000/LY |
| | - €10m | - €14m | - €19m | €13m |
| | -193 LY | -699 LY | 54 LY | 2,547 LY |
| 55–59 | €30 000/LY | ACCG dominates | ACCG dominates | €2,000/LY |
| | - €13m | - €5m | - €24m | €27m |
| | -435 LY | 574 LY | 1 387 LY | 12,743 LY |
| 50–54 | €42 000/LY | ACCG dominates | €55,000/LY | €4,000/LY |
| | - €35m | - €9m | - €29m | €28m |
| | -843 LY | 1,404 LY | -527 LY | 7,376 LY |
| 45–49 | €69 000/LY | ACCG dominates | €15,000/LY | €5,000/LY |
| | - €48m | - €10m | - €28m | €16m |
| | -698 LY | 1,892 LY | -1 804 LY | 3 183 LY |
| 40–44 | €52 000/LY | ACCG dominates | €45 000/LY | €6,000/LY |
| | - €60m | - €7m | - €18m | €11m |
| | -1,137 LY | 1,734 LY | -397 LY | 1 809 LY |

| | | |
|---|---|---|
| aggregated incremental cost-effectiveness ratio | ACCG dominates | |
| aggregated incremental costs | - €394m | -300 euros per person* |
| aggregated incremental effects | 49,000 LY | 12 days per person* |

*averaged figure in the target population (1.5 million Finns) over the period of study (10-40 years)

**40% of initial pharmacological therapies are thiazide diuretics - ACE inhibitors, angiotensin II antagonists, beta blocking agents and calcium channel blockers are the equally likely alternatives**

blood pressure group classification

**males** (age group)

| | BPG 0 | BPG 1 | BPG 2 | BPG 3 |
|---|---|---|---|---|
| 70–74 | €217 000/LY | €74 000/LY | ACCG dominates | ACCG dominated |
| | - €5m | - €1m | - €4m | €4m |
| | -23 LY | -15 LY | 19 LY | -65 LY |
| 65–69 | €103 000/LY | €32 000/LY | ACCG dominates | €12 000/LY |
| | - €6m | - €4m | - €5m | €5m |
| | -56 LY | -110 LY | 311 LY | 394 LY |
| 60–64 | €42 000/LY | €9 000/LY | ACCG dominates | €5 000/LY |
| | - €9m | - €8m | - €15m | €3m |
| | -223 LY | -860 LY | 222 LY | 677 LY |
| 55–59 | €15,000/LY | ACCG dominates | ACCG dominates | €4,000/LY |
| | - €11m | - €3m | - €11m | €12m |
| | -746 LY | 146 LY | 1 845 LY | 3 418 LY |
| 50–54 | €42,000/LY | ACCG dominates | ACCG dominates | €7,000/LY |
| | - €24m | - €7m | - €24m | €30m |
| | -572 LY | 1 064 LY | 3,364 LY | 4,471 LY |
| 45–49 | €34,000/LY | ACCG dominates | ACCG dominates | €12,000/LY |
| | - €29m | - €10m | - €24m | €25m |
| | -841 LY | 1 705 LY | 1 170 LY | 2,038 LY |
| 40–44 | €42,000/LY | ACCG dominates | ACCG dominates | €7,000/LY |
| | - €38m | - €7m | - €18m | €14m |
| | -904 LY | 891 LY | 287 LY | 1,896 LY |

**females** (age group)

| | BPG 0 | BPG 1 | BPG 2 | BPG 3 |
|---|---|---|---|---|
| 70–74 | ACCG dominates | ACCG dominates | ACCG dominates | €17,000/LY |
| | - €3m | - €5m | - €11m | €10m |
| | 4 LY | 15 LY | 332 LY | 566 LY |
| 65–69 | €95,000/LY | €48,000/LY | €32,000/LY | €6,000/LY |
| | - €3m | - €12m | - €11m | €8m |
| | -36 LY | -247 LY | -359 LY | 1,325 LY |
| 60–64 | €50,000/LY | €20,000/LY | ACCG dominates | €4,000/LY |
| | - €10m | - €14m | - €19m | €10m |
| | -193 LY | -699 LY | 54 LY | 2,553 LY |
| 55–59 | €30 000/LY | ACCG dominates | ACCG dominates | €2,000/LY |
| | - €13m | - €5m | - €24m | €23m |
| | -435 LY | 574 LY | 1 387 LY | 12,750 LY |
| 50–54 | €42 000/LY | ACCG dominates | €55,000/LY | €3,000/LY |
| | - €35m | - €9m | - €29m | €23m |
| | -843 LY | 1,404 LY | -527 LY | 7,382 LY |
| 45–49 | €69 000/LY | ACCG dominates | €15,000/LY | €4,000/LY |
| | - €48m | - €10m | - €28m | €13m |
| | -698 LY | 1,892 LY | -1 804 LY | 3 187 LY |
| 40–44 | €52 000/LY | ACCG dominates | €45 000/LY | €5,000/LY |
| | - €60m | - €7m | - €18m | €9m |
| | -1,137 LY | 1,734 LY | -397 LY | 1 811 LY |

| | | |
|---|---|---|
| aggregated incremental cost-effectiveness ratio | ACCG dominates | |
| aggregated incremental costs | - €446m | -300 euros per person* |
| aggregated incremental effects | 49,000 LY | 12 days per person* |

*averaged figure in the target population (1.5 million Finns) over the period of study (10-40 years)

**80% of initial pharmacological therapies are thiazide diuretics - ACE inhibitors, angiotensin II antagonists, beta blocking agents and calcium channel blockers are the equally likely alternatives**

blood pressure group classification

**males**

| age group | BPG 0 | BPG 1 | BPG 2 | BPG 3 |
|---|---|---|---|---|
| 70–74 | €217 000/LY | €74 000/LY | ACCG dominates | ACCG dominated |
| | - €5m | - €1m | - €6m | €1m |
| | -23 LY | -15 LY | 19 LY | -65 LY |
| 65–69 | €103 000/LY | €32 000/LY | ACCG dominates | €2 000/LY |
| | - €6m | - €4m | - €8m | €1m |
| | -56 LY | -110 LY | 313 LY | 395 LY |
| 60–64 | €42 000/LY | €9 000/LY | ACCG dominates | €/LY |
| | - €9m | - €8m | - €17m | €m |
| | -223 LY | -860 LY | 224 LY | 679 LY |
| 55–59 | €15,000/LY | ACCG dominates | ACCG dominates | €2,000/LY |
| | - €11m | - €3m | - €13m | €6m |
| | -746 LY | 146 LY | 1 850 LY | 3 421 LY |
| 50–54 | €42,000/LY | ACCG dominates | ACCG dominates | €4,000/LY |
| | - €24m | - €7m | - €27m | €17m |
| | -572 LY | 1 064 LY | 3,368 LY | 4,480 LY |
| 45–49 | €34,000/LY | ACCG dominates | ACCG dominates | €7,000/LY |
| | - €29m | - €10m | - €25m | €15m |
| | -841 LY | 1 705 LY | 1 171 LY | 2,042 LY |
| 40–44 | €42,000/LY | ACCG dominates | ACCG dominates | €4,000/LY |
| | - €38m | - €7m | - €18m | €7m |
| | -904 LY | 891 LY | 287 LY | 1,900 LY |

**females**

| age group | BPG 0 | BPG 1 | BPG 2 | BPG 3 |
|---|---|---|---|---|
| 70–74 | ACCG dominates | ACCG dominates | ACCG dominates | €8,000/LY |
| | - €3m | - €5m | - €11m | €4m |
| | 4 LY | 15 LY | 332 LY | 568 LY |
| 65–69 | €95,000/LY | €48,000/LY | €33,000/LY | €3,000/LY |
| | - €3m | - €12m | - €12m | €3m |
| | -36 LY | -247 LY | -359 LY | 1,328 LY |
| 60–64 | €50,000/LY | €20,000/LY | ACCG dominates | €2,000/LY |
| | - €10m | - €14m | - €19m | €4m |
| | -193 LY | -699 LY | 54 LY | 2,565 LY |
| 55–59 | €30 000/LY | ACCG dominates | ACCG dominates | €1,000/LY |
| | - €13m | - €5m | - €24m | €14m |
| | -435 LY | 574 LY | 1 387 LY | 12,762 LY |
| 50–54 | €42 000/LY | ACCG dominates | €56,000/LY | €2,000/LY |
| | - €35m | - €9m | - €29m | €13m |
| | -843 LY | 1,404 LY | -526 LY | 7,393 LY |
| 45–49 | €69 000/LY | ACCG dominates | €15,000/LY | €2,000/LY |
| | - €48m | - €10m | - €28m | €7m |
| | -698 LY | 1,892 LY | -1 804 LY | 3 194 LY |
| 40–44 | €52 000/LY | ACCG dominates | €45 000/LY | €3,000/LY |
| | - €60m | - €7m | - €18m | €5m |
| | -1,137 LY | 1,734 LY | -397 LY | 1 814 LY |

| | | |
|---|---|---|
| aggregated incremental cost-effectiveness ratio | ACCG dominates | |
| aggregated incremental costs | - €550m | -400 euros per person* |
| aggregated incremental effects | 49,000 LY | 12 days per person* |

*averaged figure in the target population (1.5 million Finns) over the period of study (10-40 years)

**100% of initial pharmacological therapies are thiazide diuretics**

blood pressure group classification

**males**

| age group | BPG 0 | BPG 1 | BPG 2 | BPG 3 |
|---|---|---|---|---|
| 70–74 | €217 000/LY | €74 000/LY | ACCG dominates | ACCG dominated |
| | - €5m | - €1m | - €7m | €m |
| | -23 LY | -15 LY | 20 LY | -65 LY |
| 65–69 | €103 000/LY | €32 000/LY | ACCG dominates | ACCG dominates |
| | - €6m | - €4m | - €9m | - €1m |
| | -56 LY | -110 LY | 313 LY | 395 LY |
| 60–64 | €42 000/LY | €9 000/LY | ACCG dominates | ACCG dominates |
| | - €9m | - €8m | - €18m | - €1m |
| | -223 LY | -860 LY | 225 LY | 680 LY |
| 55–59 | €15,000/LY | ACCG dominates | ACCG dominates | €1,000/LY |
| | - €11m | - €3m | - €14m | €3m |
| | -746 LY | 146 LY | 1 852 LY | 3 423 LY |
| 50–54 | €42,000/LY | ACCG dominates | ACCG dominates | €2,000/LY |
| | - €24m | - €7m | - €29m | €11m |
| | -572 LY | 1 064 LY | 3,370 LY | 4,485 LY |
| 45–49 | €34,000/LY | ACCG dominates | ACCG dominates | €5,000/LY |
| | - €29m | - €10m | - €26m | €11m |
| | -841 LY | 1 705 LY | 1 172 LY | 2,044 LY |
| 40–44 | €42,000/LY | ACCG dominates | ACCG dominates | €2,000/LY |
| | - €38m | - €7m | - €19m | €4m |
| | -904 LY | 891 LY | 288 LY | 1,902 LY |

**females**

| age group | BPG 0 | BPG 1 | BPG 2 | BPG 3 |
|---|---|---|---|---|
| 70–74 | ACCG dominates | ACCG dominates | ACCG dominates | €3,000/LY |
| | - €3m | - €5m | - €11m | €2m |
| | 4 LY | 15 LY | 332 LY | 569 LY |
| 65–69 | €95,000/LY | €48,000/LY | €33,000/LY | €1,000/LY |
| | - €3m | - €12m | - €12m | €1m |
| | -36 LY | -247 LY | -359 LY | 1,330 LY |
| 60–64 | €50,000/LY | €20,000/LY | ACCG dominates | €1,000/LY |
| | - €10m | - €14m | - €19m | €1m |
| | -193 LY | -699 LY | 54 LY | 2,571 LY |
| 55–59 | €30 000/LY | ACCG dominates | ACCG dominates | €1,000/LY |
| | - €13m | - €5m | - €24m | €10m |
| | -435 LY | 574 LY | 1 387 LY | 12,768 LY |
| 50–54 | €42 000/LY | ACCG dominates | €56,000/LY | €1,000/LY |
| | - €35m | - €9m | - €30m | €9m |
| | -843 LY | 1,404 LY | -526 LY | 7,399 LY |
| 45–49 | €69 000/LY | ACCG dominates | €15,000/LY | €1,000/LY |
| | - €48m | - €10m | - €28m | €5m |
| | -698 LY | 1,892 LY | -1 804 LY | 3 198 LY |
| 40–44 | €52 000/LY | ACCG dominates | €45 000/LY | €2,000/LY |
| | - €60m | - €7m | - €18m | €3m |
| | -1,137 LY | 1,734 LY | -397 LY | 1 815 LY |

| | | |
|---|---|---|
| aggregated incremental cost-effectiveness ratio | ACCG dominates | |
| aggregated incremental costs | - €602m | -400 euros per person* |
| aggregated incremental effects | 49,000 LY | 12 days per person* |

*averaged figure in the target population (1.5 million Finns) over the period of study (10-40 years)

**benefit discount rate = 3%, cost discount rate = 6%**

blood pressure group classification

**males**

| age group | | BPG 0 | BPG 1 | BPG 2 | BPG 3 |
|---|---|---|---|---|---|
| | 70–74 | €184 000/LY | €62 000/LY | ACCG dominates | ACCG dominated |
| | | - €5m | - €1m | - €4m | €2m |
| | | -26 LY | -17 LY | 18 LY | -90 LY |
| | 65–69 | €79 000/LY | €24 000/LY | ACCG dominates | €6 000/LY |
| | | - €5m | - €3m | - €6m | €3m |
| | | -69 LY | -136 LY | 378 LY | 470 LY |
| | 60–64 | €30 000/LY | €7 000/LY | ACCG dominates | €2 000/LY |
| | | - €9m | - €7m | - €15m | €2m |
| | | -293 LY | -1 131 LY | 287 LY | 876 LY |
| | 55–59 | €10,000/LY | ACCG dominates | ACCG dominates | €2,000/LY |
| | | - €11m | - €3m | - €11m | €9m |
| | | -1 028 LY | 202 LY | 2 496 LY | 4 625 LY |
| | 50–54 | €26,000/LY | ACCG dominates | ACCG dominates | €3,000/LY |
| | | - €22m | - €6m | - €23m | €23m |
| | | -852 LY | 1 595 LY | 4,912 LY | 6,549 LY |
| | 45–49 | €20,000/LY | ACCG dominates | ACCG dominates | €6,000/LY |
| | | - €27m | - €9m | - €22m | €19m |
| | | -1 341 LY | 2 752 LY | 1 770 LY | 3,157 LY |
| | 40–44 | €23,000/LY | ACCG dominates | ACCG dominates | €3,000/LY |
| | | - €35m | - €6m | - €16m | €10m |
| | | -1 525 LY | 1 526 LY | 407 LY | 3,050 LY |

**females**

| age group | | BPG 0 | BPG 1 | BPG 2 | BPG 3 |
|---|---|---|---|---|---|
| | 70–74 | ACCG dominates | ACCG dominates | ACCG dominates | €10,000/LY |
| | | - €3m | - €4m | - €11m | €7m |
| | | 6 LY | 22 LY | 398 LY | 672 LY |
| | 65–69 | €74,000/LY | €38,000/LY | €24,000/LY | €3,000/LY |
| | | - €3m | - €11m | - €11m | €5m |
| | | -43 LY | -299 LY | -453 LY | 1,667 LY |
| | 60–64 | €35,000/LY | €14,000/LY | ACCG dominates | €2,000/LY |
| | | - €9m | - €13m | - €18m | €7m |
| | | -256 LY | -932 LY | 27 LY | 3,374 LY |
| | 55–59 | €20 000/LY | ACCG dominates | ACCG dominates | €1,000/LY |
| | | - €12m | - €5m | - €22m | €17m |
| | | -616 LY | 853 LY | 1 784 LY | 17,533 LY |
| | 50–54 | €25 000/LY | ACCG dominates | €31,000/LY | €2,000/LY |
| | | - €32m | - €8m | - €27m | €17m |
| | | -1306 LY | 2,114 LY | -859 LY | 11,106 LY |
| | 45–49 | €38 000/LY | ACCG dominates | €8,000/LY | €2,000/LY |
| | | - €44m | - €9m | - €25m | €9m |
| | | -1162 LY | 3,099 LY | -2 979 LY | 5 192 LY |
| | 40–44 | €27 000/LY | ACCG dominates | €22 000/LY | €2,000/LY |
| | | - €54m | - €6m | - €16m | €6m |
| | | -2,031 LY | 3,031 LY | -727 LY | 3 146 LY |

| | | |
|---|---|---|
| aggregated incremental cost-effectiveness ratio | ACCG dominates | |
| aggregated incremental costs | - €450m | -300 euros per person* |
| aggregated incremental effects | 71,000 LY | 17 days per person* |

*averaged figure in the target population (1.5 million Finns) over the period of study (10-40 years)

# Sensitivity analysis



**benefit discount rate = 3%, cost discount rate = 3%**

blood pressure group classification

**males**

| age group | BPG 0 | BPG 1 | BPG 2 | BPG 3 |
|---|---|---|---|---|
| 70–74 | €209 000/LY | €71 000/LY | ACCG dominates | ACCG dominated |
| | - €5m | - €1m | - €5m | €2m |
| | -26 LY | -17 LY | 18 LY | -90 LY |
| 65–69 | €93 000/LY | €29 000/LY | ACCG dominates | €5 000/LY |
| | - €6m | - €4m | - €7m | €2m |
| | -69 LY | -136 LY | 378 LY | 470 LY |
| 60–64 | €37 000/LY | €8 000/LY | ACCG dominates | €2 000/LY |
| | - €11m | - €9m | - €18m | €2m |
| | -293 LY | -1 131 LY | 287 LY | 876 LY |
| 55–59 | €13,000/LY | ACCG dominates | ACCG dominates | €2,000/LY |
| | - €13m | - €4m | - €15m | €9m |
| | -1 028 LY | 202 LY | 2 496 LY | 4 625 LY |
| 50–54 | €33,000/LY | ACCG dominates | ACCG dominates | €4,000/LY |
| | - €28m | - €10m | - €33m | €25m |
| | -852 LY | 1 595 LY | 4,912 LY | 6,549 LY |
| 45–49 | €26,000/LY | ACCG dominates | ACCG dominates | €7,000/LY |
| | - €35m | - €14m | - €32m | €22m |
| | -1 341 LY | 2 752 LY | 1 770 LY | 3,157 LY |
| 40–44 | €31,000/LY | ACCG dominates | ACCG dominates | €4,000/LY |
| | - €47m | - €10m | - €23m | €12m |
| | -1 525 LY | 1 526 LY | 407 LY | 3,050 LY |

**females**

| age group | BPG 0 | BPG 1 | BPG 2 | BPG 3 |
|---|---|---|---|---|
| 70–74 | ACCG dominates | ACCG dominates | ACCG dominates | €11,000/LY |
| | - €3m | - €5m | - €12m | €8m |
| | 6 LY | 22 LY | 398 LY | 672 LY |
| 65–69 | €88,000/LY | €45,000/LY | €29,000/LY | €4,000/LY |
| | - €4m | - €13m | - €13m | €6m |
| | -43 LY | -299 LY | -453 LY | 1,667 LY |
| 60–64 | €43,000/LY | €17,000/LY | ACCG dominates | €2,000/LY |
| | - €11m | - €16m | - €22m | €8m |
| | -256 LY | -932 LY | 27 LY | 3,374 LY |
| 55–59 | €25 000/LY | ACCG dominates | ACCG dominates | €1,000/LY |
| | - €15m | - €6m | - €28m | €22m |
| | -616 LY | 853 LY | 1 784 LY | 17,533 LY |
| 50–54 | €32 000/LY | ACCG dominates | €42,000/LY | €2,000/LY |
| | - €42m | - €11m | - €36m | €22m |
| | -1306 LY | 2,114 LY | -859 LY | 11,106 LY |
| 45–49 | €51 000/LY | ACCG dominates | €12,000/LY | €2,000/LY |
| | - €59m | - €13m | - €35m | €13m |
| | -1162 LY | 3,099 LY | -2 979 LY | 5 192 LY |
| 40–44 | €37 000/LY | ACCG dominates | €31 000/LY | €3,000/LY |
| | - €74m | - €9m | - €23m | €9m |
| | -2,031 LY | 3,031 LY | -727 LY | 3 146 LY |

| | | |
|---|---|---|
| aggregated incremental cost-effectiveness ratio | ACCG dominates | |
| aggregated incremental costs | - €621m | -400 euros per person* |
| aggregated incremental effects | 71,000 LY | 17 days per person* |

*averaged figure in the target population (1.5 million Finns) over the period of study (10-40 years)

**benefit discount rate = 0%, cost discount rate = 0%**

blood pressure group classification

**males**

| age group | BPG 0 | BPG 1 | BPG 2 | BPG 3 |
|---|---|---|---|---|
| 70–74 | €201 000/LY | €69 000/LY | ACCG dominates | ACCG dominated |
| | - €6m | - €1m | - €7m | €2m |
| | -31 LY | -20 LY | 13 LY | -144 LY |
| 65–69 | €81 000/LY | €25 000/LY | ACCG dominates | €3 000/LY |
| | - €8m | - €5m | - €10m | €2m |
| | -96 LY | -190 LY | 505 LY | 608 LY |
| 60–64 | €30 000/LY | €7 000/LY | ACCG dominates | €1 000/LY |
| | - €13m | - €11m | - €24m | €1m |
| | -449 LY | -1 736 LY | 424 LY | 1 303 LY |
| 55–59 | €10,000/LY | ACCG dominates | ACCG dominates | €1,000/LY |
| | - €17m | - €6m | - €22m | €8m |
| | -1 709 LY | 334 LY | 4 020 LY | 7 458 LY |
| 50–54 | €24,000/LY | ACCG dominates | ACCG dominates | €2,000/LY |
| | - €38m | - €16m | - €50m | €27m |
| | -1 603 LY | 3 015 LY | 8,940 LY | 11,967 LY |
| 45–49 | €17,000/LY | ACCG dominates | ACCG dominates | €4,000/LY |
| | - €49m | - €25m | - €48m | €27m |
| | -2 823 LY | 5 850 LY | 3 443 LY | 6,355 LY |
| 40–44 | €19,000/LY | ACCG dominates | ACCG dominates | €2,000/LY |
| | - €67m | - €19m | - €36m | €13m |
| | -3 551 LY | 3 595 LY | 710 LY | 6,617 LY |

**females**

| age group | BPG 0 | BPG 1 | BPG 2 | BPG 3 |
|---|---|---|---|---|
| 70–74 | ACCG dominates | ACCG dominates | ACCG dominates | €9,000/LY |
| | - €4m | - €6m | - €14m | €8m |
| | 9 LY | 36 LY | 526 LY | 872 LY |
| 65–69 | €79,000/LY | €41,000/LY | €25,000/LY | €3,000/LY |
| | - €5m | - €16m | - €16m | €7m |
| | -58 LY | -399 LY | -649 LY | 2,377 LY |
| 60–64 | €35,000/LY | €14,000/LY | €470 000/LY | €2,000/LY |
| | - €14m | - €20m | - €28m | €9m |
| | -398 LY | -1 460 LY | -59 LY | 5,212 LY |
| 55–59 | €19 000/LY | ACCG dominates | ACCG dominates | €1,000/LY |
| | - €20m | - €9m | - €38m | €30m |
| | -1 064 LY | 1579 LY | 2 652 LY | 29,105 LY |
| 50–54 | €22 000/LY | ACCG dominates | €28,000/LY | €1,000/LY |
| | - €57m | - €16m | - €51m | €31m |
| | -2604 LY | 4,047 LY | -1 843 LY | 21,190 LY |
| 45–49 | €32 000/LY | ACCG dominates | €8,000/LY | €2,000/LY |
| | - €85m | - €19m | - €52m | €19m |
| | -2604 LY | 6,785 LY | -6 600 LY | 11 253 LY |
| 40–44 | €22 000/LY | ACCG dominates | €19 000/LY | €2,000/LY |
| | - €111m | - €14m | - €36m | €14m |
| | -5,119 LY | 7,411 LY | -1 889 LY | 7 627 LY |

| | | |
|---|---|---|
| aggregated incremental cost-effectiveness ratio | ACCG dominates | |
| aggregated incremental costs | - €908m | -600 euros per person* |
| aggregated incremental effects | 129,000 LY | 31 days per person* |

*averaged figure in the target population (1.5 million Finns) over the period of study (10-40 years)
