# Supplementary material for: Cost-effectiveness analysis of guidelines for antihypertensive care in Finland
Source: BMC Health Serv Res. 2007 Oct 24;7:172. doi: 10.1186/1472-6963-7-172 (PMC2174470; doi:10.1186/1472-6963-7-172)

**Figure 1: Size of study population age groups – population weights used, by gender.**

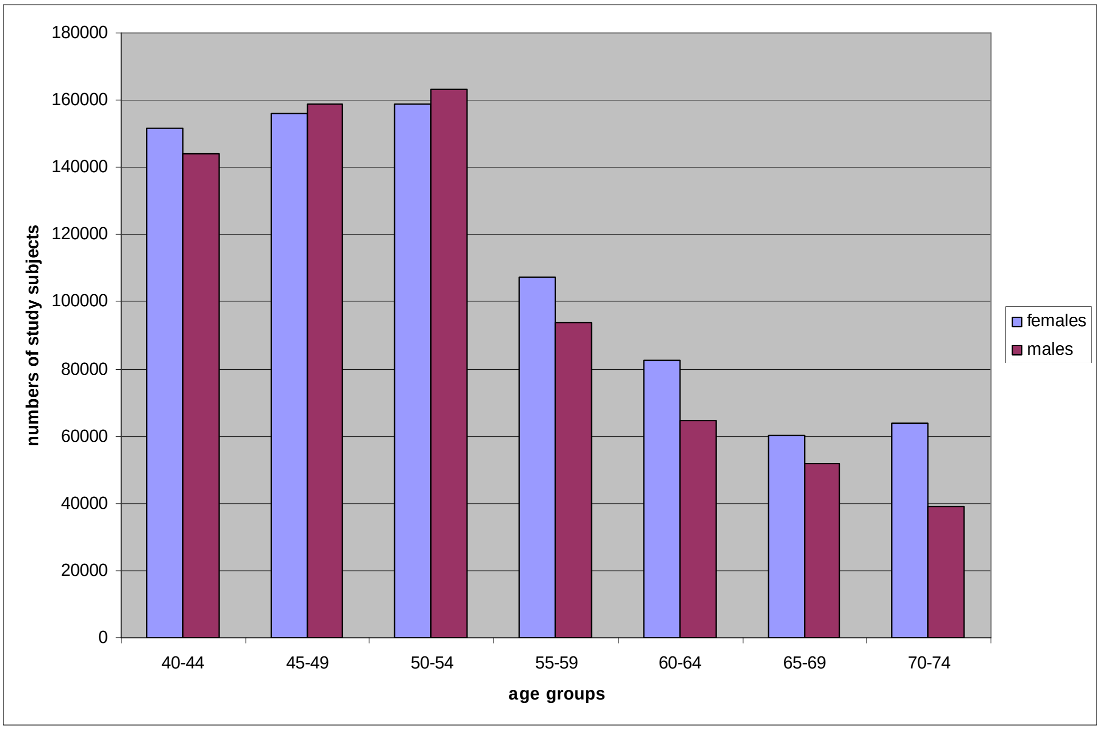


**Figure 2: Percentage of females in each BPG within each age group – population weights used.**

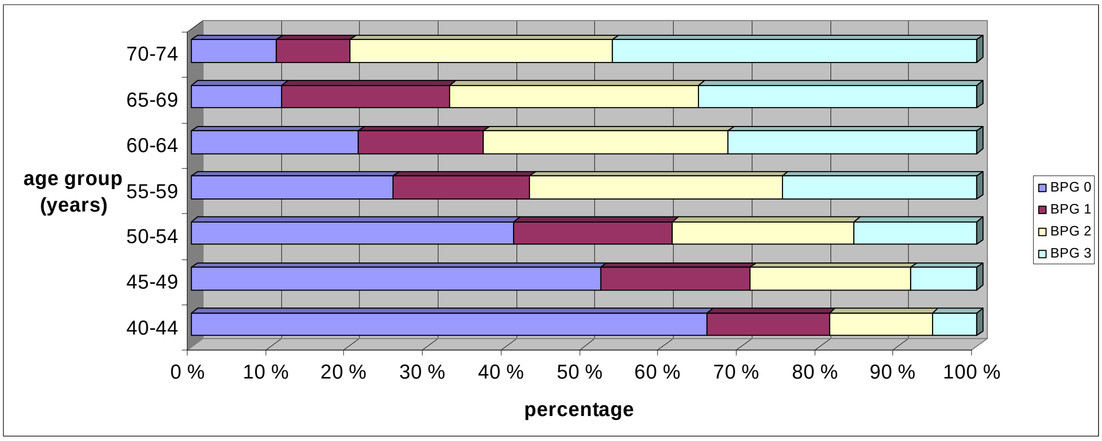


**Figure 3: Percentage of males in each BPG within each age group – population weights used**

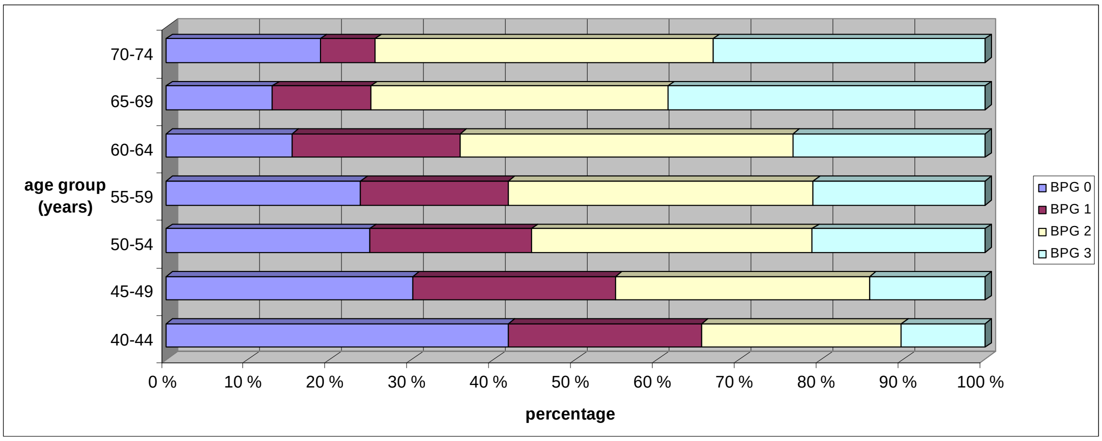

Supplement: Additional File 6 — Population sizes. Supplementary details concerning the size of the study and target populations. [file 1472-6963-7-172-S6.pdf]
