# Supplementary material for: Cost-effectiveness analysis of guidelines for antihypertensive care in Finland
Source: BMC Health Serv Res. 2007 Oct 24;7:172. doi: 10.1186/1472-6963-7-172 (PMC2174470; doi:10.1186/1472-6963-7-172)
Supplement: Additional File 7 — Transition probabilities. Supplementary details concerning transition probabilities. [file 1472-6963-7-172-S7.pdf]

Figure 1: Size of age groups used in the forming transition probabilities, by gender.

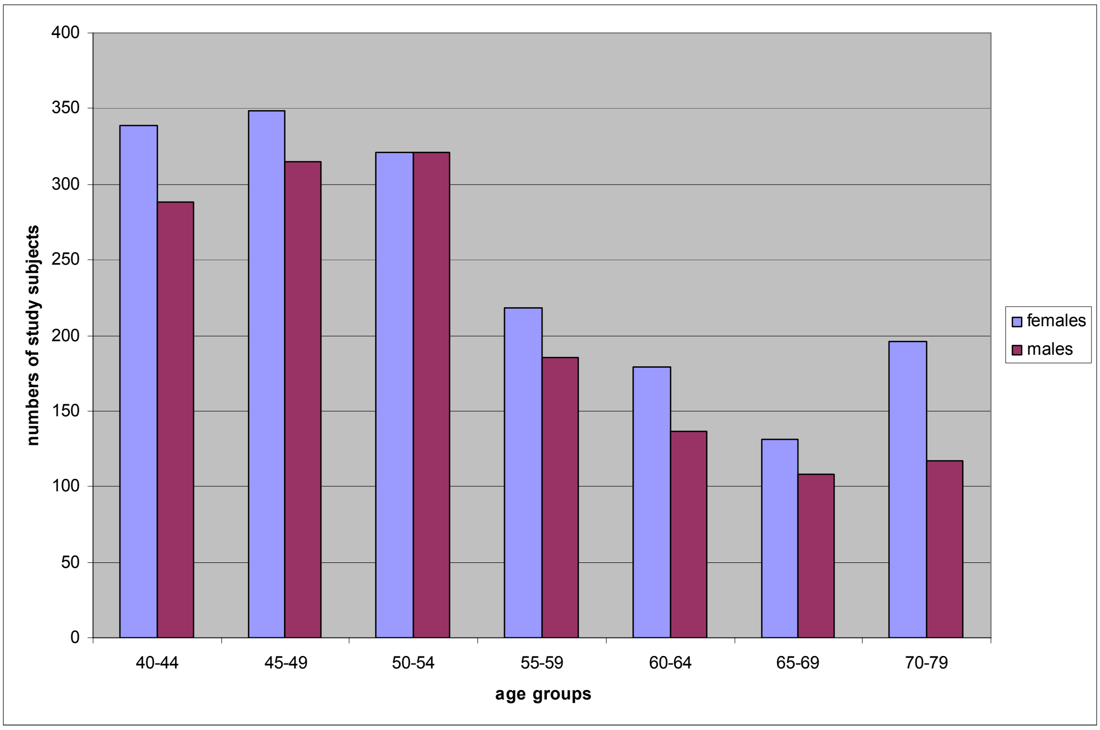

Table 1: An example showing 4 of the 504 sets of transition probabilities between BPG states (16 transitions in each set).

**monitoring group transitions**	females	from↓ / to→	BPG0	BPG1	BPG2	BPG3
**(for age group 45-49)**		BPG0	0.94	0.06	0	0
		BPG1	0	0.818	0.182	0
		BPG2	0	0	0.929	0.071
		BPG3	0	0	0	1
	males	from↓ / to→	BPG0	BPG1	BPG2	BPG3
		BPG0	0.839	0.161	0	0
		BPG1	0	0.756	0.244	0
		BPG2	0	0	0.909	0.091
		BPG3	0	0	0	1
**ACCG triple therapy transitions**	females	from↓ / to→	BPG0	BPG1	BPG2	BPG3
**(for age group 45-49)**		BPG0	1	0	0	0
		BPG1	1	0	0	0
		BPG2	0.718	0.282	0	0
		BPG3	0	0.34	0.588	0.072
	males	from↓ / to→	BPG0	BPG1	BPG2	BPG3
		BPG0	1	0	0	0
		BPG1	1	0	0	0
		BPG2	0.737	0.263	0	0
		BPG3	0	0.27	0.617	0.113

Table 2: An example showing the main transition probabilities used (in this case under the PCP scenario, triple therapy, for a 45-49 age group, after the first Markov cycle)

females	from↓ / to→	BPG0	BPG1	BPG2	BPG3	CHD	CV	CHD death	CV death	Other death
	**BPG0**	0.972	0	0	0	0.013	0.008	0.0015	1.5E-10	0.005
	**BPG1**	0.964	0	0	0	0.016	0.010	0.0019	1.5E-10	0.008
	**BPG2**	0.458	0.434	0.065	0	0.018	0.011	0.0020	1.5E-10	0.012
	**BPG3**	0	0.064	0.674	0.195	0.024	0.017	0.002	0.006	0.018
	**CHD**	0	0	0	0	0.836	0.041	0.004	0.078	0.041
	**CV**	0	0	0	0	0.061	0.819	0.009	0.082	0.029
males	**from↓ / to→**	**BPG0**	**BPG1**	**BPG2**	**BPG3**	**CHD**	**CV**	**CHD death**	**CV death**	**Other death**
	**BPG0**	0.944	0	0	0	0.010	0.010	7.7E-07	0.013	0.023
	**BPG1**	0.931	0	0	0	0.013	0.013	7.8E-07	0.015	0.029
	**BPG2**	0.468	0.401	0.057	0	0.014	0.014	7.8E-07	0.015	0.031
	**BPG3**	0	0.018	0.701	0.161	0.031	0.031	0.007	0.015	0.037
	**CHD**	0	0	0	0	0.871	0.006	0.010	0.084	0.029
	**CV**	0	0	0	0	0.017	0.866	0.040	0.004	0.073
