# Supplementary material for: Cost-effectiveness analysis of guidelines for antihypertensive care in Finland
Source: BMC Health Serv Res. 2007 Oct 24;7:172. doi: 10.1186/1472-6963-7-172 (PMC2174470; doi:10.1186/1472-6963-7-172)
Supplement: Additional File 8 — Main assumptions in this study. Supplementary details of the main assumptions used in this study. [file 1472-6963-7-172-S8.pdf]

**concerning pharmacological therapies**	It is assumed in the base case that neither elevated blood pressure *per se* (SBP exceeding 130 mmHg or DBP exceeding 85 mmHg) nor prolonged antihypertensive therapy will result in direct reductions in health status.<br>In the base case the majority (60%) of patients receive thiazide diuretics as their initial form of treatment, i.e. as the first line drug.<br>The effectiveness of individual pharmacological therapies (i.e., the changes resulting from interventions in terms of change in BP) is assumed not to differ between the ACCG and PCP scenarios. Differences in the interventions' immediate or delayed impact were assumed in the base case to be negligible, and the effects of all pharmacological and lifestyle interventions were assumed to occur within the first Markov cycle.<br>We assume there to be no clinically meaningful differences between the alternative drug classes, given the uncertainty over the size of these effects (see, e.g., [1]). Equivalence, in terms of side-effects, of the pharmacological therapies is assumed given a lack of strong evidence to the contrary. We assume the impact of potential side-effects on antihypertensive care itself or on individuals treated with antihypertensive interventions to be negligible. This assumption is made with the knowledge that, potentially, small changes in HRQL or adverse events may have a large impact on the estimated scenario outcomes [2, 3].<br>It was decided that the base case scenario would assume an ideal situation whereby the adherence of both patients and health care professionals would be complete (i.e., 100% adherence). Sensitivity analysis is undertaken by varying the estimates of BP change following pharmacological interventions; this can be interpreted as a method for describing model sensitivity to level of adherence. Baseline BP changes are assumed to be reduced by, e.g., 50%. Changes in the estimates of BP change following pharmacological interventions do not take into account potential differences in adherence between the two scenarios.<br>It is assumed here that antihypertensive pharmacological therapy is effective in reducing morbidity and mortality through its effect on BP rather than through any potential direct effect on morbidity and mortality.<br>It is currently assumed that the possible switching of regimens allowed under the ACCG scenario within the first Markov cycle does not affect the overall cost of the ACCG pharmacological intervention, and that the cost of the pharmacological intervention under the ACCG scenario is determined by the relevant usage defined at the entrance to each Markov sub-tree.
**concerning estimates of effects**	The BP values for individuals from the H2000 study population are adjusted to incorporate the potential effect of existing antihypertensive medication. We assume that individual BP measurements observed in the H2000 sample population do not fully incorporate reductions in BP resulting from the recorded use of antihypertensive pharmacological interventions. We therefore make an adjustment to such BP measurements according to Table 6, in Additional file 1.<br>We assume, for the purposes of calculating transition probabilities, that the individuals interviewed in the H2000 survey in the age group 75-79 are also representative of the 70-74 year old age group to which we refer throughout the rest of this study.

	The intensity rate estimates were calculated using an assumption that BP levels (standardised for age) fully explain the observed variation in morbidity and mortality, i.e., we conduct the base case analysis using the assumption of 'full benefitt' [2].<br>We assume here that the approximate parity of outcomes for the observed span of time (1980-1994) extends to future periods (e.g., 2001 onwards).<br>The potential influence of long-term (or secular) change in BP values or classification is also a potential confounding factor that has not been incorporated into this study. BP classification errors are assumed not to be a major confounding factor in the CEA study conducted here, even though there is a detectable round number bias.<br>We also assume that the estimated change in BP is actually achieved, that the effect of non-adherence is negligible, that the change to (or inclusion in) a BP group completely and linearly defines risk of morbidity and mortality, and that fraction of benefit does not alter with age.
**concerning treatment under the PCP and ACCG scenarios**	It was assumed that lifestyle intervention is available only as part of the list of intervention options under the ACCG scenario.<br>Treatment is assumed to continue unaltered after the first five-year period, thus limiting the scope for the model to consider, e.g., treatment termination.<br>According to the PCP scenario, it was assumed that pharmacological therapy would continue unaltered after initiation. According to the ACCG scenario, it was assumed that shifts between regimen types could occur, as well as the addition of other drug classes within the first quinquennium. The probability of switching to an alternative regimen (or set of regimens) during the first year of a five-year Markov cycle under the ACCG scenario is, in the base case, 0.45. The potential use of titration was not addressed in either scenario.
**concerning the discount rate**	The discount rate of 5% for both costs and effects is that recommended in the 2003 annex to the decree of the Ministry of Social Affairs and Health concerning the Health Insurance Act, Section 5a.
**concerning equity**	The aggregated ICER results presented here assume that individuals and groups are treated equally according to *ex ante* equity concerns, i.e. that equity is restricted to 'equitable efficiency' [4].

References used in the list of assumptions:

1. National Collaborating Centre for Chronic Conditions: **Hypertension: management of hypertension in adults in primary care: partial update**. 2006.
2. Weinstein MC, Stason WB: **Hypertension: a Policy Perspective**. Cambridge: Harvard University Press; 1976.
3. Drummond M, Coyle D: **Assessing the economic value of antihypertensive medicines**. *J Hum Hypertens* 1992, **6**(6):495-501.
4. Dowie J: **Why cost-effectiveness should trump (clinical) effectiveness: the ethical economics of the South West quadrant**. *Health economics* 2004, **13**(5):453-459.
